# Characteristics of Bone Turnover in the Long Bone Metaphysis Fractured Patients with Normal or Low Bone Mineral Density (BMD)

**DOI:** 10.1371/journal.pone.0096058

**Published:** 2014-05-01

**Authors:** Christoph Wölfl, Daniela Schweppenhäuser, Thorsten Gühring, Caner Takur, Bernd Höner, Ulrich Kneser, Paul Alfred Grützner, Leila Kolios

**Affiliations:** 1 Department for Traumatology and Orthopaedic Surgery, BG Traumacenter Ludwigshafen, Ludwigshafen, Germany; 2 SRH Hochschule Heidelberg, Dpt. Of Social Sciences and Law, Heidelberg, Germany; 3 Department for Plastic-, Reconstructive and Handsurgery, Burn Care Centre, Department of Plastic Surgery of Heidelberg University, BG Traumacenter Ludwigshafen, Ludwigshafen, Germany; Faculdade de Medicina Dentária, Universidade do Porto, Portugal

## Abstract

The incidence of osteoporotic fractures increases as our population ages. Until now, the exact biochemical processes that occur during the healing of metaphyseal fractures remain unclear. Diagnostic instruments that allow a dynamic insight into the fracture healing process are as yet unavailable. In the present matched pair analysis, we study the time course of the osteoanabolic markers bone specific alkaline phosphatase (BAP) and transforming growth factor β1 (TGFβ1), as well as the osteocatabolic markers crosslinked C-telopeptide of type-I-collagen (β-CTX) and serum band 5 tartrate-resistant acid phosphatase (TRAP5b), during the healing of fractures that have a low level of bone mineral density (BMD) compared with fractures that have a normal BMD. Between March 2007 and February 2009, 30 patients aged older than 50 years who suffered a metaphyseal fracture were included in our study. BMDs were verified by dual energy Xray absorptiometry (DXEA) scans. The levels of BTMs were examined over an 8-week period. Osteoanabolic BAP levels in those with low levels of BMD were significantly different from the BAP levels in those with normal BMD. BAP levels in the former group increased constantly, whereas the latter group showed an initial strong decrease in BAP followed by slowly rising values. Osteocatabolic β-CTX increased in the bone of the normal BMD group constantly, whereas these levels decreased significantly in the bone of the group with low BMD from the first week. TRAP5b was significantly reduced in the low level BMD group. With this work, we conduct first insights into the molecular biology of the fracture healing process in patients with low levels of BMD that explains the mechanism of its fracture healing. The results may be one reason for the reduced healing qualities in bones with low BMD.

## Introduction

Bone formation markers reflect the enzymatic activity of bone building cells, mostly osteoblasts, which are excess products from the bone formation process that are released during the breakdown of matrix components [1]. Serum osteocalcin (OC), bone specific alkaline phosphatase (BAP), transforming growth factor β1 (TGFβ1) and the N-terminal propeptide of type-I-collagen (Col1a1) represent the most sensitive bone formation markers. Bone resorption markers are products from the deposition of bone tissue, such as the crosslinked C-(CTX) and N-(NTX) telopeptides of type-I-collagen, or indicate the activity of bone catabolic cells, such as osteoclasts. The serum band 5 tartrate-resistant acid phosphatase (TRAP 5b) as one example is a safe and sensitive marker for detecting the high turnover typical in osteoporosis [2]. In the osteoporotic (non fractured) bone, the microarchitecture deteriorates as a result of altered bone turnover and is characterized by typical changes of these BTMs.

Typically, osteoporosis is a silent disease until the first fracture occurs. After an osteoporotic fracture, callus- and bone remodeling is required [3], [4]. Bone formation and resorption rates increase to repair the bone defect, and the accelerated bone metabolism can be detected using elevated serum levels of BTMs [5]–[11]. The elevation in BTMs may result from increased bone remodeling activity at the fracture sites or reflect accelerated bone resorption close to the fracture [12]–[15]. However, until now, the exact metabolism processes at play during the healing of fractured bone that has a low level of bone mineral density has not been elucidated. Diagnostic instruments that allow a dynamic insight into the fracture healing process and that may be used in collecting decision criteria for treatment of fractures and for monitoring the healing course are as yet unavailable [16].

There are only a few studies examining the characteristics of BTM levels during the healing of osteoporotic fractures [16], [17], and there is no matched pair-analysis comparing the early (up to 8 weeks after fracture) time course of BTM levels during the fracture healing of bones with a low level of bone mineral density versus normal BMD bone. In the present matched-pair study, we analyse the time course of the osteoanabolic markers BAP and TGFβ1 (transforming growth factor β1), as well as the osteocatabolic markers β-CTX and TRAP5b, during the fracture healing process of bone with a low level of bone mineral density compared with fractures of bones with normal bone mineral density.

## Materials and Methods

Between March 2009 and February 2011, patients older than 50 years who suffered a metaphyseal fracture (distal radius, proximal humerus and proximal femur fracture) that required surgical stabilization and who agreed to take part in this special protocol were included in this study. All patients underwent state of the art operative treatment. Exclusion criteria were polytrauma, significant soft tissue injury, highly open fracture, >24 h mechanical ventilation after surgery, dialysis, long-term therapy with immunosuppressants, collagenosis, chronic inflammatory bowel disease, hematological disorders and malignancies.

In all patients, radiographs of the fracture region and the lumbar spine were performed with anterior-posterior and lateral views. Radiographs of the lumbar spine were used to exclude the possibility of morphological changes of the vertebrae that may lead to false values in the DEXA scans for bone density measurement. To monitor the clinical course of fracture healing, radiographs of the fracture site were obtained at 4 and 8 weeks and one year after osteosynthetic stabilization.

Out of this collective, 15 matched pairs were created according to the matching criteria presented in Table 1 of [26, 27]. Among these matched pairs were 7 pairs with fractures of the distal radius, 3 pairs with proximal humeral fractures and 5 pairs with proximal femoral fractures (including femoral neck and intertrochanteric fractures). The patients’ baseline demographic data showed no significant differences (Table 2).

**Table 2:** Table of demographic data of the included and matched patients.

	mean		standard deviation		p-values (t-test)
parameter	normal BMD	low BMD	normal BMD	low BMD	
age	63.40	66.0	5.27	6.11	0.226
BMI (kg/m^2^)	26.01	27.11	5.27	4.75	0.556
weight (kg)	73.37	76.13	18.39	12.17	0.631
height (cm)	168.87	168.53	9.21	7.31	0.913
creatinin (mg/dl)	0.7613	0.78	0.145	0.093	0.678

There were no significant differences in base line demographics. Statistics were performed using the software SPSS 11.0.0 (IBM Germany, Munich, Germany), P≤0.05 was considered to be significant.

The study was designed as an epidemiologic case-control study. After receiving results from the DEXA measurements, patients were matched in a low bone mineral density group and a normal bone mineral density group. Further matching criteria were age (+/−5 years), sex (male, female), fracture localization (proximal humerus, distal radius, proximal femur), fracture type (according to AO-classification; A-, B- or C-type fractures) and operation method (plate, intramedular nail). Thus, we received a collective of 15 matched pairs, or 30 patients in total (Table 1 of [26, 27]), out of a total of 78 patients who met the inclusion criteria. All appropriate patients were included; there were no patients selectively included to match with previously included patients. We did not subdivide groups further because all patients were older than 50 years and represented the collective of patients at risk for osteoporosis and osteoporotic fractures.

The healing of fractures was assessed clinically and radiologically during the usual follow-up examinations that are planned at the fourth and eighth week after surgery, as well as one year later. The fracture consolidation was scored by 2 members of our departments (1 orthopedic surgeon, 1 radiologist) who did not participate in the study and who were blind to the group assignments. Fracture consolidation was scored by the Lane-Sandhu Score (Table 3). In cases where there were post-surgical complications, patients were seen more frequently in the osteoporotic fracture care center in the outpatient clinic of our department.

**Table 3:** Fracture consolidation was scored according to the Lane-Sandhu Score by 2 independent examiners.

Points	Callus and Fracture line
**0**	no callus tissue, fracture line clear
**1**	25% callus tissue, fracture line still clearly visible
**2**	50% callus tissue, fracture line blurred
**3**	75% callus tissue, fracture line barely visible
**4**	100% callus tissue, no remaining fracture line visible

The score provides 5 levels of consolidation. The results are shown in Figure 5.

The bone mineral density (BMD) was examined within one week after surgery by a standardized protocol at the lumbar spine and both femoral necks except for the patients with fractures of the hip (in which case, the injured side was not examined) by DEXA (Lunar iDPX, GE Medical Systems Germany, Solingen, Germany), based on Encore TM Version II.X software. Using normative data for young adult white people, the BMD was categorized as normal or low, as defined by the World Health Organization^17^. Participants with a T-score ≤2.5 SD were categorized as having osteoporosis.

A laboratory analysis was performed the morning after injury with the patient in a fasting state. In addition to the typical routine laboratory values, the bone formation markers BAP (bone specific alkaline phosphatase) and TGFβ1 (transforming growth factor β1), as well as the osteocatabolic markers β-CTX (crosslinked C-(CTX) telopeptide of type-I-collagen) and TRAP5b (serum band 5 tartrate-resistant acid phosphatase), were examined. Quantitative measurements were obtained using the chemiluminescence immunoassay “Access Ostase assay 37300” (Beckman Coulter, Brea, USA) and the electrochemiluminescence immunoassay ECLIA (Roche, Basel, Switzerland). Laboratory tests were repeated at the first, fourth and eighth postoperative week, as well as one year postoperatively.

Statistics were performed using the software SPSS 11.0.0 (IBM Germany, Munich, Germany) and Microsoft Excel 2003/2007 (Microsoft Corp. Washington, USA). By pretrial power analysis of the first three patients, we calculated that 15 matched pairs were necessary for obtaining statistical significance. The Friedman test, the Wilcoxon signed rank test and Mann-Whitney U tests were used as post-hoc tests.

**Figure 1:**
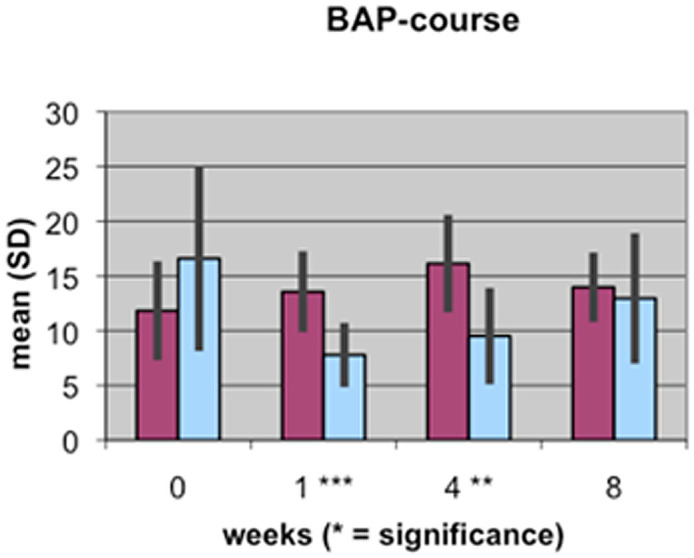
Course of BAP serum concentration during fracture healing of eight weeks in low BMD versus normal BMD patients. Statistics were performed using the software SPSS 11.0.0 (IBM Germany, Munich, Germany), P≤0.05 was considered to be significant, p≤0.01 as very significant, and p≤0.001 as highly significant. Different levels of significance are marked by one to three stars.

**Figure 2:**
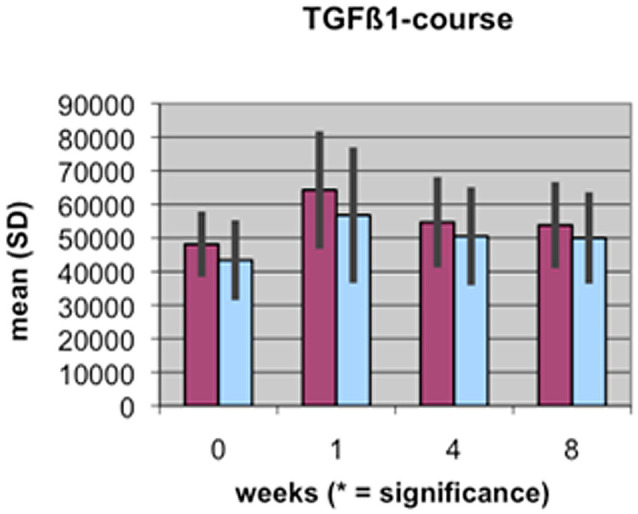
Course of TGFβ1 serum concentration during fracture healing of eight weeks in low BMD versus normal BMD patients. Statistics were performed using the software SPSS 11.0.0 (IBM Germany, Munich, Germany), P≤0.05 was considered to be significant, p≤0.01 as very significant, and p≤0.001 as highly significant. Different levels of significance are marked by one to three stars.

**Figure 3:**
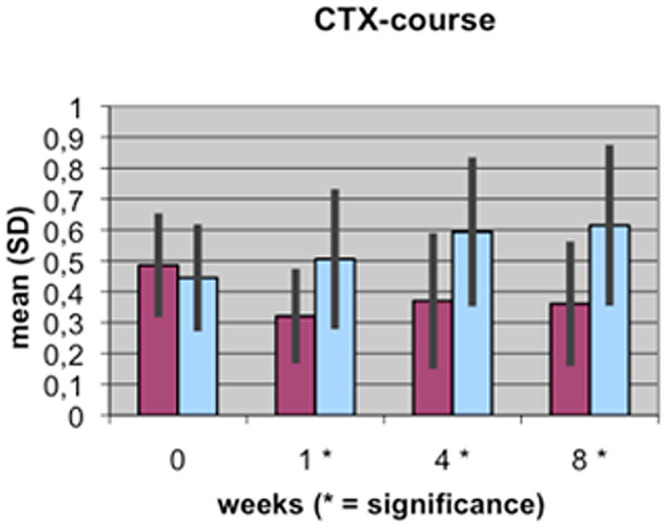
Course of CTX serum concentration during fracture healing of eight weeks in low BMD versus normal BMD patients. Statistics were performed using the software SPSS 11.0.0 (IBM Germany, Munich, Germany), P≤0.05 was considered to be significant, p≤0.01 as very significant, and p≤0.001 as highly significant. Different levels of significance are marked by one to three stars.

**Figure 4:**
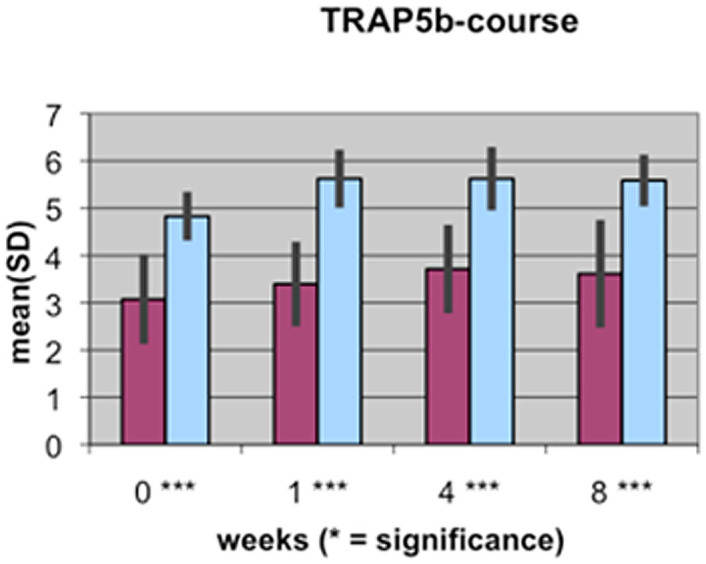
Course of TRAP5b serum concentration during fracture healing of eight weeks in low BMD versus normal BMD patients. Statistics were performed using the software SPSS 11.0.0 (IBM Germany, Munich, Germany), P≤0.05 was considered to be significant, p≤0.01 as very significant, and p≤0.001 as highly significant. Different levels of significance are marked by one to three stars.

A value of P≤0.05 was considered to be significant, p≤0.01 very significant, and p≤0.001 highly significant. These different levels of significance are marked by 1 to 3 stars (*) in Figures 1–4.

The study is approved by the local Ethical Committee of the board of Medical Profession of Rhineland-Palatinate (837.368.10/7377). All participants gave their written informed consent, and the data were analyzed anonymously with matching numbers. The study was conducted according to the principles of the declaration of Helsinki.

## Results

### Bone Turnover Markers

The results of the laboratory analyses of BAP, TGFβ1, β-CTX and TRAP5b are presented graphically in Figures 1–4.

The levels of the bone formation marker BAP showed a remarkable difference between the two groups that could be detected at the first time point. The control group showed a higher level of BAP (16.6 µg/l) than osteoporotic patients, who had a value of 11.8 µg/l. In the first week after surgery, a decrease of BAP to 7.8 µg/l was detected in the control group, whereas the level of the low bone mineral density group differed significantly at 13.6 µg/l. Until the fourth week after surgery, the BAP levels in both groups showed a constant increase, with a maximum value of 16.1 µg/l in the low BMD group, which surpassed the maximum value of the control group significantly (9.5 µg/l). Until the eighth postoperative week, a slight decrease was observed in the control group, while the levels of BAP in the low BMD group decreased slightly.

The results of measuring the levels of TGFβ1 revealed an increase in the first week, followed by a slight decrease up to eight weeks in both groups. The measurements of TGFβ1 levels in the patients with low BMD were at all time points higher than those in the patients with a normal BMD, but without significance.

The β-CTX levels were only slightly different between both groups at the first time point (the day after the fracture occurred), with values of 0.49 µg/l in those with low BMD and 0.44 µg/l in the control group. Within the control group, β-CTX levels constantly increased up to the eighth week after surgery, reaching a maximum value of 0.62 µg/l. The values of β-CTX from the control group were always higher during the healing process than the values from the low BMD group. In the control group, CTX levels decreased initially and stayed at a level of approximately 0.3 µg/l.

The level of TRAP5b in both groups showed an initial increase until the first week after the fracture and remained steady at this level up to the eighth week. At all time points, the values of the control group surpassed those of the low BMD group, and this increase was highly significant.

### Fracture Consolidation

**Figure 5:**
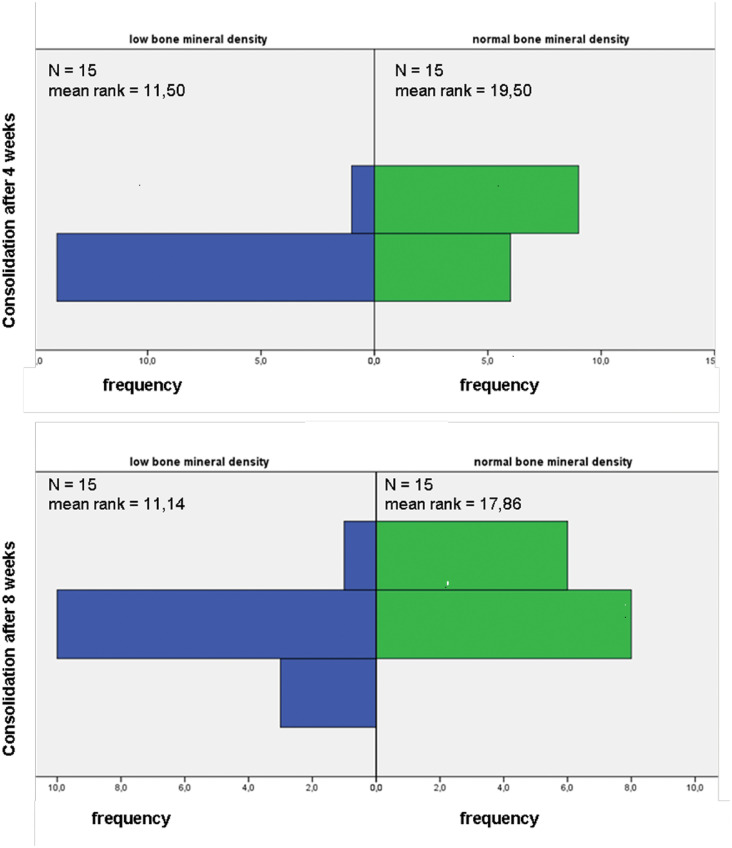
Consolidation of the fractures following the Lane-Shandu-scoring after 4 and 8 weeks, assessed by two independent examiners.

The consolidation was scored using the Lane-Sandhu Score (Table 3). There was a mean score of 2.6 after four weeks in the control group and 3.43 after eight weeks. In the groups of patients with a low level BMD, the score after four weeks was 2.07 and after eight weeks was 2.86. At both time points, there was a significantly reduced score in the low level BMD group (Figure 5). The normal BMD level group showed better callus production, which is a sign of a better fracture healing. None of the patients developed pseudarthrosis. Twice, an endoprosthesis had to be implanted after a failure of the primary osteosynthesis.

## Discussion

Because osteoporosis represents one of the increasing health problems in the world, it is important to examine the mechanisms underlying the qualitatively and quantitatively reduced healing processes [17], [18] of typical metaphyseal fractures in osteoporotic bone.

Bone is remodeled by formation and resorption processes that can be studied by biochemical analyses of the enzymatic activities, degradation products and neosynthesized constituents produced during bone changes [19].

When a fracture occurs, increased resorption and formation are needed to support bone repair and fracture healing [20]. Because bone turnover is increased during fracture healing, biochemical markers of bone metabolism are useful surrogate markers for monitoring fracture healing.

Previous studies showed increased levels of markers of bone resorption in patients with vertebral or hip fractures at an early stage of fracture healing [21], [22], although biochemical markers of bone formation increased at a later stage [23]. Our study showed interesting variations in the levels of the markers of bone metabolism that have not been described in the literature before.

In bone that is of normal BMD, the course of resorption markers β-CTX and TRAP5b showed a slight increase in the first weeks of the fracture healing period and remained at a stable level during the remodeling process in the 4^th^ and up to the 8^th^ week [18], [19], [23]. However, there are significant differences in the levels of bone metabolism markers in bone that is of low BMD. At nearly all time points of the fracture healing process, resorption markers β-CTX and TRAP5b are clearly and significantly reduced compared to bone markers in patients with normal BMD. This could be a result of the disturbed osteoclastic activity in the osteoporotic situation and may be a first explanation of the delayed healing process: the initially necessary bone resorption processes do not occur physiologically (Figures 1, 2).

β-CTX additionally has a high clinical relevance, since it shows very fast and dynamically the changes in bone transforming processes. This marker therewith could be implemented as a special monitoring option for control of therapies. TRAP 5 belongs to the most abundant enzymes in osteoclasts and therefore specific determination of TRAP 5 activity can be essential for clinical assessment of bone metabolism, especially since our results have shown a significant difference to the processes in normal BMD bone.

BAP represents one of the most sensitive markers for bone formation. In our study, the concentration of BAP during the healing process in patients with either low or normal bone density levels shows interesting results. The decrease in BAP in the first week of fracture healing in normal bone is a new finding that is not yet reported in the medical literature. One reason for the decrease in BAP may be that the initial focus of the healing organism is on resorption processes of the bony structure on the side of the fracture (Figure 4). Therefore, resorption markers β-CTX and TRAP5b increase during this first week (Figures 1, 2). BAP is located in the membranes of osteoblasts. In the first week after the fracture, the reactive phase, the amount of soft-tissue cells (endothelial cells, macrophages, granulocytes and fibroblasts) surpasses the number of osteoblasts, perhaps temporarily displacing them, which may explain our findings.

In the patients with low BMD, BAP shows a different concentration curve, with increasing levels up to the 4^th^ week after the injury and then slight decreases until the 8^th^ week of fracture healing. Apart from the first week, BAP levels in the group with low BMD always exceed those in the group with normal BMD, which is partly significant. This result could mean that in bone of normal BMD, there are less active osteoblasts necessary to achieve sufficient fracture healing. More BAP is needed in bone of low BMD and osteoblasts are more activated to balance the remodeling processes in osteoporotic bone (Figure 4).

The chosen second bone formation parameter was TGFβ1, which has been implicated as a regulator of enchondral ossification during skeleton formation, but also during fracture healing. TGFβ is known to be expressed during all stages of fracture repair, and endogenous TGFβ has strong effects on gene expression and differentiation of bone cells during bone repair [24], [25]. Furthermore, in vivo administration of TGFβ induces rapid closure of callus formation in normal bone, and increased bone formation and strength during rat tibial fracture repair of non-osteoporotic [24], [25]. Taken together, these findings illustrate that TGFβ can regulate bone formation cell function in vitro and in vivo, but the roles of endogenous TGFβ in the activities of these cell types and in bone remodeling in a situation of low BMD remained unclear so far.

When considering the evaluated levels of TGFβ1 in our study, the findings are more discrete than the BAP-results, but follow the same structure. At all time points, TGFβ1-activation in bone of patients with low BMD surpasses that of patients with normal BMD. There is a clear increase in the first week of fracture healing that slowly decreases up to the 8^th^ week as the fracture stabilizes (Figure 4). This could be explained as a result of an increased activation of bone formation cells in the osteoporotic situation in order to balance the disturbed microarchitecture especially in the first time period.

Other than the significant differences in bone formation markers between the groups with low BMD and normal BMD, we also found differences in the fracture healing itself as measured by the Lane-Sandhu Scoring System. While the scoring system is inherently subjective, two independent examiners nevertheless rated the patients with low BMD significantly lower in fracture healing than those with normal BMD.

To achieve a more detailed image of these complex processes, further studies are necessary to examine more bone specific markers, as well as fibroblastic and angiogenetic factors. The aim is to establish decision criteria for treatment of fractures and for monitoring osteoporotic fracture healing. These studies are currently in process in our department.

### Limitations

However the study design was planned carefully, the study has some limitations, which were not preventable.

Our study lacks a control group that matches the demographics of individuals without fractures. We had to accept this flaw in our study design because we rarely see patients without fractures in our trauma department.

Additionally, the number of patients included in our study does not reflect the proportion of patients who are treated with metaphyseal fractures in our department. Nearly 60% of the patients asked had no interest in taking part in the study or in further diagnostics, such as DEXA measurements of bone density; by this, we had to accept the use of the matched pair analysis with the included low number of patients.

## Conclusions

With this study, we give first insights to the molecular biology of fracture healing in osteoporotic bone that may explain the mechanisms of fracture healing. The conspicuously elevated osteoanabolic proteins and decreased osteocatabolic proteins during the healing period of patients with low BMD bone compared to patients with normal BMD could be a result caused by disturbed remodeling metabolism and leading to the qualitatively reduced healing process. Further studies are necessary to achieve a more detailed knowledge of fracture healing and the creation of decision criteria for fracture therapy and for monitoring fracture healing.

## Supporting Information

Document S1 Exemplary profile of DEXA and laboratory measurements of one study patient.
(PDF)

Document S2 Positive vote of the Ethical Committee of the board of Medical Profession of Rhineland-Palatinate (837.368.10/7377).
(PDF)
