# Correction: Characteristics of bone turnover in the long bone metaphysis fractured patients with normal or low Bone Mineral Density (BMD)

**DOI:** 10.1371/journal.pone.0270079

**Published:** 2022-06-13

**Authors:** Christoph Wölfl, Daniela Schweppenhäuser, Thorsten Gühring, Caner Takur, Bernd Höner, Ulrich Kneser, Paul Alfred Grützner, Leila Kolios

After this article [1] was published, concerns were raised about similarities between this work and a study published previously in [2]. The two articles address related research questions and present similar questions, and the Methods sections and Table 1 of each article indicate that the studies used overlapping populations. Differences between the two articles include:

- [2] reportedly compares osteoporotic versus nonosteoporotic patients where [1] compares low versus normal bone mineral density patients,
- for pair 15 osteosynthesis is marked as conservative in [2] and as IFN in [1],
- different BAP and CTX results are reported in the two articles, and
- [1] examines outcomes not reported in [2] including TRAP5b, TGFβ1, and fracture consolidation data.

The authors confirmed that these two studies are based on the same populations, and that the *PLOS ONE* study is a follow-up study to [2]. The authors apologize for not having explained this and referenced [2] accordingly in the *PLOS ONE* article [1]. At the time of this notice’s publication, [2] was added to the *PLOS ONE* article’s References section as reference #26. For both studies [1, 2], participants were categorized into two groups according to WHO classification guidelines [3], with participants who had T-score ≤ -2.5 SD categorized for the osteoporotic, or low bone mineral density, group. Terminology used to describe the two groups in [1] versus [2] reflects preferences expressed by reviewers of [1] during the peer review process.

The difference in osteosynthesis information provided for pair 15 in Table 1 of [1] and [2] is due to a reporting error in [2] which has since been corrected [4].

The BAP and CTX results reported in the two articles are based on the same data; underlying data are provided in S1 File. Results reported in [2] included data for 15 patient pairs, of which 11 were included in the study reported in [1]. The authors clarified that the initial experiments reported in [2] assayed only for BAP and CTX. Later, the authors used the same samples, which had been frozen since the original study, to assay for TGFβ1. Reliable TGFβ1 measurements could not be obtained for four samples and those pairs were therefore excluded from the second study; only pairs for which authors were able to obtain a full dataset were included in the analyses reported in [1]. TRAP5b results for the full 15 patient cohort are in Supporting Information (S1 File). The BAP, CTX, and TRAP5b analyses yield the same overall conclusions when comparing the 11 patient versus 15 patient results [1, 2].

The participant recruitment dates are listed incorrectly in the Methods section of [1] and should be March 2007 –February 2009, as is correctly reported in the Abstract.

Bar graphs in the *PLOS ONE* figures did not include legends or information as to measurement units represented on the Y axes. Updated figures are provided here [Figs 1–4]. The p-values (*) reported in the updated Figs 1–4 correspond with the results of the Mann Whitney U test for group differences. Friedmann tests for variation over time points for each marker were performed as reported in the text in [1], but the p-values are not displayed in the figures. p-values are provided here in Supporting Information (S2 File). For each marker (BAP, TGF, CTX, TRAP), the Mann-Whitney U Test was used for group differences and the Friedmann test was used for repeat measurements (time points). The authors stated that non-parametric tests were likely used because the data are not normally distributed for all time points and markers, and use of a non-parametric test for the Lane-Shandu-scoring was likely motivated by the assumed ordinal scale level of the variable. The data distribution, including the corresponding figures to confirm the data distribution are provided here in S3 File.

**Fig 1:**
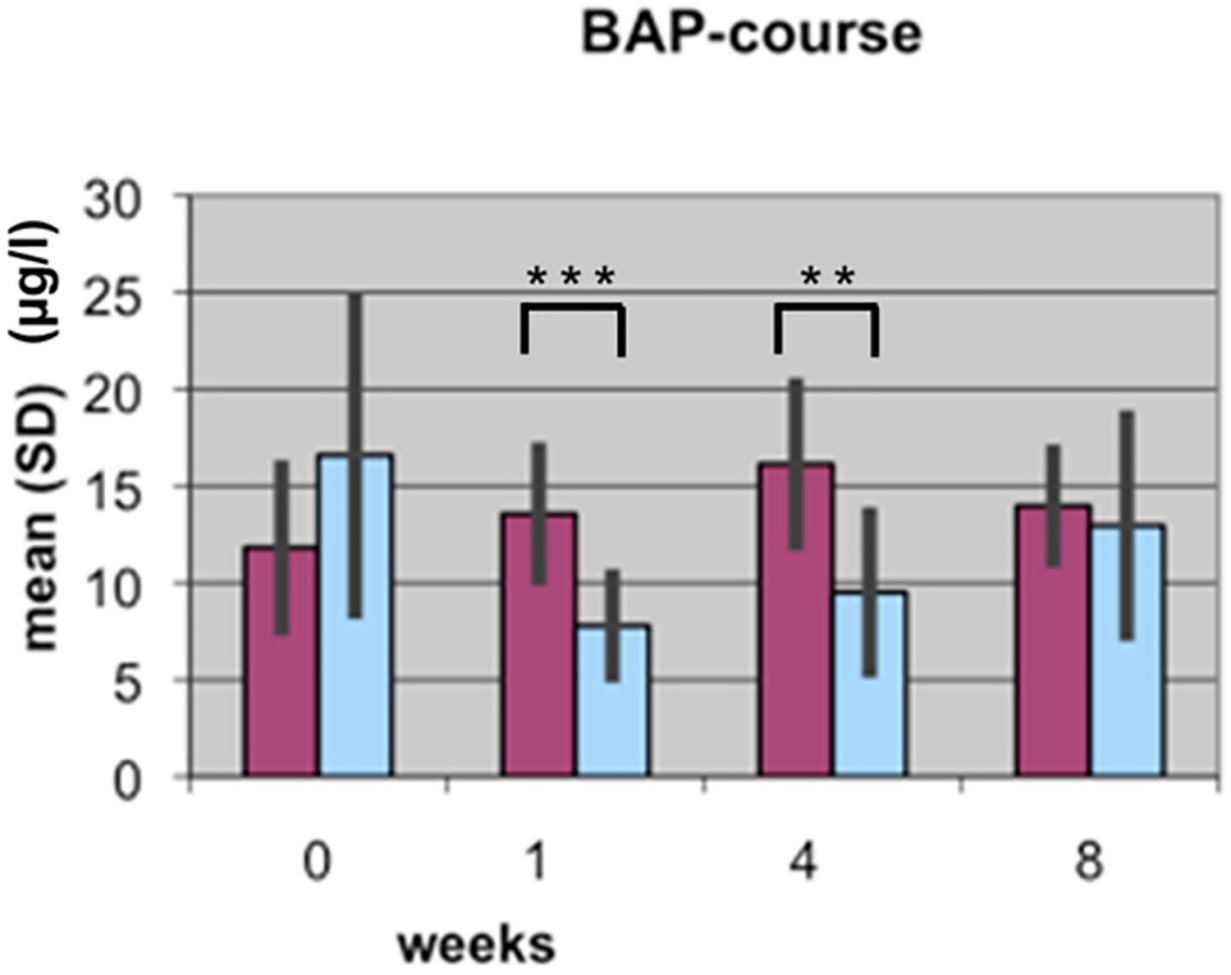
Course of BAP serum concentration (µg/l) during fracture healing of eight weeks in low BMD versus normal BMD patients. (Dark bars: low BMD group, light bars: normal BMD group). Statistics were performed using the software SPSS 11.0.0 (IBM Germany, Munich, Germany), Friedman test, Wilcoxon rank test and Mann-Whitney U tests were used. P ≤ 0.05 was considered to be significant, p ≤ 0.01 as very significant, and p ≤ 0.001 as highly significant. Different levels of significance are marked by one to three stars.

**Fig 2:**
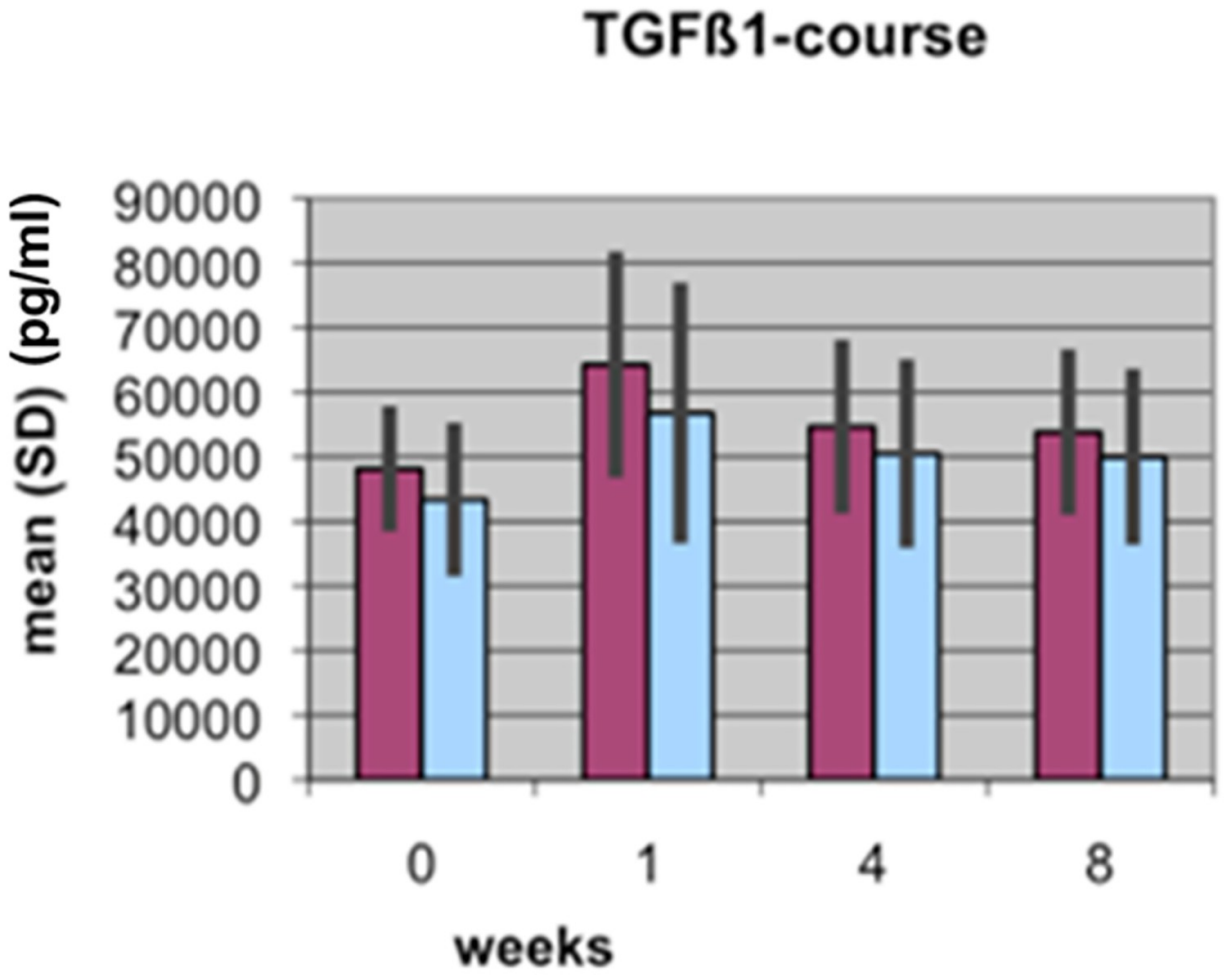
Course of TGFß1 serum concentration (pg/ml) during fracture healing of eight weeks in low BMD versus normal BMD patients. (Dark bars: low BMD group, light bars: normal BMD group). Statistics were performed using the software SPSS 11.0.0 (IBM Germany, Munich, Germany), Friedman test, Wilcoxon rank test and Mann-Whitney U tests were used. P ≤ 0.05 was considered to be significant, p ≤ 0.01 as very significant, and p ≤ 0.001 as highly significant. Different levels of significance are marked by one to three stars.

**Fig 3:**
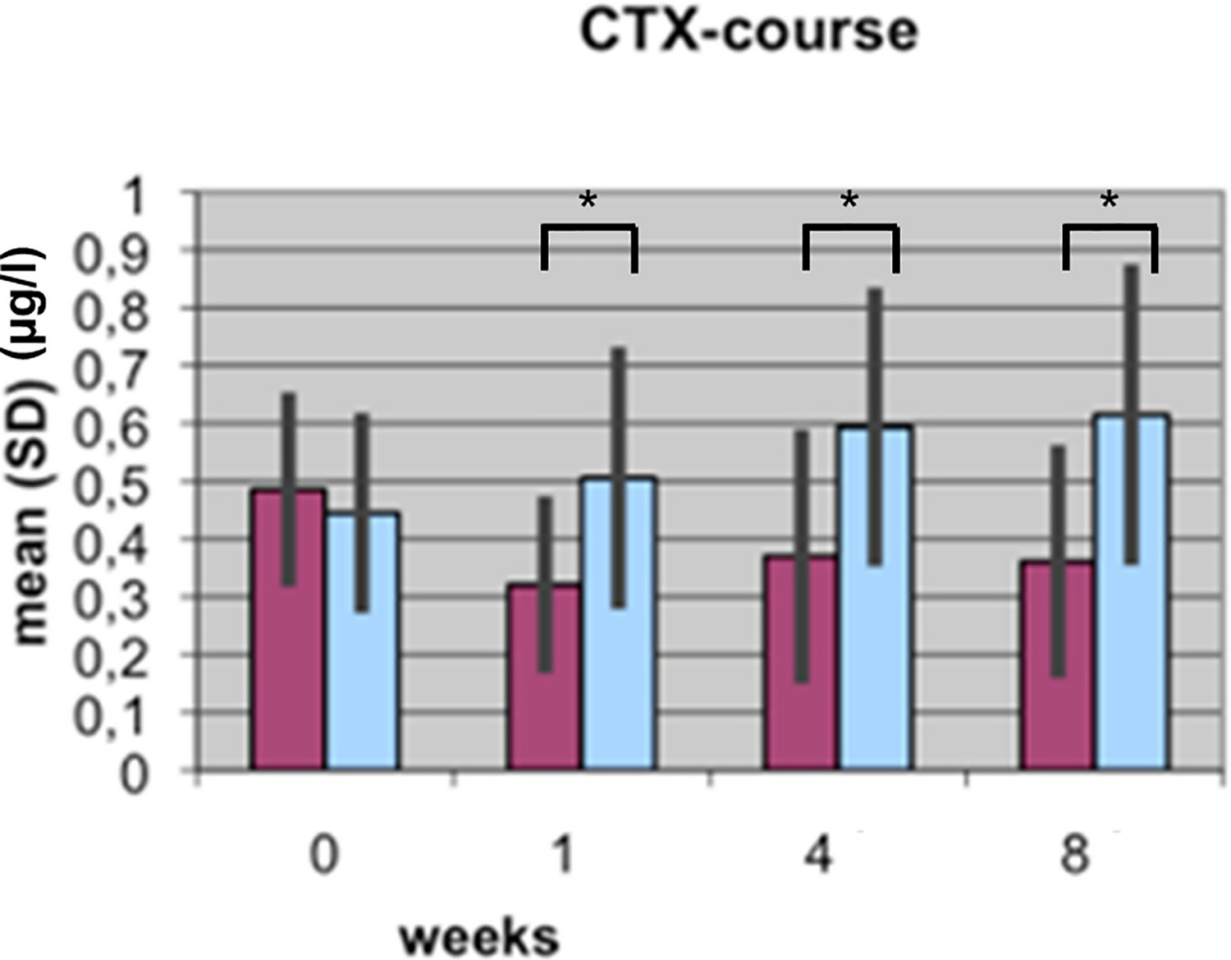
Course of CTX serum concentration (µg/l) during fracture healing of eight weeks in low BMD versus normal BMD patients. (Dark bars: low BMD group, light bars: normal BMD group). Statistics were performed using the software SPSS 11.0.0 (IBM Germany, Munich, Germany), Friedman test, Wilcoxon rank test and Mann-Whitney U tests were used. P ≤ 0.05 was considered to be significant, p ≤ 0.01 as very significant, and p ≤ 0.001 as highly significant. Different levels of significance are marked by one to three stars.

**Fig 4:**
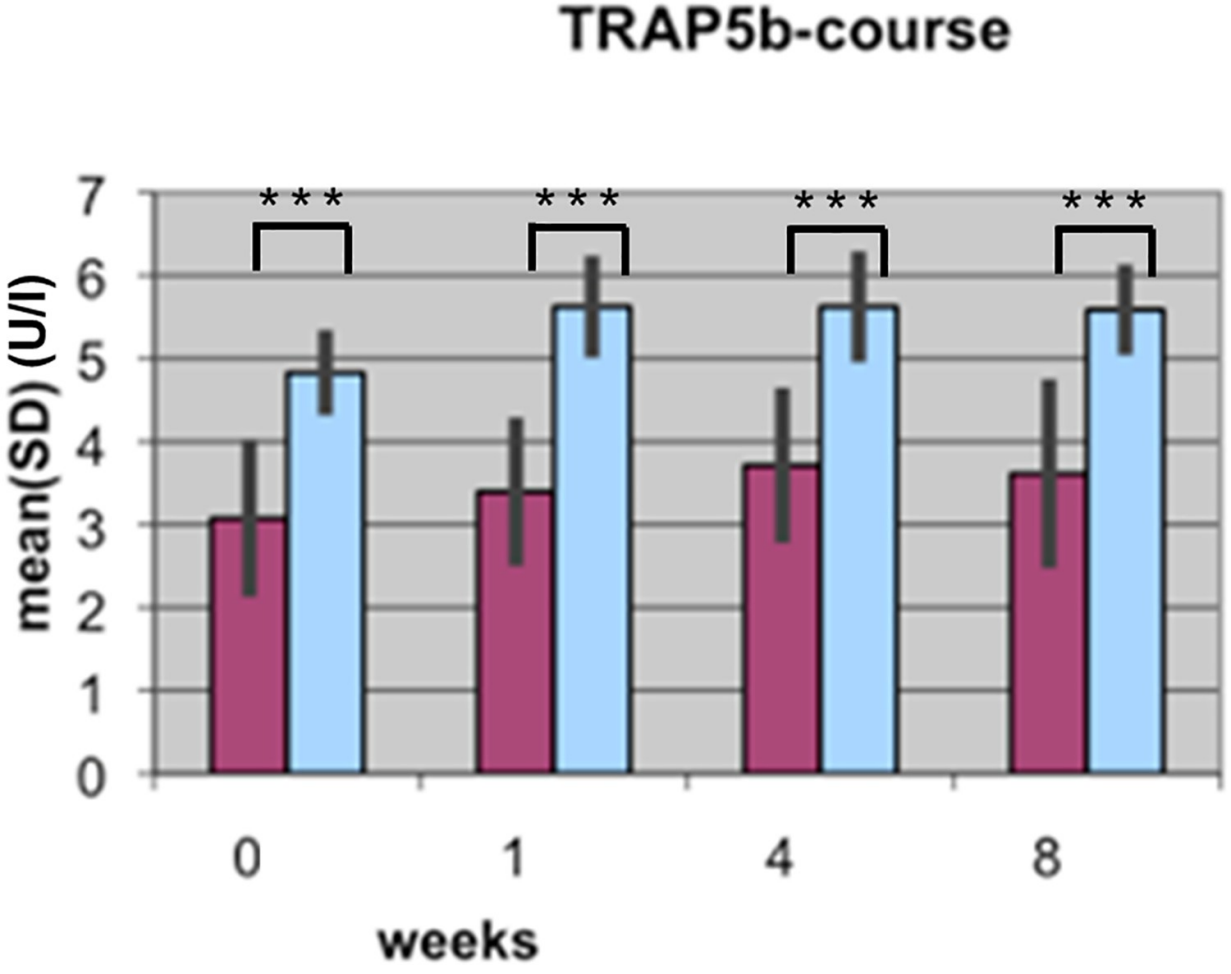
Course of TRAP5b serum concentration (U/l) during fracture healing of eight weeks in low BMD versus normal BMD patients. (Dark bars: low BMD group, light bars: normal BMD group). Statistics were performed using the software SPSS 11.0.0 (IBM Germany, Munich, Germany), Friedman test, Wilcoxon rank test and Mann-Whitney U tests were used. P ≤ 0.05 was considered to be significant, p ≤ 0.01 as very significant, and p ≤ 0.001 as highly significant. Different levels of significance are marked by one to three stars.

The authors have clarified that since the TRA and CTX time point data were not normally distributed, a non-parametric approach for statistical testing was chosen. A Friedman test was performed for each marker and group over repeated measurements. Differences between the two groups were tested via Mann Whitney U test for each marker and time point. Group differences for fracture consolidation scores at two time points were also tested using non-parametric Mann Whitney U tests regarding the ordinal level of the Lane-Shandy score, thus resulting in a total of 26 single tests and the typical problem of alpha inflation. Therefore the statistical approach with multiple single tests is a limitation of this study [1].

The raw data underlying all results reported in the article–including those for which concerns were not raised–are available.

The authors apologize for the errors in the published article.

Table 1 reports material from [2], published in 2012 by Springer, which are not offered under a CC-BY license. At the time of publication of this notice, the article [1] was republished to remove Table 1.

## Supporting information

S1 File Underlying data supporting the TRAP5b, CTX, TGFβ1 and BAP results. (XLS)

S2 File P values for Figs 1–4. (XLSX)

S3 File Data distribution and corresponding figures. (PDF)
