# Supplementary material for: Characteristics of Bone Turnover in the Long Bone Metaphysis Fractured Patients with Normal or Low Bone Mineral Density (BMD)
Source: PLoS One. 2014 May 1;9(5):e96058. doi: 10.1371/journal.pone.0096058 (PMC4006874; doi:10.1371/journal.pone.0096058)
Supplement: Document S1 — Exemplary profile of DEXA and laboratory measurements of one study patient. (PDF) [file pone.0096058.s001.pdf]

Klinik für Unfallchirurgie und Orthopädie BG Unfallklinik

Ludwig-Guttmann-Str.13 D-67071 Ludwigshafen
Tel: 0621-68100

**Patient:**	[redacted]	**Patientenkennung:**	20080723		
**Geburtsdatum:**	[redacted] 61,2 Jahre	**Verantwortlicher Arzt:**	[redacted]		
**Größe / Gewicht:**	168,0 cm 60,0 kg	**Gemessen:**	23.07.2008	17:34:26	(11,40)
**Geschl. / Ethn.:**	Weiblich Weiß	**Analysiert:**	23.07.2008	17:37:16	(11,40)

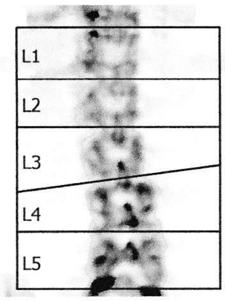

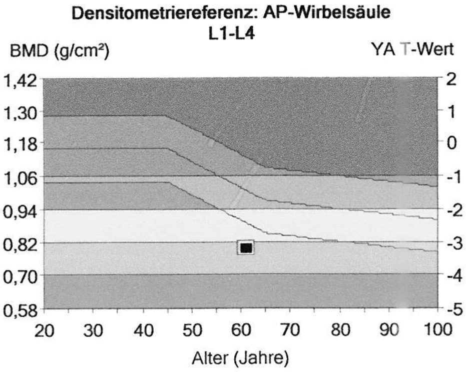

	BMD	Junge Erw.		Altersvergl.	
**Bereich**	**(g/cm²)**	**(%)**	**T-Wert**	**(%)**	**Z-Wert**
L1	0,709	63	-3,5	74	-2,1
L2	0,691	58	-4,2	67	-2,8
L3	0,840	70	-3,0	82	-1,6
L4	0,917	76	-2,4	89	-0,9
L1-L2	0,701	60	-3,9	71	-2,4
L1-L3	0,748	64	-3,5	75	-2,1
L1-L4	0,797	68	-3,2	79	-1,7
L2-L3	0,769	64	-3,6	75	-2,1
L2-L4	0,827	69	-3,1	81	-1,7
L3-L4	0,882	74	-2,6	86	-1,2

Übereinstimmung nach Alter, Gewicht (Frauen 25-100 kg), Ethnische Deutschland (Alter 20-40) AP-Wirbelsäule Referenzbevölkerung (v110)
Laut Statistik sind 68% der Folge-Scans im Bereich von 1SA (± 0,010 g/cm² für AP-Wirbelsäule L1-L4)

Bild nicht für Diagnosezwecke

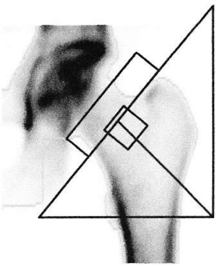

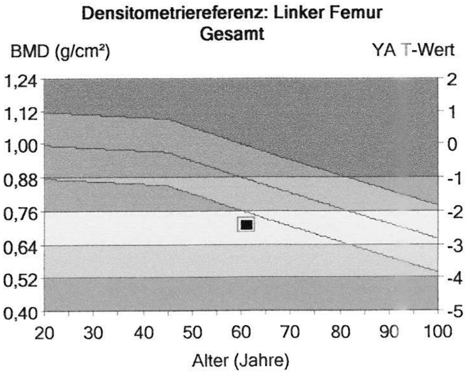

	BMD	Junge Erw.		Altersvergl.	
**Bereich**	**(g/cm²)**	**(%)**	**T-Wert**	**(%)**	**Z-Wert**
Hals	0,696	71	-2,4	85	-1,0
Gesamt	0,706	71	-2,5	81	-1,4

Übereinstimmung nach Alter, Gewicht (Frauen 25-100 kg), Ethnische Deutschland (Alter 20-40) Femur Referenzbevölkerung (v110)
Laut Statistik sind 68% der Folge-Scans im Bereich von 1SA (± 0,012 g/cm² für Linker Femur Gesamt)

Bild nicht für Diagnosezwecke

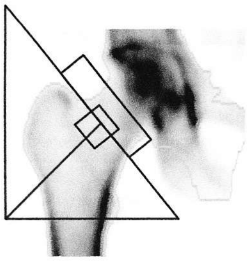

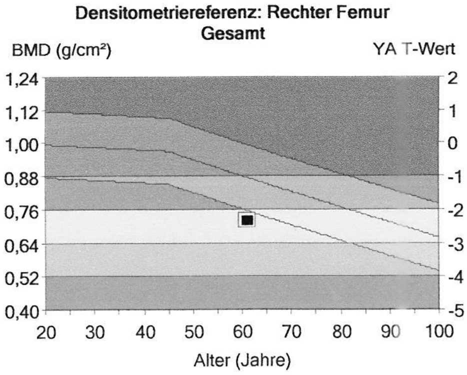

	BMD	Junge Erw.		Altersvergl.	
**Bereich**	**(g/cm²)**	**(%)**	**T-Wert**	**(%)**	**Z-Wert**
Hals	0,699	71	-2,3	85	-1,0
Gesamt	0,718	72	-2,4	82	-1,3

Übereinstimmung nach Alter, Gewicht (Frauen 25-100 kg), Ethnische Deutschland (Alter 20-40) Femur Referenzbevölkerung (v110)
Laut Statistik sind 68% der Folge-Scans im Bereich von 1SA (± 0,012 g/cm² für Rechter Femur Gesamt)

Bild nicht für Diagnosezwecke

Name, Vorname des Versicherten

geb. am

Kassen-Nr.	Versicherten-Nr.	Status

Vertragsarzt-Nr.	VK gültig bis	Datum

LABOR LIMBACH
HEIDELBERG

Im Breitspiel 15 - 69126 Heidelberg
Tel.: 06221/3432-300
Fax: 06221/3432-110
www.labor-limbach.de

Geschlecht:
- ❑ männlich
- ❑ weiblich

Abnahmedatum: ______________

Abnahmezeit: ______________

BG-Unfallklinik Ludwigshafen
Ludwig-Guttmann-Str. 13
67071 Ludwigshafen

Kd.-Nr.: 21715

Station: __________

Kostenträger:
- ❑ privat (Patientenadresse angeben)
- ❑ Krankenhaus

Osteoporose-Diagnostik bei Patienten mit frischen Frakturen
(am Folgetag nach der Fraktur und ggf. bei späteren Kontrollen)

EDV-Nummer	Analysen	benötigtes Material
**90155 Profil Fraktur BG LU**	Pyridinoline, 25-OH-Vit.-D, Östradiol, iPTH, CTX, PINP, Homocystein, Vitamin B_{12}, Folat	1. Morgenurin, 10 ml<br>Serum, 2 ml<br>EDTA-Plasma, 1,5 ml

Materialabnahmehinweise:
1. Morgenurin: Gewinnung direkt nach dem Aufstehen, z. B. 06.30 Uhr
EDTA-Plasma, Serum: Abnahme vor 08.30 Uhr, nüchtern (Proben innerhalb 1 Stunde zentrifugieren und abtrennen)

Material bis zur Abholung kühl lagern.

Datum: __________ Unterschrift: ______________________________

Prof.Dr.med.H.Schmidt-Gayk Dres.med. H.J.Limbach S.Walch J.Bartel H.Kiralp
Fachärzte für Labormedizin
Dres.med.A.Fahr A.Turnwald-M. M.Holfelder **Dr.med.G.Porsch**
Ärzte für Mikrobiologie und Infektionsepidemiologie Fachärztin für Hygiene

Im Breitspiel 15 · 69126 Heidelberg Tel.: 06221/34 32 -0 Fax.: 06221/34 32 110

Dr.med. Wölfl
BG-Unfallklinik / Ambulanz
Ludwig-Guttmann-Straße 13

67071 Ludwigshafen

Patient : [redacted]
Geburtsdatum : [redacted]
Geschlecht : weiblich
Kennung :
Klin.Diagnose:

Kopie : **168 337727** **Seite 1**
Einsender : 21715
Eing/Ausg : 15.08.2008/19.08.2008

Befundbericht

Einsendung von:
BG Unfallklinik Labor
Station UCHI 9 Ludwig-Guttmann-Straße 13 67071 Ludwigshafen

**Untersuchung/Methode**	**Normber.**	**Dim.**	**Wert**

Probe 01: EDTA-Plasma vom 15.08.2008 07:15h / Eingang 15.08.2008 17:42

**Parathormon (PTH) intakt**	11 - 43	**ng/l**	**12**

Bei ungenügender Vitamin D Versorgung (25OHD < 20 ug/l) werden PTH-Spiegel bis 60 ng/l gemessen. Wegen leichter Tagesrhythmik sind die Werte abends etwa 10 ng/l höher als morgens.

Zielbereich bei Dialysepatienten 150 - 300 ng/l (nach K/DOQI-Leitlinien).

Probe 02: Serum vom 15.08.2008 07:15h / Eingang 15.08.2008 17:42

**25-Hydroxy-Vitamin 25(OH)D3**	20 - 70	**ug/l**	**39**

Zielbereich bei Osteoporose und Dialysepatienten 30-40 ug/l (Dawson-Hughes 2004; Coen, Kidney Int.2005; 68; 1840-8 und nach K/DOQI-Leitlinien > 30 ug/l).

Probe 01: EDTA-Plasma vom 15.08.2008 07:15h / Eingang 15.08.2008 17:42

**beta-CrossLaps**	< 0.57	**ug/l**	**0.31**

Der Referenzbereich gilt für eine Probe, die morgens zwischen 8:00 Uhr und 8:30 Uhr beim nüchternen Patienten abgenommen wurde. Dialysepat. zeigen wenig Tagesrhythmik. Dialysepatienten (normaler Knochenabbau): 0,5 - 2,1 ug/l.

Probe 03: Urin vom 15.08.2008 07:15h / Eingang 15.08.2008 17:42

**Pyridinolin im Urin**	160 - 280	**ug/g Krea.**	**304** ↑

Probe 01: EDTA-Plasma vom 15.08.2008 07:15h / Eingang 15.08.2008 17:42

**Prokollagen TypI N Propeptid**	15 - 59	**ug/l**	**50**

Bei Dialysepatienten werden PINP-Werte im Bereich

Prof.Dr.med.H.Schmidt-Gayk Dres.med. H.J.Limbach S.Walch J.Bartel H.Kiralp
Fachärzte für Labormedizin
Dres.med.A.Fahr A.Turnwald-M. M.Holfelder
Ärzte für Mikrobiologie und Infektionsepidemiologie
Dr.med.G.Porsch
Fachärztin für Hygiene

Im Breitspiel 15 · 69126 Heidelberg Tel.: 06221/34 32 -0 Fax.: 06221/34 32 110

Dr.med. Wölfl
[redacted]

BG-Unfallklinik / Ambulanz
Kopie **168 337727** 19.08.2008 **Seite 2**

**Untersuchung/Methode**	**Normber.**	**Dim.**	**Wert**

von 50 - 400 ug/l als Zielbereich angesehen.
(Alvarez et al.,JBoneMinerMetab 2004;22:254-9)

Probe 03: Urin vom 15.08.2008 07:15h / Eingang 15.08.2008 17:42

**Desoxypyridinolin im Urin**	26 - 65	**ug/g Krea.**	83 ↑

Referenzbereiche der Pyridinoline (Pyridinolin und Desoxypyridinolin) nur gültig für die Bestimmung im ersten Morgenurin (Spontanurin).

Befund spricht für mittelgradig erhöhten Knochenabbau. Falls andere Störungen (Tumor, Immobilisierung, Hyperthyreose, Hyperparathyreoidismus, Akromegalie, M. Paget, Vitamin-D-Mangel) ausgeschlossen sind, Östrogen-Gestagen-Substitution in Abhängigkeit von der Klinik und vom Menopausenstatus (LH, FSH, Östradiol) zu empfehlen.

**Kreatinin im Urin**	0.4 - 2	**g/l**	0.75

Probe 02: Serum vom 15.08.2008 07:15h / Eingang 15.08.2008 17:42

**Östradiol, 17-beta- / E2**		**ng/l**	65

Referenzbereich:

Follikelphase	13 - 166 ng/l
Mittzyklischer Gipfel	86 - 498 ng/l
Lutealphase	44 - 211 ng/l
Postmenopause (ohne Östrogen-Sub.)	< 35 ng/l

**Homocystein**		**umol/l**	9.6

Referenzbereiche für Nierengesunde
Optimaler Wert: unter 9 umol/l

Ansteigendes Mortalitätsrisiko bei folgenden Werten:

9.0-14.9 umol/l	1.9 fach	Lit.: NEJMed 1997;
15.0-19.9 umol/l	2.8 fach	337:230-236
über 20.0 umol/l	4.5 fach	

Bei eingeschränkter Nierenfunktion sind Werte unter 20 umol/l anzustreben.

Prof.Dr.med.H.Schmidt-Gayk Dres.med. H.J.Limbach S.Walch J.Bartel H.Kiralp
Fachärzte für Labormedizin
Dres.med.A.Fahr A.Turnwald-M. M.Holfelder
Ärzte für Mikrobiologie und Infektionsepidemiologie
Dr.med.G.Porsch
Fachärztin für Hygiene

Im Breitspiel 15 · 69126 Heidelberg Tel.: 06221/34 32 -0 Fax.: 06221/34 32 110

Dr.med. Wölfl
[redacted]

BG-Unfallklinik / Ambulanz
Kopie **168 337727** 19.08.2008 **Seite 3**

**Untersuchung/Methode**	**Normber.**	**Dim.**	**Wert**
**Vitamin B12**	141 - 489	**pmol/l**	**646** ↑

unter 141 pmol/l kann eine makrozytäre Anämie auftreten,
unter 250 pmol/l ist bei Nierengesunden mit einem erhöhten Homocysteinspiegel zu rechnen.
Bei Dialysepatienten sind zur Senkung des Homocysteinspiegels höhere Vitamin-B12-Spiegel erforderlich (600-800 pmol/l).

**Untersuchung/Methode**	**Normber.**	**Dim.**	**Wert**
**Folsäure (Folat)**	4.5 - 21	**nmol/l**	**22**

unter 4 nmol/l kann eine makrozytäre Anämie auftreten,
unter 14 nmol/l steigt der Homocysteinspiegel beim Nierengesunden.
Bei Dialysepatienten sind höhere Spiegel zur Senkung des Homocysteins erforderlich.

Diensthabende(r) Arzt/Ärzte (Durchwahl): Kiralp (109)

Mit freundlichen Grüßen
