# Supplementary material for: Characteristics of Bone Turnover in the Long Bone Metaphysis Fractured Patients with Normal or Low Bone Mineral Density (BMD)
Source: PLoS One. 2014 May 1;9(5):e96058. doi: 10.1371/journal.pone.0096058 (PMC4006874; doi:10.1371/journal.pone.0096058)
Supplement: Document S2 — Positive vote of the Ethical Committee of the board of Medical Profession of Rhineland-Palatinate (837.368.10/7377). (PDF) [file pone.0096058.s002.pdf]

Landesärztekammer Rheinland-Pfalz
Körperschaft des Öffentlichen Rechts
Postfach 29 26 · 55019 Mainz
Deutschhausplatz 3 · 55116 Mainz
Telefon (06131) 28822-0
Telefax (06131) 28822-66

- Ethik-Kommission -

Landesärztekammer Rhld.-Pf. · Postfach 29 26 · 55019 Mainz

BG Unfallklinik Ludwigshafen
Klinik für Hand-, Plast. und Rek. Chirurgie
Frau Dr. Leila Kolios
Ludwig-Guttmann-Straße 13
67071 Ludwigshafen

Ansprechpartner:

Frau Pierzina, Frau Lettau,
Frau Rademacher, Frau Bohrer

Telefon:
-63 (Pie), -64 (Le), -65 (Rd), -67 (Bo)
Bitte beachten Sie die geänderten Rufnummern!

Bitte bei jedem Schriftwechsel die Bearbeitungsnummer angeben!

Mainz, den 14.12.2010 / Bo

Antrag (monozentrisch) BG UNFALLKLINIK LUDWIGSHAFEN, Klinik für Hand-, Plastische und Rekonstruktive Chirurgie, Ludwigshafen, Dr. L. Kolios, Dr. Wölfl, vom 22.09.2010 (Eingang: 24.09.2010):
"Osteoporotische Frakturen - Verlauf von Wachstumsfaktoren bei osteoporotischen Frakturen und Stimulation der osteoporotischen metaphysären Frakturheilung unter niederfrequenter Ultraschalltherapie sowie extrakorporaler Stoßwellentherapie"

Bearbeitungsnummer 837.368.10 (7377)

Sehr geehrte Frau Dr. Kolios,

die Ethik-Kommission bei der Landesärztekammer Rheinland-Pfalz hat in ihrer Sitzung am 06.10.2010 über Ihren Antrag beraten und folgenden Beschluss gefasst:

Auf der Grundlage der zur Beratung vorgelegten Unterlagen sowie der durch Ihr Schreiben vom 13.11.2010, 03.12.2010 und 11.12.2010 nachgereichten Dokumente bestehen nach dem gegenwärtigen Stand keine berufsethischen und berufsrechtlichen Bedenken gegen die Durchführung der geplanten Studie, d. h. Sie erhalten ein positives Votum.

<u>Ferner gibt die Ethik-Kommission folgende allgemeine Hinweise:</u>

Die Verantwortlichkeit des Prüfarztes bleibt in vollem Umfang bestehen und wird durch diese Entscheidung nicht berührt. Die Entscheidung ergeht unter dem Vorbehalt gleichbleibender Gegebenheiten.

Der Ethik-Kommission sind alle schwerwiegenden Komplikationen in beurteilbarer Form unverzüglich mitzuteilen. Die Ethik-Kommission bittet darum, dass ihr das Ergebnis der Studie zur Kenntnis gebracht wird.

Mit freundlichen Grüßen

i.V. [illegible]

Prof. Dr. Dr. Dick
Stellv. Vorsitzender

Landesärztekammer Rheinland-Pfalz

Folgende Unterlagen haben zur Beratung vorgelegen:

Anschreiben vom 22.09.2010 (eingegangen am 24.09.2010) mit folgenden Anlagen:
Antrag vom 21.09.2010
Einverständniserklärung Klinikdirektor vom 31.08.2010
Curriculum Vitae Dr. Kolios
Curriculum Vitae Dr. Wölfl
Patientengebrauchsanweisung und Packungsbeilage

Anschreiben vom 13.11.2010 (eingegangen am 16.11.2010) mit folgenden nachgereichten Anlagen:
Certificate No. CE 512806
Informationsblatt für Patienten
Einwilligungserklärung

Anschreiben vom 03.12.2010 (eingegangen am 06.12.2010) mit folgenden nachgereichten Anlagen:
Studieninformationsblatt für Patienten
Überarbeitete Einwilligungserklärung

Anschreiben vom 11.12.2010 (eingegangen am 13.12.2010) mit folgenden nachgereichten Anlagen:
Überarbeitetes Studieninformationsblatt für Patienten
Überarbeitete Einwilligungserklärung

Informationsblatt für Patienten
(Zum Verbleib bei den Patienten)

Osteoporotische Frakturen, Korrelation zwischen den humanen Wachstumsfaktoren und Östradiol, Vitamin D und Procalcitonin im Serum von Patienten mit Altersfrakturen ohne adäquatem Trauma.

Sehr geehrte Patientin, sehr geehrter Patient,

Sie haben einen Knochenbruch erlitten und sollen in unserem Haus eine offene Stabilisierung dessen mit Metallimplantaten erhalten. Aufgrund des Unfallmechanismus, der Art des Bruches und der Gesamtkonstellation besteht der Verdacht auf einen osteoporotischen Bruch. Um diesen Verdacht zu bestätigen, bieten wir Ihnen eine spezielle Osteoporosediagnostik an, bestehend aus Laborparametern, messbar in Blut und Urin, sowie bildgebenden Verfahren.

Venöse Blutproben:

Um den weiteren Verlauf der Laborparameter bestimmen zu können, sollte innerhalb von 1Jahr in den Wochen 1, 4, 8 und nach einem Jahr eine Blutentnahme aus der Vene im nüchternen Zustand erfolgen. Diese wird von Ärzten unserer Klinik oder einem Medizinstudenten, der mit unserer Studie vertraut ist vorgenommen. Soweit möglich werden wir diese Blutentnahmen mit den Routinelaborkontrollen kombinieren.

Möglicherweise unerwünschte Effekte dieser Blutentnahme sind sehr ähnlich wie bei Blutentnahme, die normalerweise in der Klinik oder beim Hausarzt durchgeführt werden. Die Menge des Blutes die abgenommen werden sind „4 Röhrchen“ die entspricht ca. 32ml Blut. Erfahrungsgemäß hat dies keinen relevanten Einfluss auf Ihre gesamte Blutmenge. Möglicherweise ungewünschte Nebenwirkungen können sein:

- Schmerzen an der Einstichstellen
- Nachblutung, Hämatom (blauer Fleck)
- Infektion (sehr selten)
- Vagale Reaktion mit niedrigem Blutdruck
- Übelkeit und evtl. Synkope (Ohnmacht, Schwindel, Schwächegefühl)

Morgenurin

Um spezielle Knochenmarker bestimmen zu können, benötigen wir von Ihnen zweimalig eine morgentliche Urinprobe in Woche 1 und 1 Jahr postoperativ.

Bildgebende Verfahren

Um den Verdacht einer Osteoporose zu bestätigen und das Stadium dieser näher bestimmen zu können, erfolt ein Röntgen der Lendenwirbelsäule in zwei Ebenen sowie eine Knochendichtemessung mittels einer DEXA.

Bluterguss aus dem Bruchbereich:

Bei diesem operativen Eingriff wird der Bruchbereich freigelegt und der sich dort befindliche Bluterguss entfernt um die Bruchenden wieder vereinigen zu können. Wir bitten Sie uns zu gestatten, eine kleine Probe dieses Bruchhämatoms, welches normalerweise sowieso entfernt wird, für wissenschaftliche Zwecke zu entnehmen. Hierdurch wird der Fortgang der Operation in keiner Weise beeinträchtigt. Nebenwirkungen sind nicht zu erwarten, ebenso wird der Heilprozess nicht negativ beeinflusst.

Aufbewahrung der Proben:

Es ist zu erwarten, dass Wachstumsfaktoren bzw. Regelmechanismen der Knochenregenration zu untersuchen sind. Demzufolge würden wir gerne nicht benötigte Probenteile für spätere Untersuchungen aufbewahren. Selbstverständlich werden mit diesen Proben ausschließlich Untersuchungen zur Osteoporoseforschung erfolgen.

Im Forschungslabor werden dies Proben untersucht. Ziel ist es eine Erfassung der Wachstumsfaktoren, welche für die normale Knochenheilung notwendig sind mit den untersuchten Faktoren zur Osteoporosebestimmung zu vergleichen.

Die Teilnahme an der Studie/Untersuchung ist freiwillig. Sie können jederzeit, ohne Angabe von Gründen und ohne Nachteile für Ihre Behandlung die Zustimmung zurückziehen.
Bei Rücktritt wird bereits gewonnenes (Daten-) Material vernichte, es sei denn, Sie stimmen zu, das Sie trotz Ihres Rücktritts mit der Auswertung des Materials einverstanden sind.

Die Vorschriften über die ärztliche Schweigepflicht und den Datenschutz werden im Rahmen dieser Studie eingehalten. Es werden ggf. nur anonymisierten Datensätze ohne Namensnennung weitergegeben. Dritte erhalten keinen Einblick in Originalunterlagen.

Mit freundlichen Grüßen

Dr. Christoph Wölfl

Cand. Med. Mirjam Hitzler

Studie

Osteoporotische Frakturen, Korrelation zwischen den humanen Wachstumsfaktoren und Östradiol, Vitamin D und Procalcitonin im Serum von Patienten mit Altersfrakturen ohne adäquatem Trauma.

Osteoporose Sprechstunde
BG Unfallklinik Ludwigshafen
Termine 2007

Notfallambulanz
Uhrzeit 08:00 - 11:00

Bei Blutabnahme bitte 2h vorher keine Mahlzeiten zu sich nehmen.
Terminänderung über 0621 - 68102920

Termine:

	Aufnahmewoche	- Aufklärungsgespräch<br>- Faktormessung<br>- LWS Röntgen
	1. Woche postoperativ	- Osteoporoseparameter<br>- Faktormessung<br>- DEXA Termin
	DEXA Messung	
	4. Woche postoperativ	- Therapiegespräch<br>- Faktormessung<br>- Besprechung der DEXA
	8. Woche postoperativ	- Faktormessung<br>- Radiologische Kontrolle<br>- Klinische Kontrolle
	DEXA Messung	
	1 Jahr postoperativ	- Osteoporoseparameter<br>- Faktormessung<br>- DEXA

Einverständniserklärung

Osteoporotische Frakturen, Korrelation zwischen den humanen Wachstumsfaktoren und Östradiol, Vitamin D und Procalcitonin im Serum von Patienten mit Altersfrakturen ohne adäquatem Trauma.

(An der Studie teilnehmender Patienten)

Ludwigshafen, den _________

Name: ______________________________________

Geburtsdatum: ____________________

Die schriftliche Patienten/Probanden-Aufklärung habe ich erhalten und gelesen. Darüber hinaus bin ich mündlich aufgeklärt worden. Dabei wurden alle meine Fragen beantwortet.

Ich stimme der Teilnahme an der Studie freiwillig zu. Ich weiß, dass ich diese Zustimmung ohne Angabe von Gründen jederzeit und ohne Nachteile für meine weitere medizinische Versorgung widerrufen kann.

Bei Rücktritt von der Studie bin ich mit der Auswertung meines (Daten-) Materials einverstanden

Oja Onein

Ich wurde darüber aufgeklärt und stimme zu, dass die im Rahmen dieser Studie erhobenen Daten in anonymisierter Form dokumentiert und ggf. weitergegeben werden. Dritte erhalten keinen Einblick in Originalkrankenunterlagen.

Ich erkläre mich mit der Entnahme von

Venenblutproben einverstanden ____________________

Ich erkläre mich mit der Aufbewahrung der Proben über den Studienzeitraum hinaus einverstanden ____________________

Dr. ________________________________

Fragebogen zur Erstellung eines Patientenprofils (ID____________________)
Osteoporotische Frakturen, Korrelation zwischen den humanen Wachstumsfaktoren und Östradiol, Vitamin D und Procalcitonin im Serum von Patienten mit Altersfrakturen ohne adäquatem Trauma.

Name ______________
Vorname ______________
Geb. ______________
Geschlecht ______________
Adresse ______________
Ort ______________
Tel. ______________

Unfalldatum ______________
Unfalldiagnose
OP Datum ______________
Operative Versorung ______________
Therapiegruppe______________
Einwilligung ☐ ja ☐nein
Einwilligungsdatum ______________

Risikoprofil

- ☐ Abnahme der Körpergröße > 4cm
- ☐ Alkoholabusus
- ☐ Alter > 70
- ☐ Anorexia nervosa
- ☐ Antiepileptikatherapie
- ☐ Bereits osteoporotische Frakturen
- ☐ BMI < 20 kg/m^2
- ☐ Chronische Niereninsuffizienz/ Leberschaden
- ☐ Familiäre Belastung
- ☐ Frühe Menopause (< 45. Lj.) Späte Menarche (>15. Lj.)
- ☐ Hochdosis Heparintherapie
- ☐ Hormontherapie
- ☐ Hyperparathyroidismus
- ☐ Hyperthyreose
- ☐ (NI)IDDM
- ☐ Immobilisation; Inaktivität
- ☐ Maladsorptionssyndrom
- ☐ Multiple Sklerose
- ☐ Nicotinabusus
- ☐ Rheumatoide Arthritis

Ausschlusskriterien

- ☐ Polytrauma
- ☐ Erheblicher Weichteilschaden
- ☐ Hochgradig offene Fraktur
- ☐ Postop. > 24h Beatmungspflicht
- ☐ Dialysepflicht
- ☐ Dauertherapie mit Immunsuppressiva
- ☐ Kollagenose
- ☐ CED
- ☐ Hämatologische Erkrankungen
- ☐ Malignome

Einverständniserklärung

Osteoporotische Frakturen, Korrelation zwischen den humanen Wachstumsfaktoren und Östradiol, Vitamin D und Procalcitonin im Serum von Patienten mit Altersfrakturen ohne adäquatem Trauma.

(An der Studie teilnehmender Patienten)

Ludwigshafen, den ________

Name: ________________________________

Geburtsdatum: ________________

Die schriftliche Patienten/Probanden-Aufklärung habe ich erhalten und gelesen. Darüber hinaus bin ich mündlich aufgeklärt worden. Dabei wurden alle meine Fragen beantwortet.

Ich stimme der Teilnahme an der Studie freiwillig zu. Ich weiß, dass ich diese Zustimmung ohne Angabe von Gründen jederzeit und ohne Nachteile für meine weitere medizinische Versorgung widerrufen kann.

Bei Rücktritt von der Studie bin ich mit der Auswertung meines (Daten-) Materials einverstanden

Oja Onein

Ich wurde darüber aufgeklärt und stimme zu, dass die im Rahmen dieser Studie erhobenen Daten in anonymisierter Form dokumentiert und ggf. weitergegeben werden.
Dritte erhalten keinen Einblick in Originalkrankenunterlagen.

Ich erkläre mich mit der Entnahme von

Bluterguss aus dem Knochenbruchbereich	einverstanden	________________
Venenblutproben	einverstanden	________________
Entnahme von Knochenstückchen	einverstanden	________________

Ich erkläre mich mit der Aufbewahrung der Proben über den Studienzeitraum hinaus einverstanden ________________

Ärztin/Arzt ________________________________

Fragebogen zur Erstellung eines Patientenprofils (ID________________)
Osteoporotische Frakturen, Korrelation zwischen den humanen Wachstumsfaktoren und Östradiol, Vitamin D und Procalcitonin im Serum von Patienten mit Altersfrakturen ohne adäquatem Trauma.

Name	___________	Unfalldatum	___________
Vorname	___________	Unfalldiagnose	
Geb.	___________	OP Datum	___________
Geschlecht	___________	Operative Versorung	___________
Adresse	___________	Therapiegruppe	___________
Ort	___________	Einwilligung	☐ ja ☐nein
Tel.	___________	Einwilligungsdatum	___________

Risikoprofil

- ☐ Abnahme der Körpergröße > 4cm
- ☐ Alkoholabusus
- ☐ Alter > 70
- ☐ Anorexia nervosa
- ☐ Antiepileptikatherapie
- ☐ Bereits osteoporotische Frakturen
- ☐ BMI < 20 kg/m^2
- ☐ Chronische Niereninsuffizienz/ Leberschaden
- ☐ Familiäre Belastung
- ☐ Frühe Menopause (< 45. Lj.) Späte Menarche (>15. Lj.)
- ☐ Hochdosis Heparintherapie
- ☐ Hormontherapie
- ☐ Hyperparathyroidismus
- ☐ Hyperthyreose
- ☐ (NI)IDDM
- ☐ Immobilisation; Inaktivität
- ☐ Maladsorptionssyndrom
- ☐ Multiple Sklerose
- ☐ Nicotinabusus
- ☐ Rheumatoide Arthritis

Ausschlusskriterien

- ☐ Polytrauma
- ☐ Erheblicher Weichteilschaden
- ☐ Hochgradig offene Fraktur
- ☐ Postop. > 24h Beatmungspflicht
- ☐ Dialysepflicht
- ☐ Dauertherapie mit Immunsuppressiva
- ☐ Kollagenose
- ☐ CED
- ☐ Hämatologische Erkrankungen
- ☐ Malignome
