# Supplementary material for: Correction: Characteristics of bone turnover in the long bone metaphysis fractured patients with normal or low Bone Mineral Density (BMD)
Source: PLoS One. 2022 Jun 13;17(6):e0270079. doi: 10.1371/journal.pone.0270079 (PMC9191733; doi:10.1371/journal.pone.0270079)

Tests auf Normalverteilung

	Osteoporose	Kolmogorov-Smirnov Statistik	df	Signifikanz	Shapiro-Wilk Statistik	Df	Signifikanz
Bap1	N-Osteo	,143	15	,200	,941	15	,398
	Osteo	,156	11	,200	,947	11	,611
Bap2	N-Osteo	,143	15	,200	,958	15	,652
	Osteo	,149	11	,200	,940	11	,524
Bap3	N-Osteo	,164	15	,200	,941	15	,392
	Osteo	,189	11	,200	,944	11	,574
Bap4	N-Osteo	,186	15	,171	,946	15	,463
	Osteo	,184	11	,200	,923	11	,347
TRA1	N-Osteo	,127	15	,200	,951	15	,542
	Osteo	,135	11	,200	,945	11	,584
TRA2	N-Osteo	,247	15	,014	,897	15	,084
	Osteo	,159	11	,200	,961	11	,785
TRA3	N-Osteo	,110	15	,200	,969	15	,850
	Osteo	,113	11	,200	,967	11	,853
TRA4	N-Osteo	,176	15	,200	,861	15	,025
	Osteo	,140	11	,200	,954	11	,693
CTX1	N-Osteo	,156	15	,200	,954	15	,585
	Osteo	,146	11	,200	,936	11	,472
CTX2	N-Osteo	,162	15	,200	,959	15	,675
	Osteo	,269	11	,025	,900	11	,186
CTX3	N-Osteo	,118	15	,200	,933	15	,304
	Osteo	,317	11	,003	,789	11	,007
CTX4	N-Osteo	,174	15	,200	,912	15	,146
	Osteo	,170	11	,200	,931	11	,418
TGF1	N-Osteo	,136	15	,200	,951	15	,535
	Osteo	,163	11	,200	,942	11	,545
TGF2	N-Osteo	,166	15	,200	,944	15	,439
	Osteo	,210	11	,189	,916	11	,286
TGF3	N-Osteo	,185	15	,179	,919	15	,186
	Osteo	,238	11	,083	,861	11	,059
TGF4	N-Osteo	,131	15	,200	,947	15	,482
	Osteo	,132	11	,200	,947	11	,604

Abbreviations:

n-Osteo **high BMD**
Osteo **low BMD**

Bap1
Stengel-Blatt-Diagramme

Bap1 Stamm-Blatt-Diagramm für Osteoporose= N-Osteo	Bap1 Stamm-Blatt-Diagramm für Osteoporose= Osteo
<pre>Häufigkeit Stem & Blatt<br>2,00 0 . 12<br>1,00 0 . 8<br>4,00 1 . 0234<br>2,00 1 . 79<br>4,00 2 . 0444<br>2,00 2 . 77<br><br>Stammbreite: 10,00<br>Jedes Blatt: 1 Fälle</pre>	<pre>Häufigkeit Stem & Blatt<br>1,00 0 . 4<br>3,00 0 . 889<br>5,00 1 . 00134<br>1,00 1 . 5<br>1,00 Extremwerte (>=22)<br><br>Stammbreite: 10,00<br>Jedes Blatt: 1 Fälle</pre>

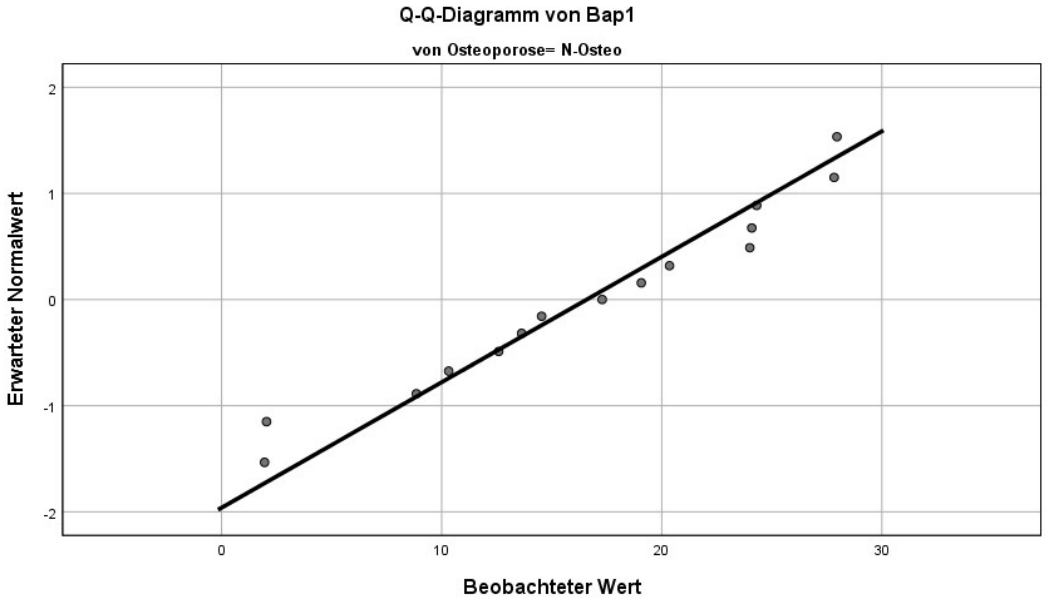

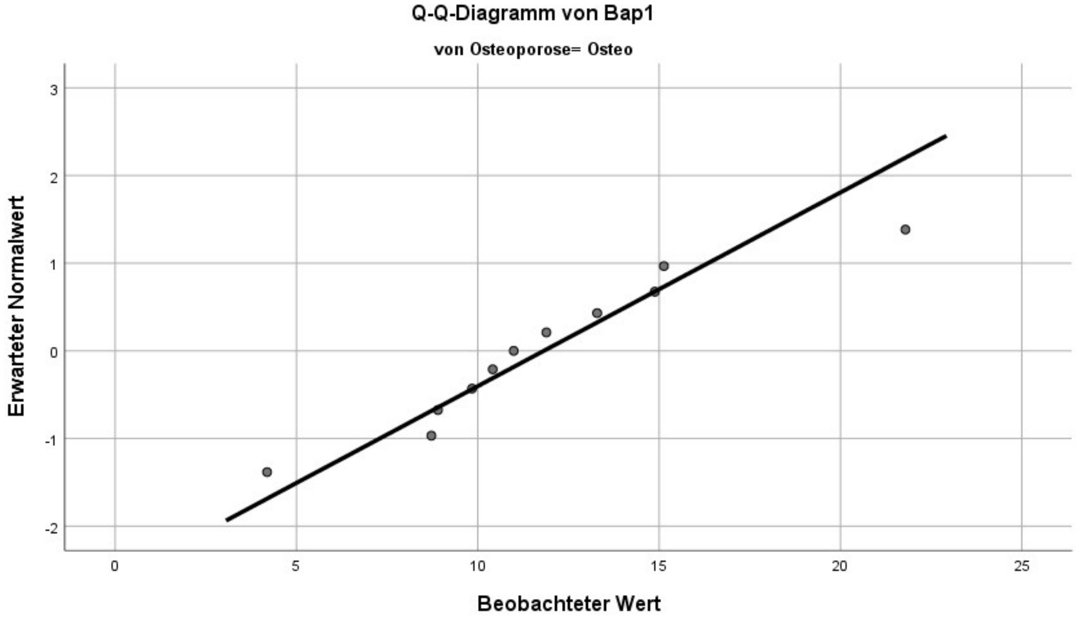

Trendbereinigte normalverteilte Q-Q-Diagramme

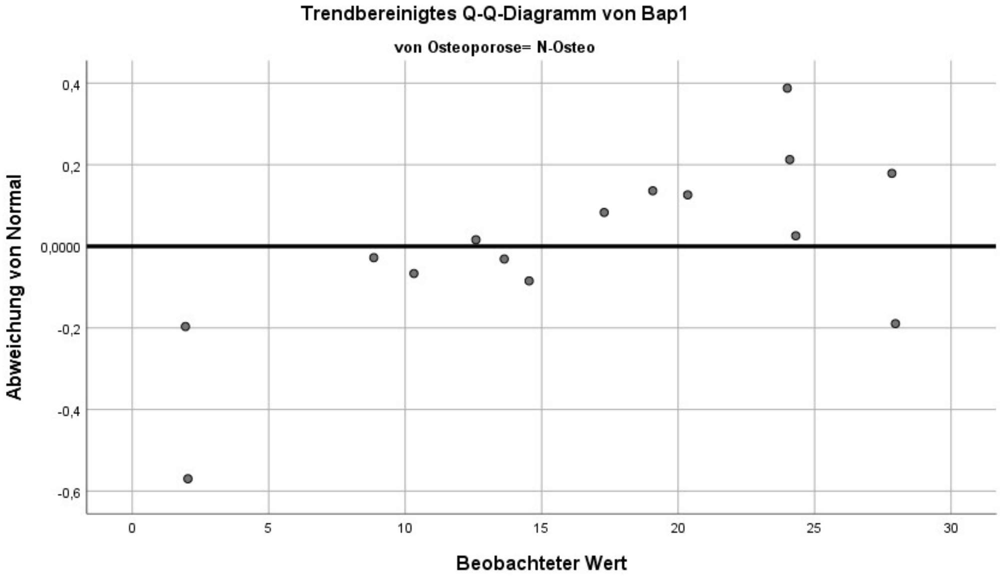

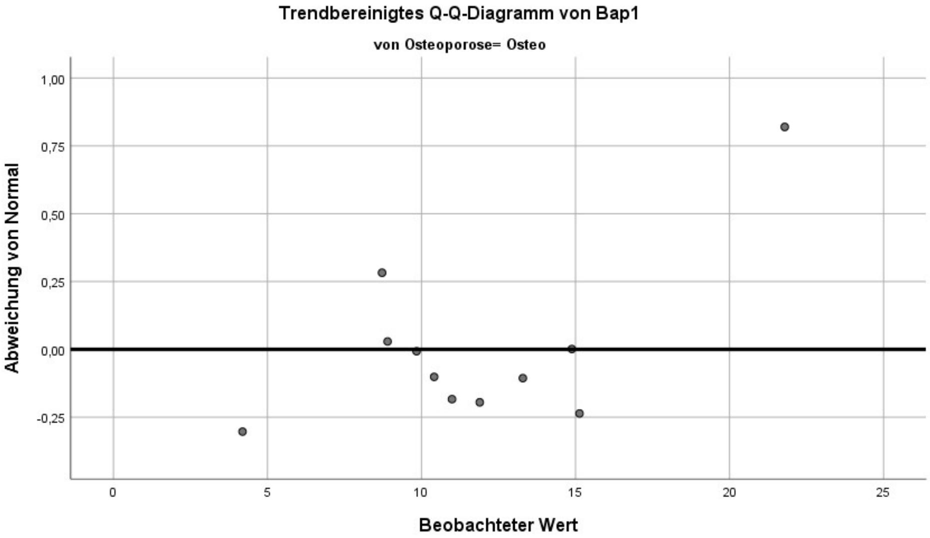

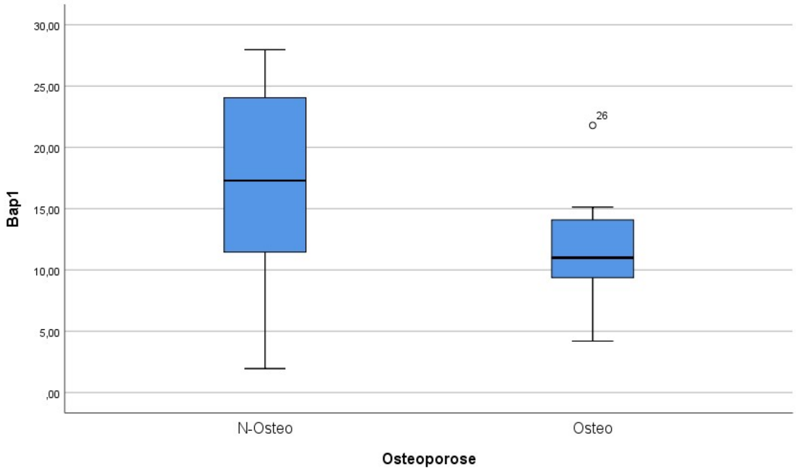

Bap2
Stengel-Blatt-Diagramme

```
Bap2 Stamm-Blatt-Diagramm für
Osteoporose= N-Osteo

 Häufigkeit  Stem &  Blatt

     1,00 Extremes    (=<1)
     1,00        0 .  4
    11,00        0 .  66677888999
     1,00        1 .  1
     1,00 Extremwerte (>=14)

 Stammbreite:     10,00
 Jedes Blatt:        1 Fälle
```

```
Bap2 Stamm-Blatt-Diagramm für
Osteoporose= Osteo

 Häufigkeit  Stem &  Blatt

     2,00        0 .  99
     6,00        1 .  002344
     2,00        1 .  67
     1,00        2 .  1

 Stammbreite:     10,00
 Jedes Blatt:        1 Fälle
```

Normalverteilote Q-Q-Diagramme

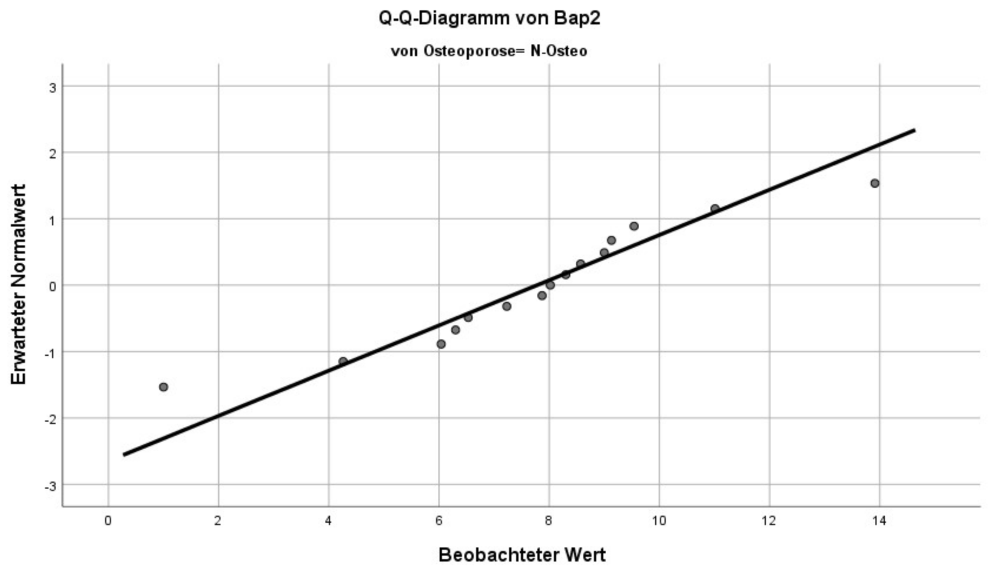

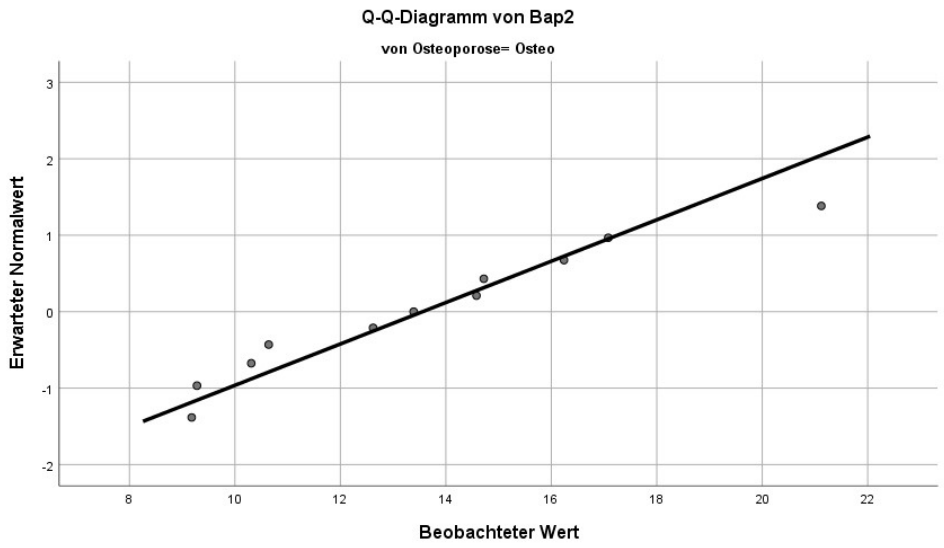

Trendbereinigte normalverteilte Q-Q-Diagramme

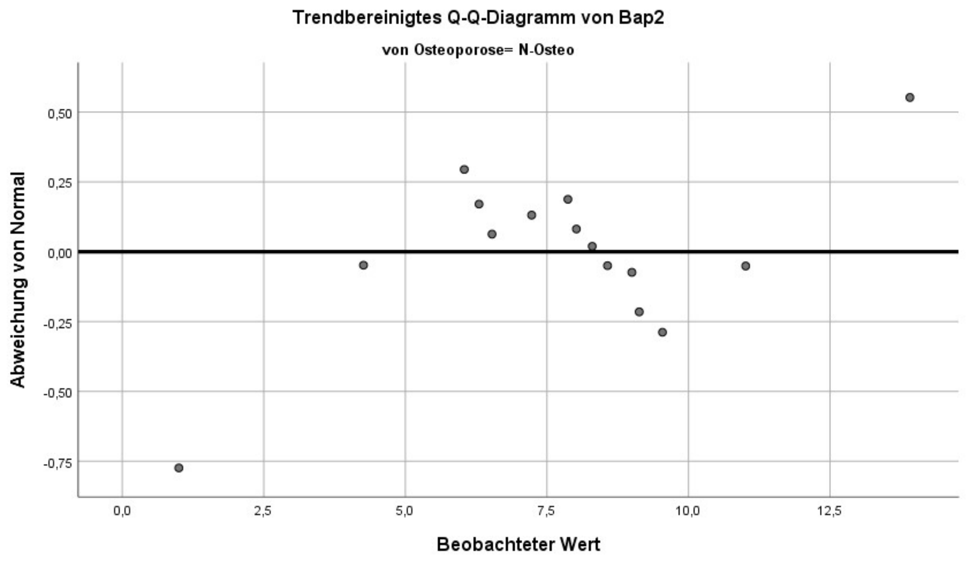

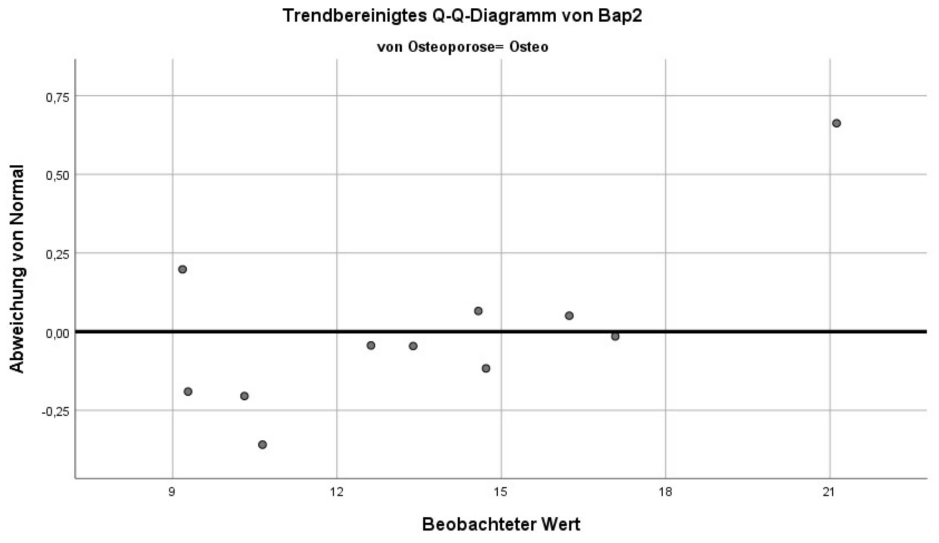

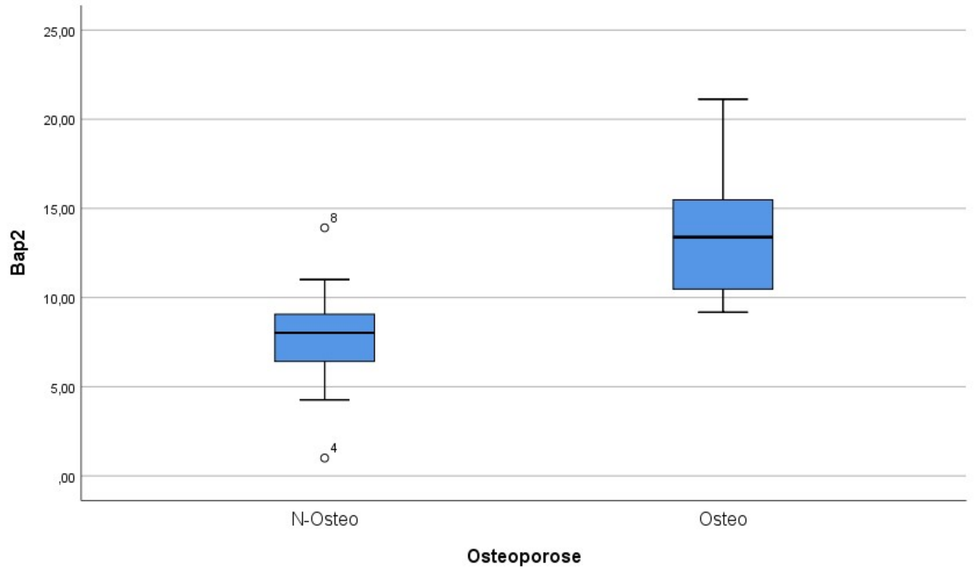

Bap3
Stengel-Blatt-Diagramme

```
Bap3 Stamm-Blatt-Diagramm für
Osteoporose= N-Osteo

 Häufigkeit  Stem &  Blatt

     2,00        0 .  14
     6,00        0 .  677789
     5,00        1 .  00011
     1,00        1 .  5
     1,00 Extremwerte (>=21)

 Stammbreite:     10,00
 Jedes Blatt:        1 Fälle
```

```
Bap3 Stamm-Blatt-Diagramm für
Osteoporose= Osteo

 Häufigkeit  Stem &  Blatt

     1,00 Extremes    (=<8)
     4,00        1 .  3344
     4,00        1 .  5568
     1,00        2 .  1
     1,00 Extremwerte (>=25)

 Stammbreite:     10,00
 Jedes Blatt:        1 Fälle
```

Normalverteilote Q-Q-Diagramme

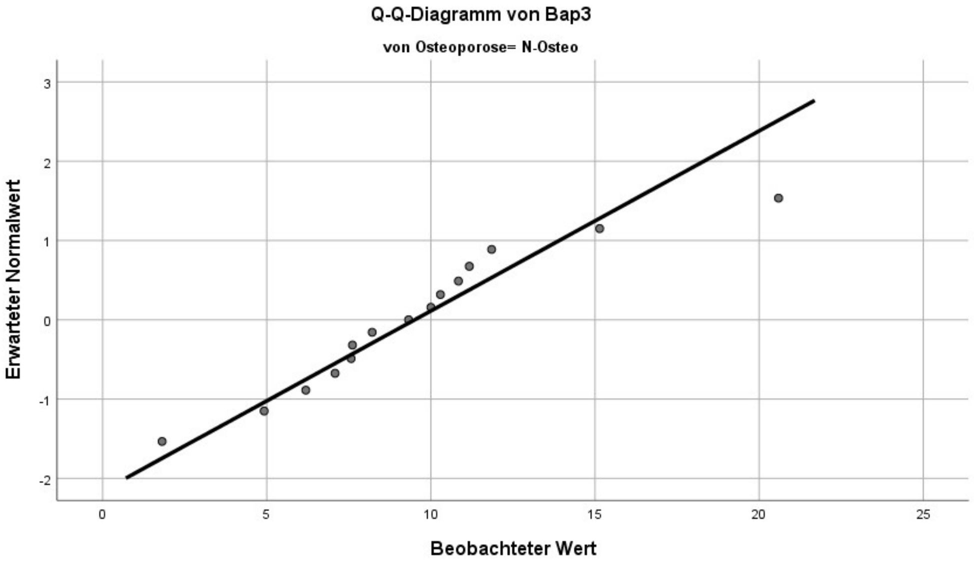

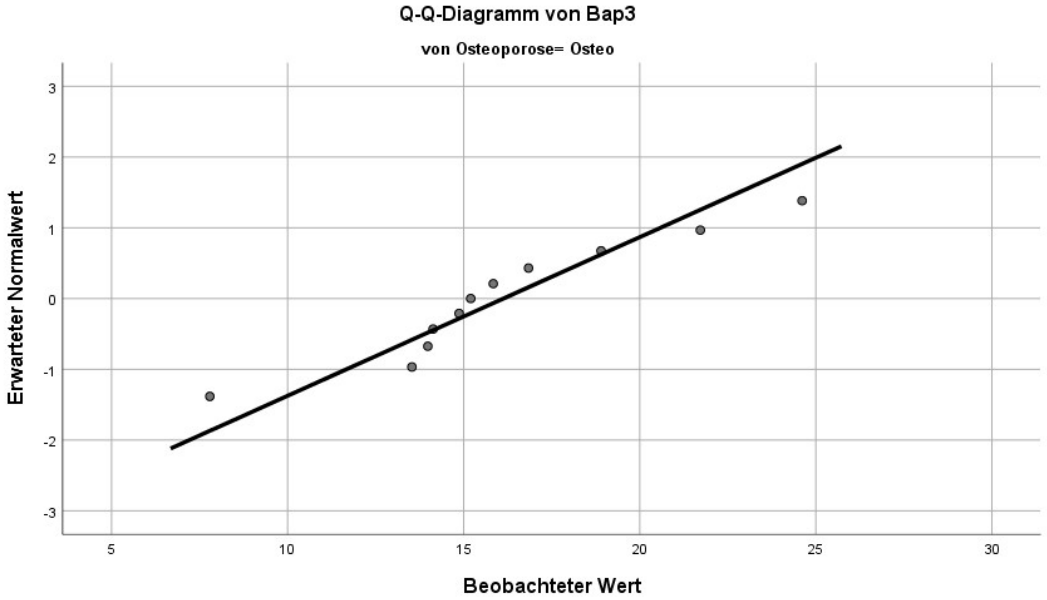

Trendbereinigte normalverteilte Q-Q-Diagramme

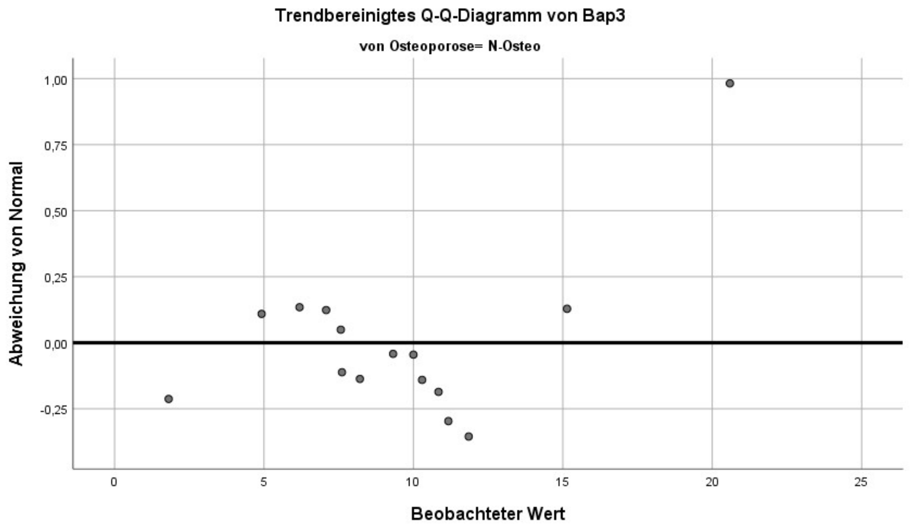

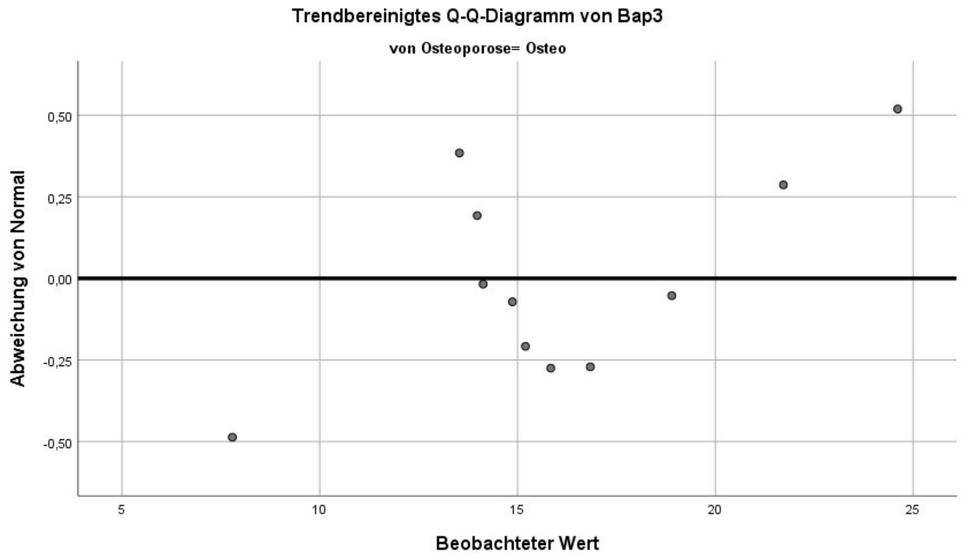

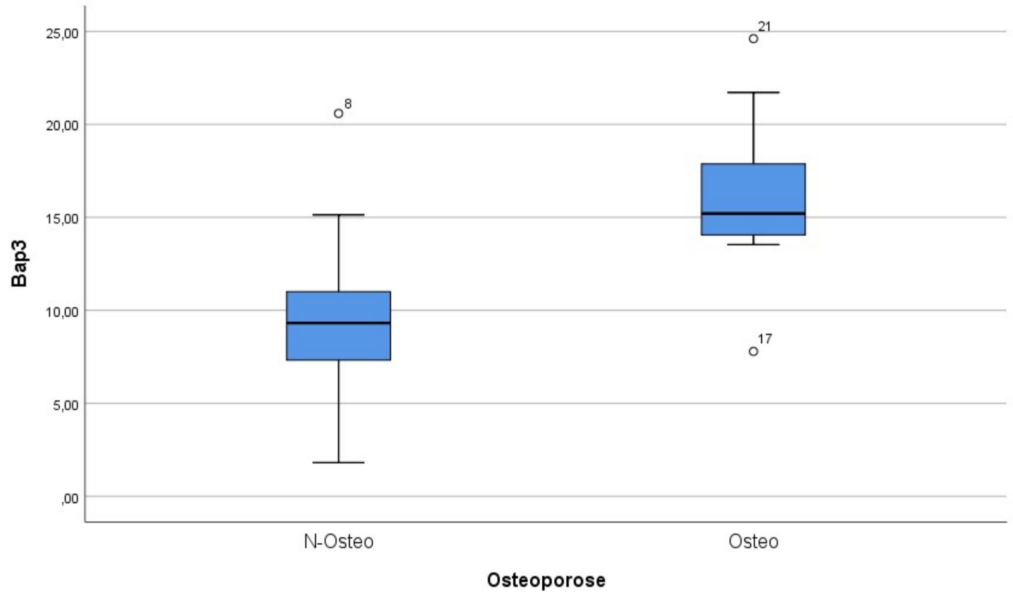

Bap4
Stengel-Blatt-Diagramme

```
Bap4 Stamm-Blatt-Diagramm für
Osteoporose= N-Osteo

 Häufigkeit  Stem &  Blatt

     1,00        0 .  3
     3,00        0 .  689
     7,00        1 .  0022224
     2,00        1 .  59
     1,00        2 .  0
     1,00 Extremwerte (>=27)

 Stammbreite:     10,00
 Jedes Blatt:         1 Fälle
```

```
Bap4 Stamm-Blatt-Diagramm für
Osteoporose= Osteo

 Häufigkeit  Stem &  Blatt

     1,00 Extremes    (=<7,0)
     2,00       12 .  07
     3,00       13 .  234
      ,00       14 .
     3,00       15 .  023
     1,00       16 .  3
     1,00 Extremwerte (>=19,9)

 Stammbreite:      1,00
 Jedes Blatt:         1 Fälle
```

Normalverteilote Q-Q-Diagramme

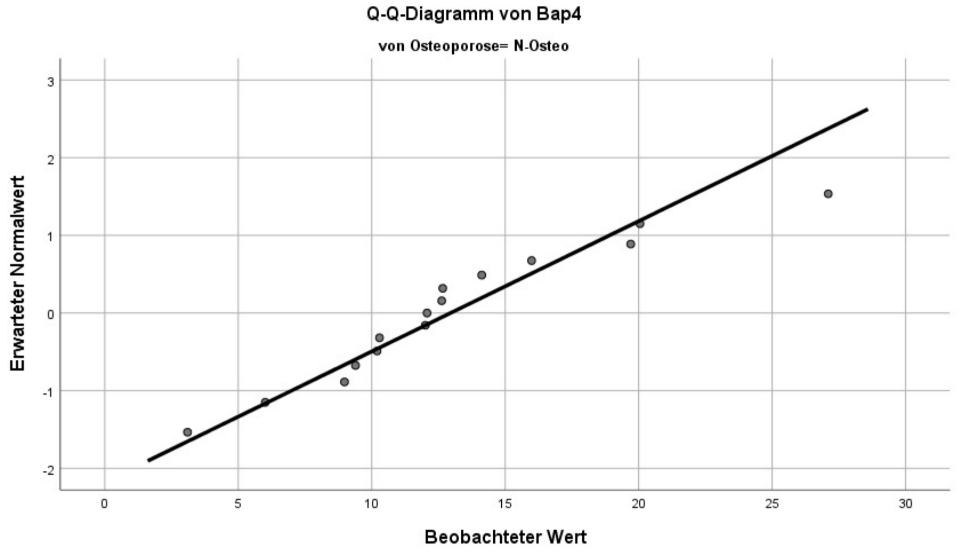

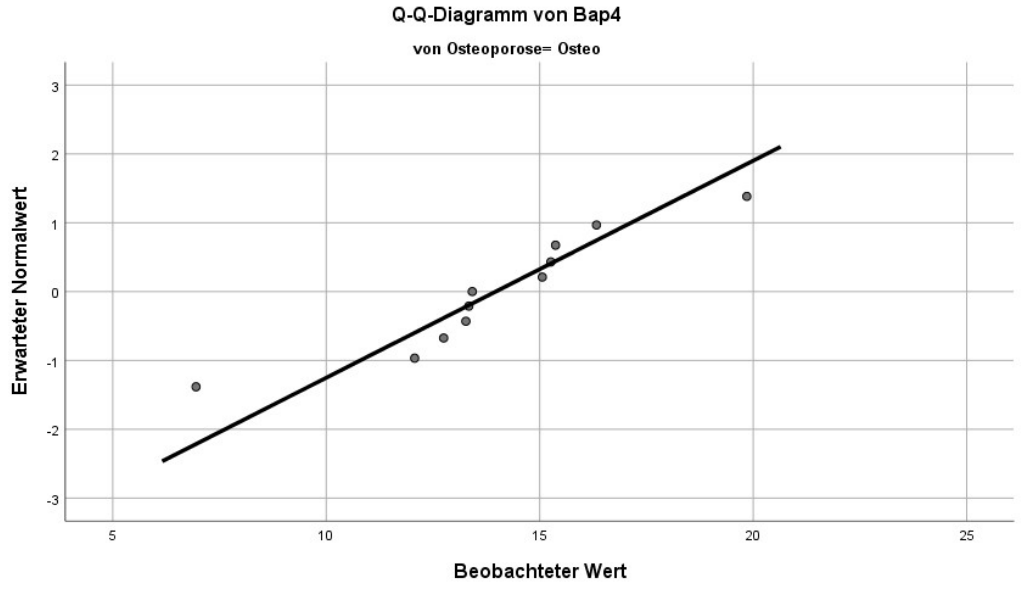

Trendbereinigte normalverteilte Q-Q-Diagramme

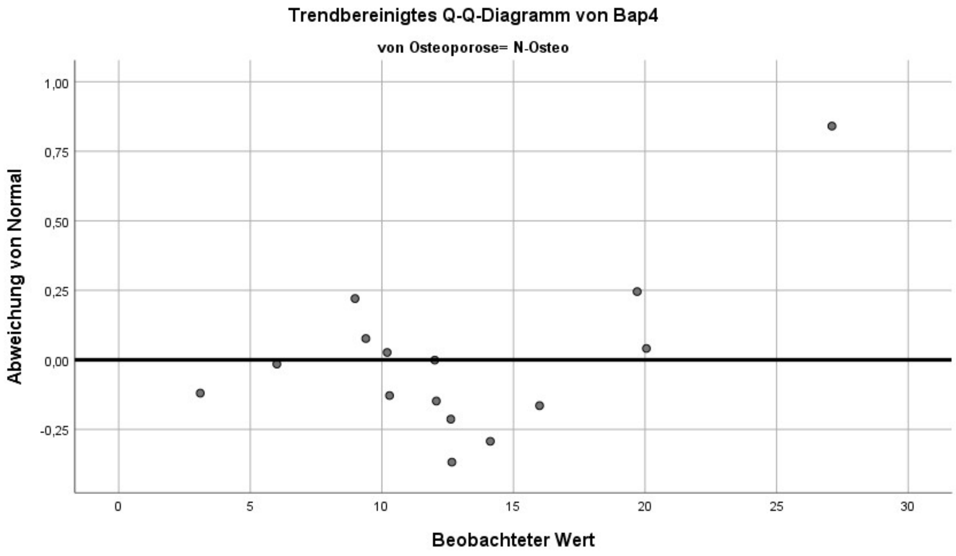

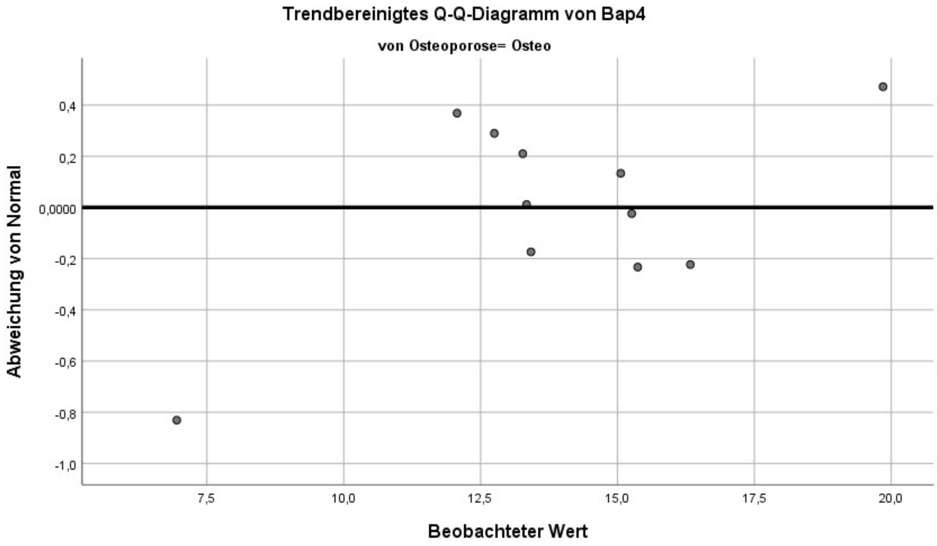

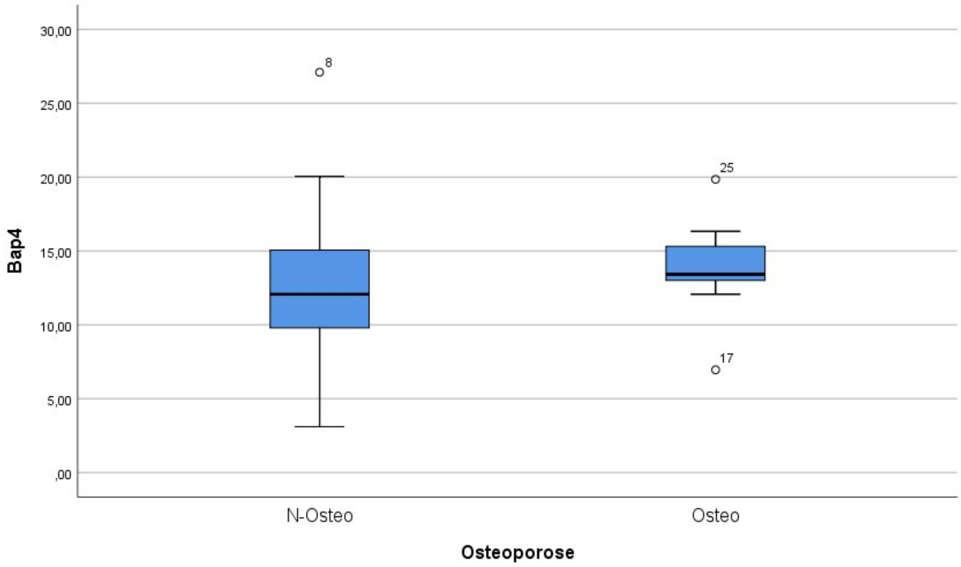

TRA1

Stengel-Blatt-Diagramme

TRA1 Stamm-Blatt-Diagramm für Osteoporose= N-Osteo	TRA1 Stamm-Blatt-Diagramm für Osteoporose= Osteo
Häufigkeit Stem & Blatt	Häufigkeit Stem & Blatt
4,00 4 . 0122	2,00 1 . 59
4,00 4 . 6778	3,00 2 . 347
5,00 5 . 01223	3,00 3 . 127
2,00 5 . 56	3,00 4 . 123
Stammbreite: 1,00	Stammbreite: 1,00
Jedes Blatt: 1 Fälle	Jedes Blatt: 1 Fälle

Normalverteilote Q-Q-Diagramme

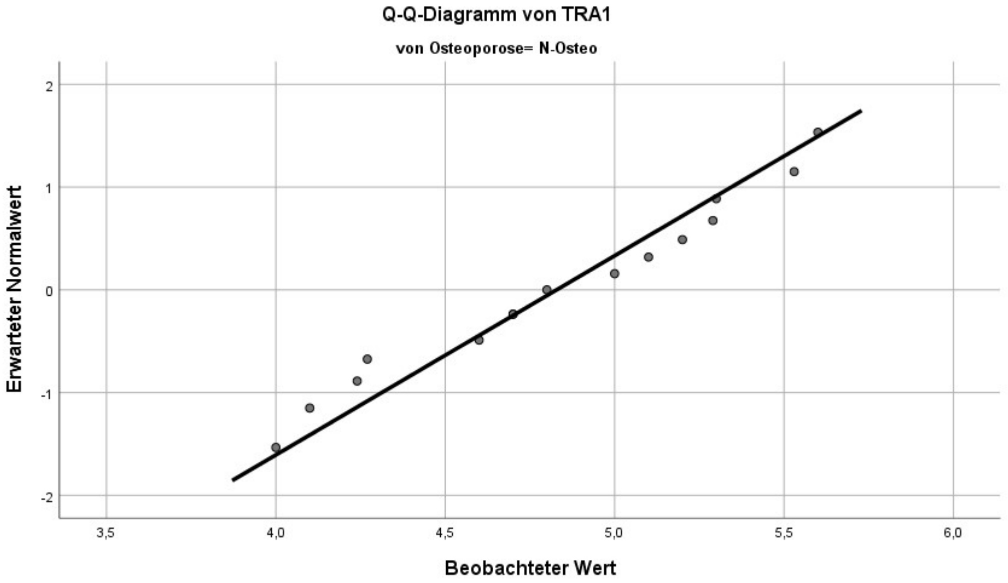

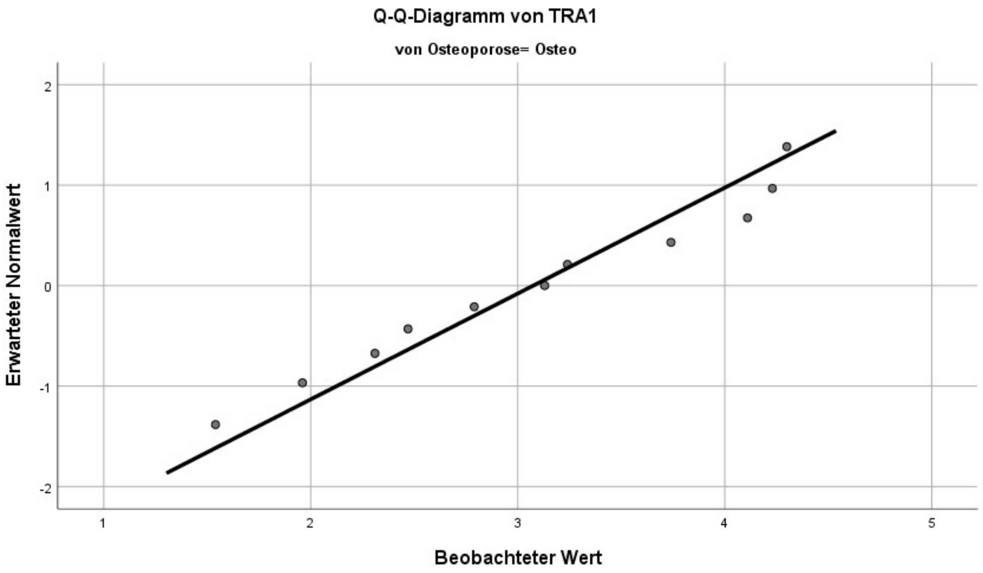

Trendbereinigte normalverteilte Q-Q-Diagramme

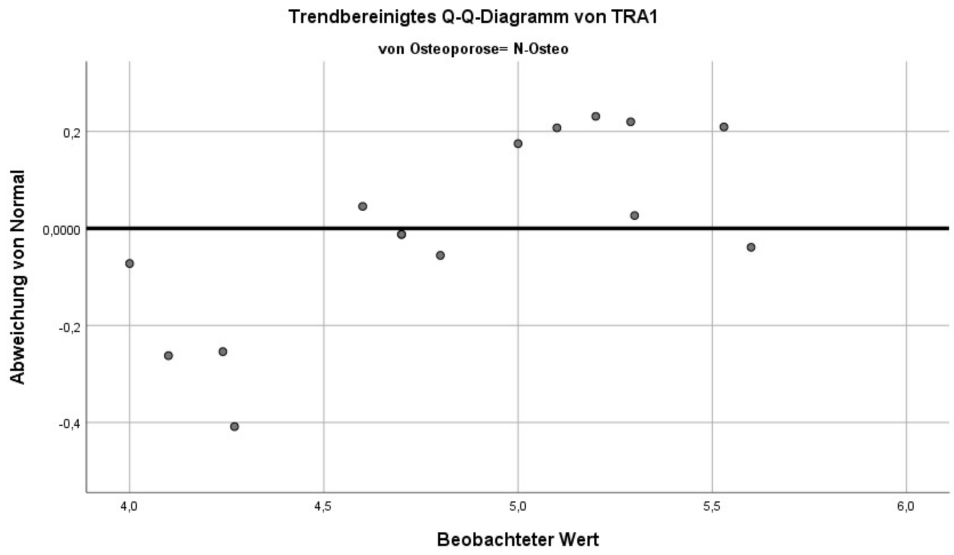

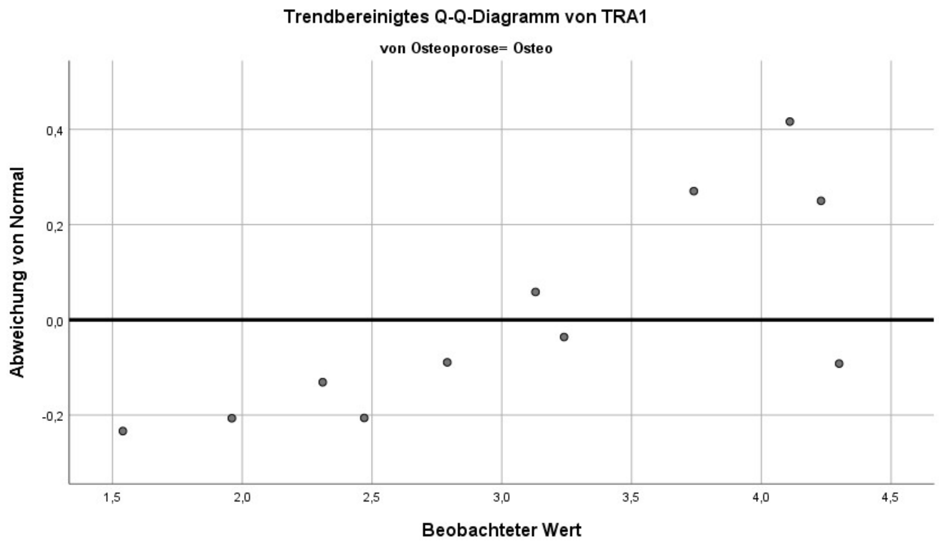

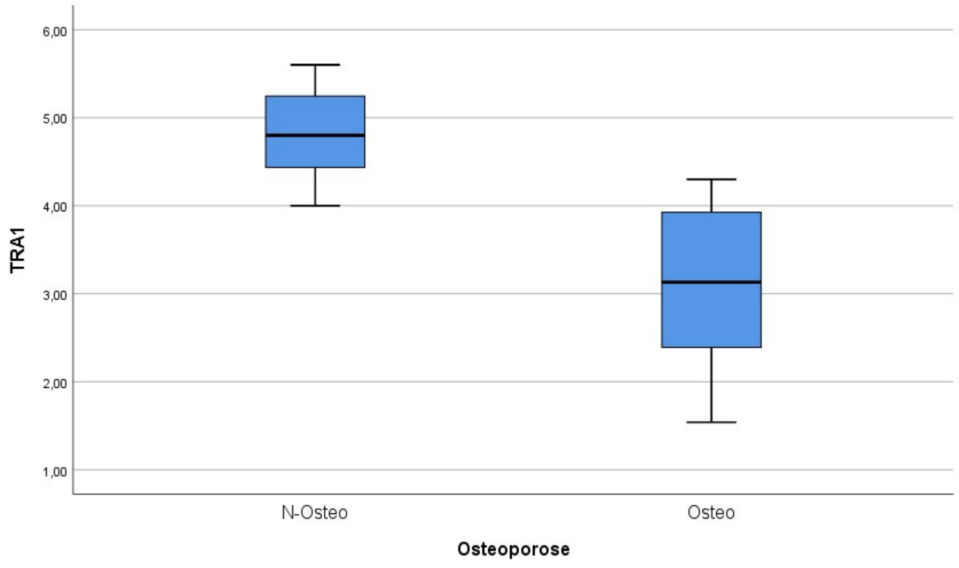

TRA2

Stengel-Blatt-Diagramme

TRA2 Stamm-Blatt-Diagramm für Osteoporose= N-Osteo	TRA2 Stamm-Blatt-Diagramm für Osteoporose= Osteo
Häufigkeit Stem & Blatt	Häufigkeit Stem & Blatt
,00 4 .	1,00 1 . 5
1,00 4 . 5	2,00 2 . 57
6,00 5 . 111122	5,00 3 . 22259
1,00 5 . 5	3,00 4 . 146
6,00 6 . 111112	Stammbreite: 1,00
1,00 6 . 6	Jedes Blatt: 1 Fälle
Stammbreite: 1,00	
Jedes Blatt: 1 Fälle	

Normalverteilote Q-Q-Diagramme

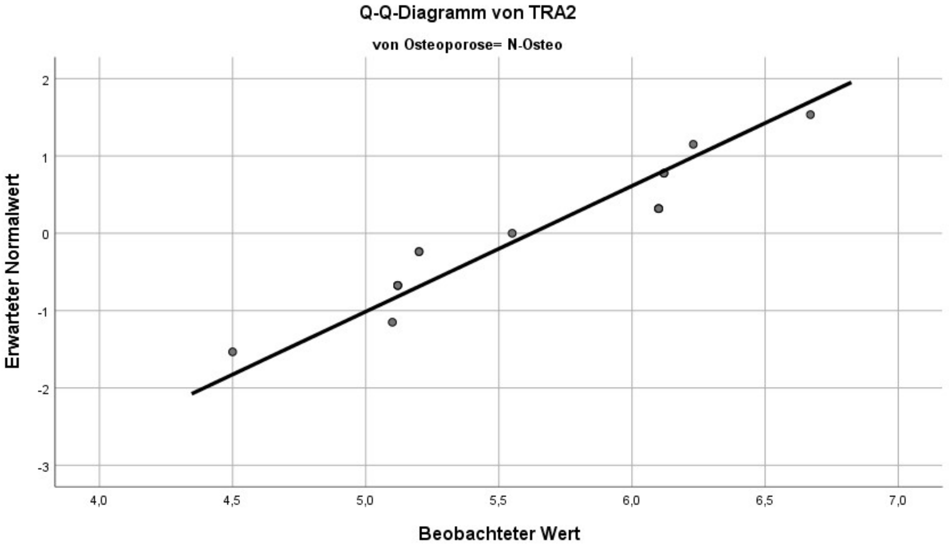

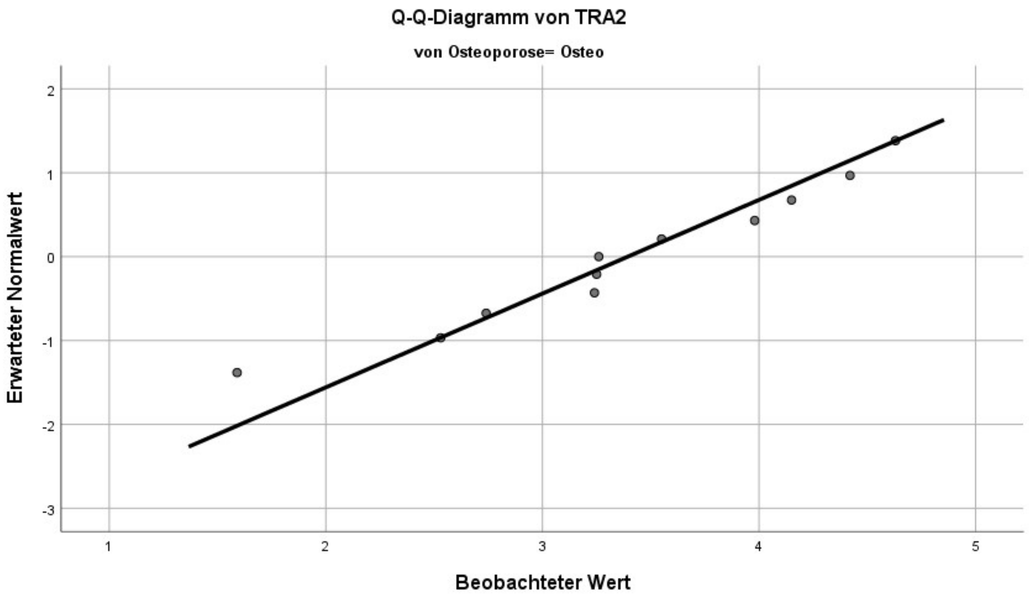

Trendbereinigte normalverteilte Q-Q-Diagramme

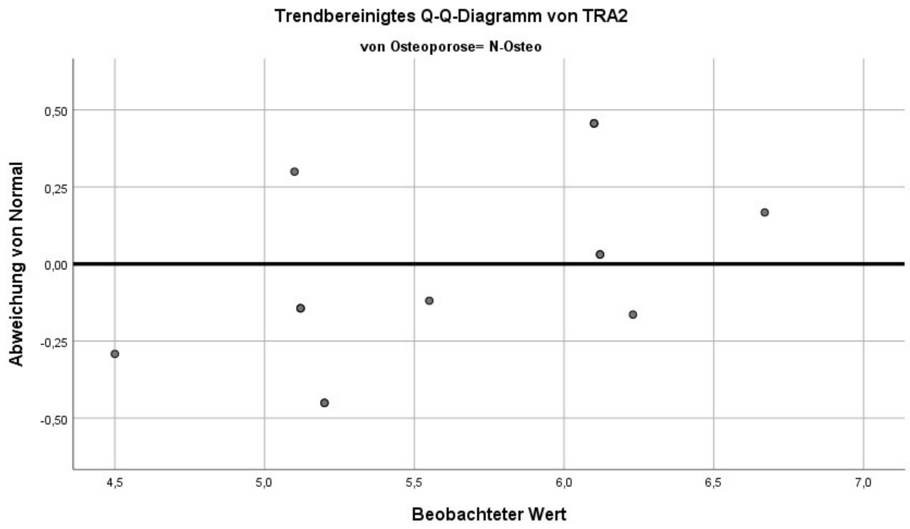

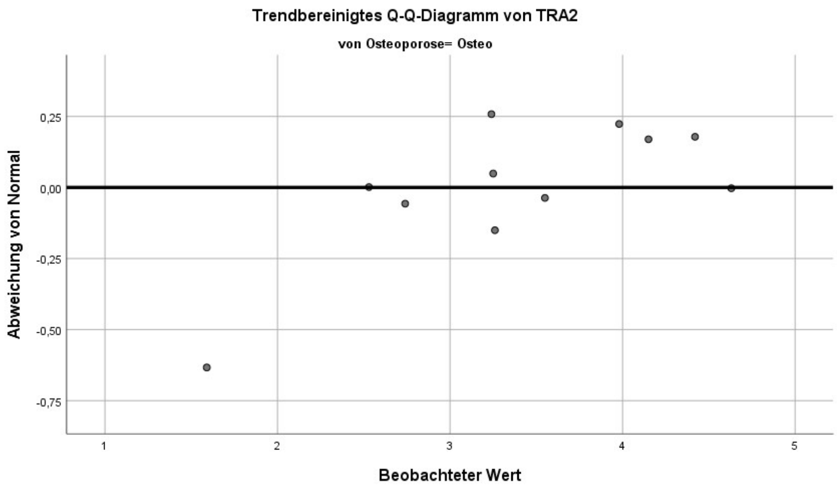

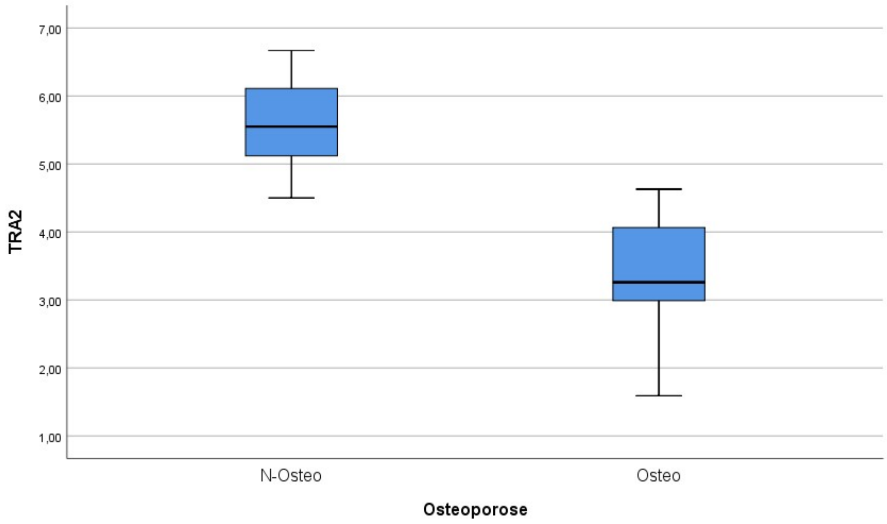

TRA3
Stengel-Blatt-Diagramme

```
TRA3 Stamm-Blatt-Diagramm für
Osteoporose= N-Osteo

 Häufigkeit  Stem &  Blatt

     1,00        4 .  0
      ,00        4 .
     4,00        5 .  0012
     5,00        5 .  56679
     4,00        6 .  0134
     1,00        6 .  7

 Stammbreite:      1,00
 Jedes Blatt:        1 Fälle
```

```
TRA3 Stamm-Blatt-Diagramm für
Osteoporose= Osteo

 Häufigkeit  Stem &  Blatt

     1,00        2 .  0
     2,00        2 .  79
     1,00        3 .  1
     2,00        3 .  56
     3,00        4 .  034
     2,00        4 .  99

 Stammbreite:      1,00
 Jedes Blatt:        1 Fälle
```

Normalverteilote Q-Q-Diagramme

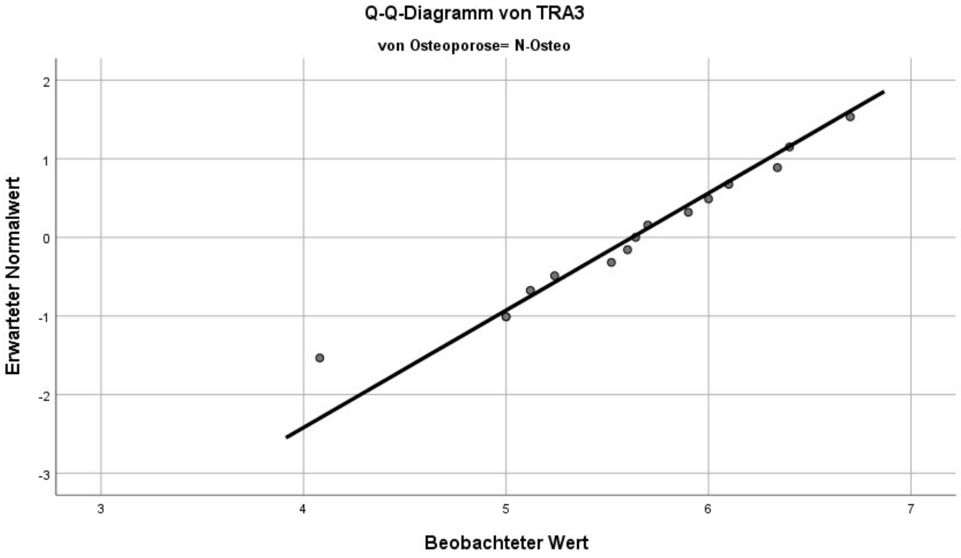

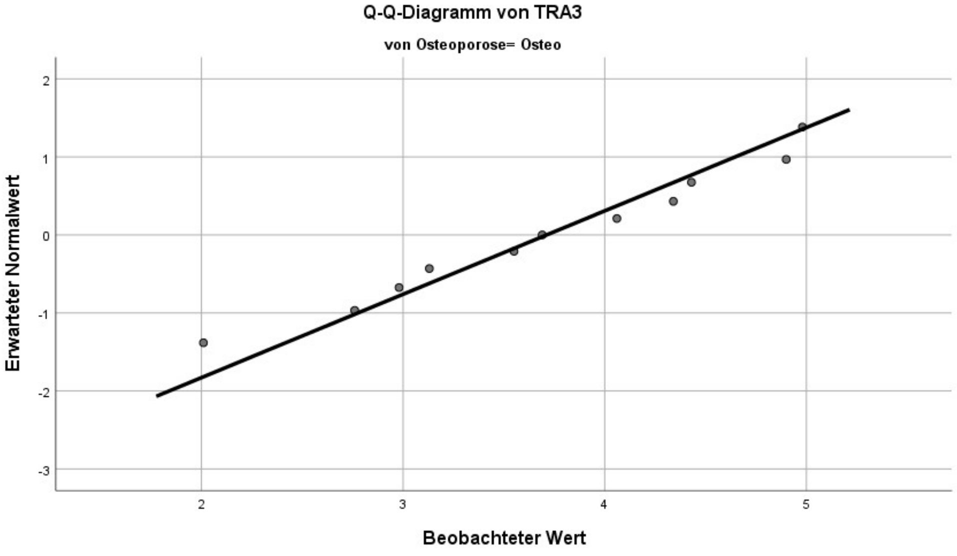

Trendbereinigte normalverteilte Q-Q-Diagramme

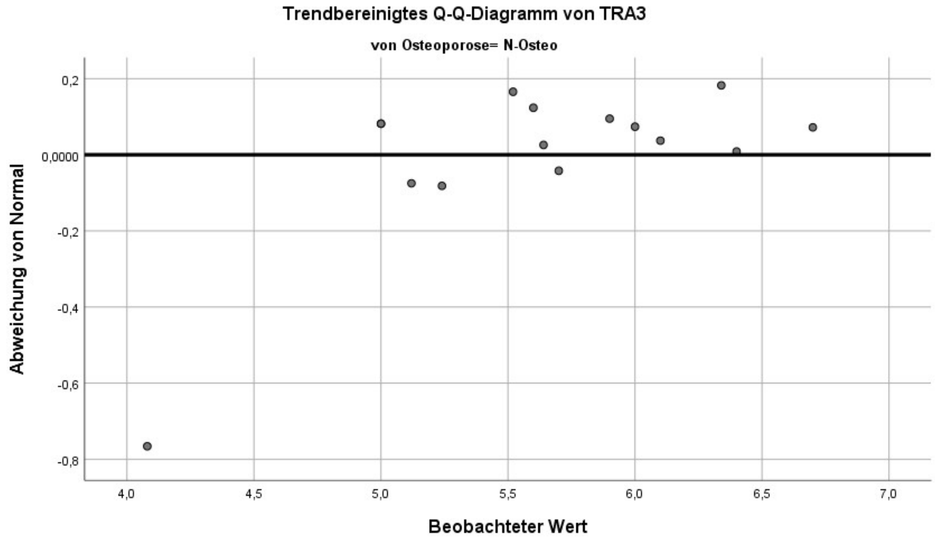

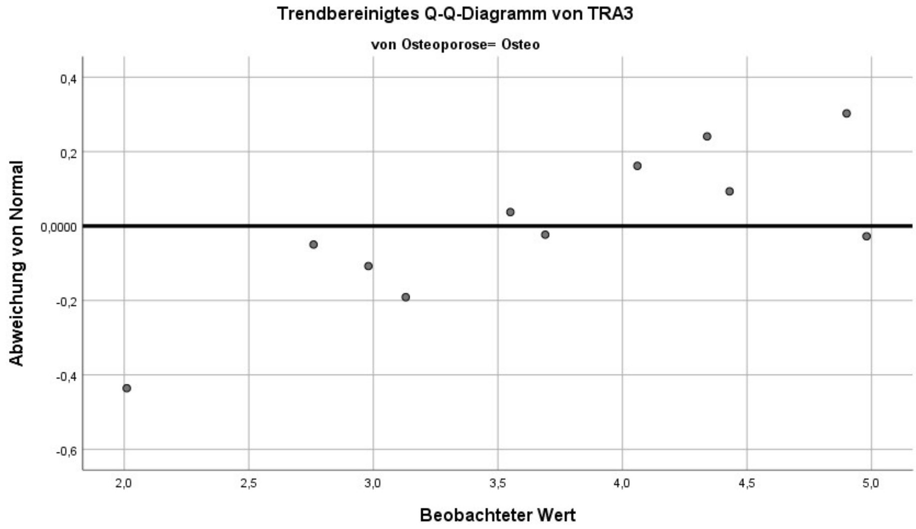

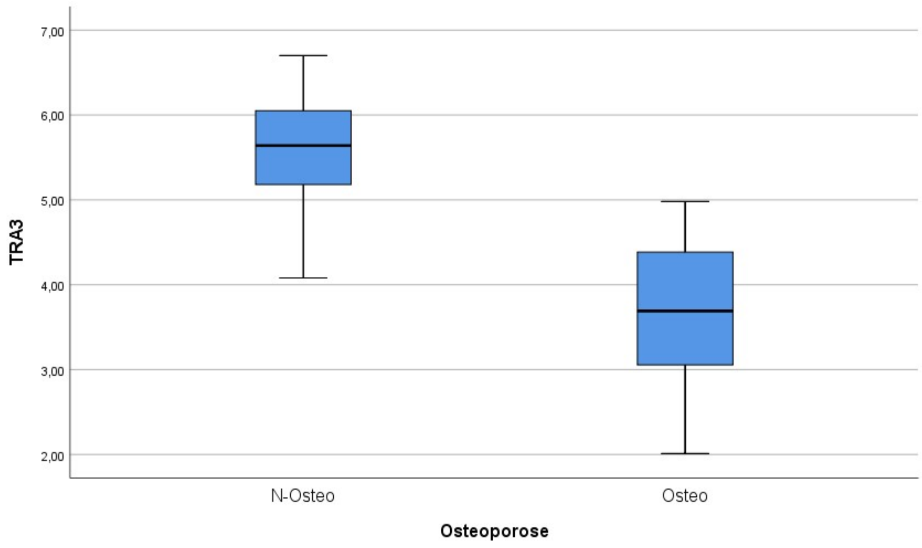

TRA4
Stengel-Blatt-Diagramme

TRA4 Stamm-Blatt-Diagramm für Osteoporose= N-Osteo	TRA4 Stamm-Blatt-Diagramm für Osteoporose= Osteo
Häufigkeit Stem & Blatt	Häufigkeit Stem & Blatt
1,00 Extremes (=<4,1)	4,00 2 . 0188
5,00 5 . 12333	3,00 3 . 257
3,00 5 . 578	3,00 4 . 089
6,00 6 . 000012	1,00 5 . 4
Stammbreite: 1,00	Stammbreite: 1,00
Jedes Blatt: 1 Fälle	Jedes Blatt: 1 Fälle

Normalverteilote Q-Q-Diagramme

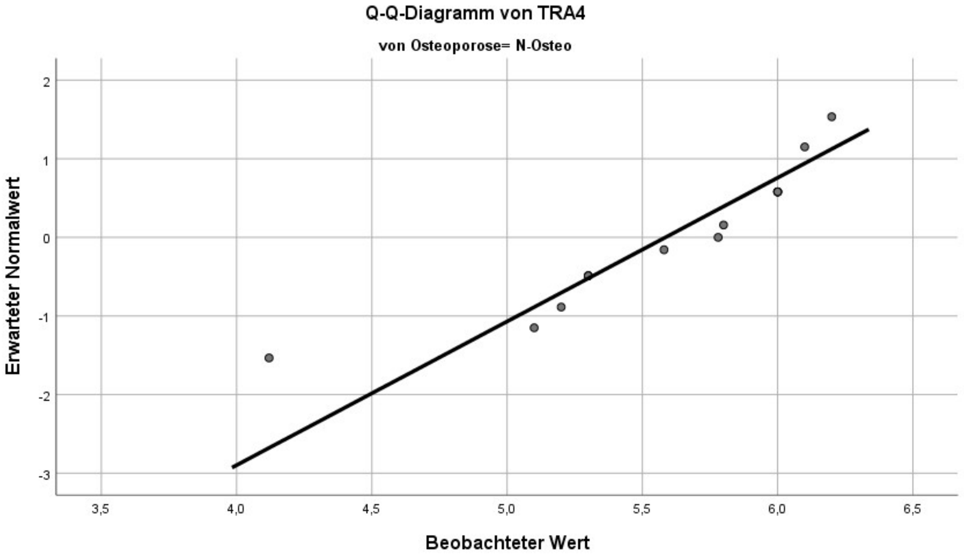

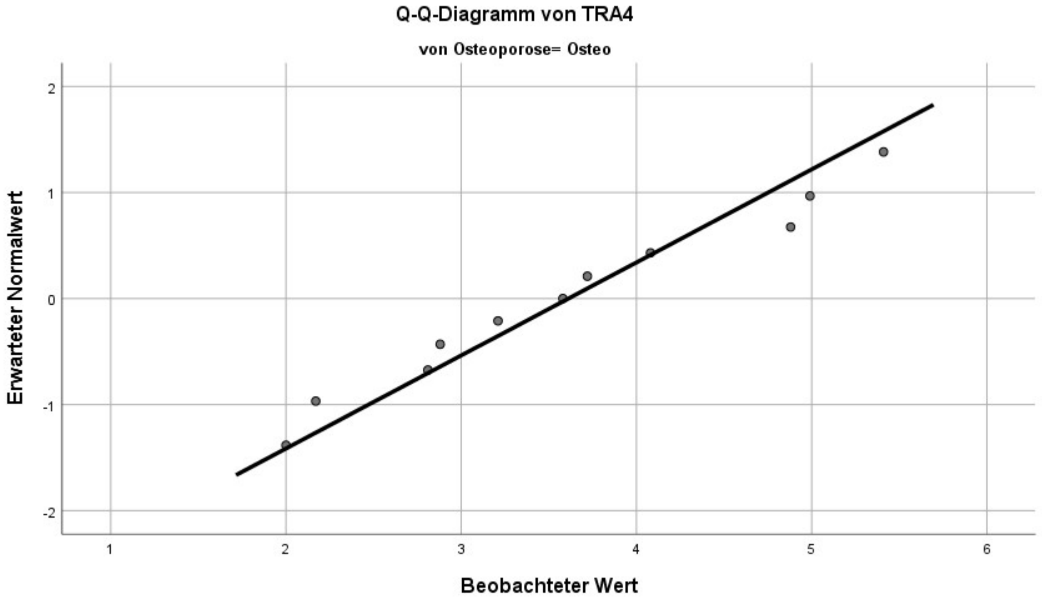

Trendbereinigte normalverteilte Q-Q-Diagramme

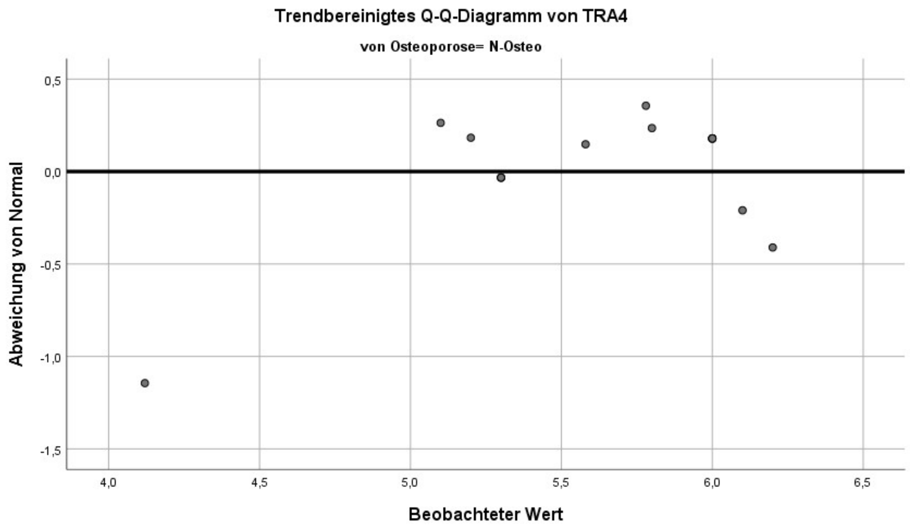

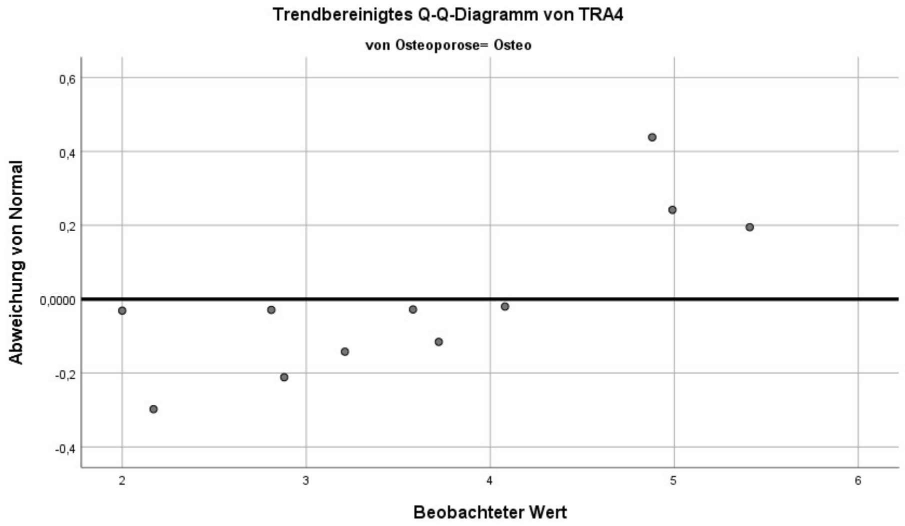

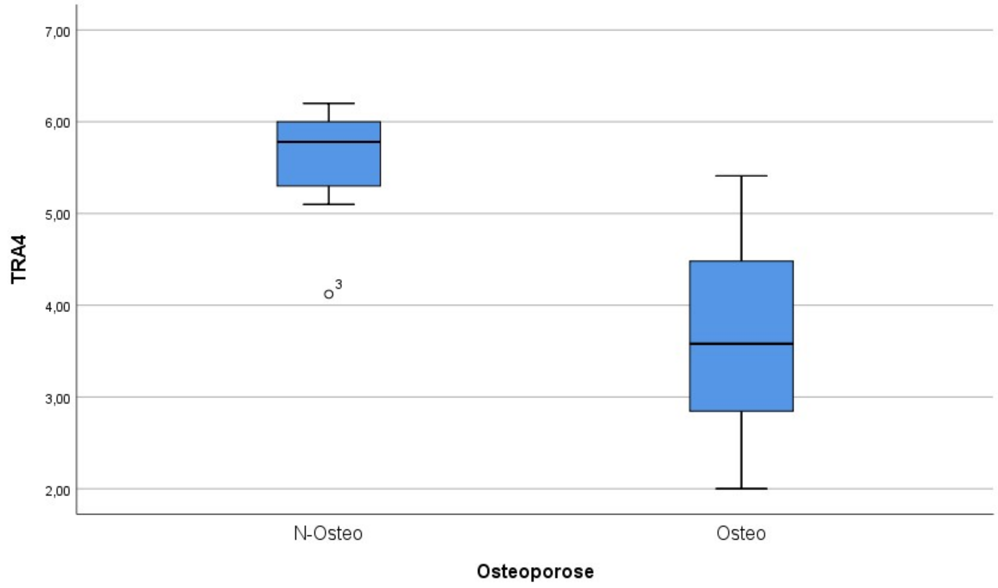

CTX1
Stengel-Blatt-Diagramme

CTX1 Stamm-Blatt-Diagramm für Osteoporose= N-Osteo	CTX1 Stamm-Blatt-Diagramm für Osteoporose= Osteo
Häufigkeit Stem & Blatt	Häufigkeit Stem & Blatt
2,00 1 . 47	1,00 2 . 5
2,00 2 . 78	3,00 3 . 259
1,00 3 . 3	2,00 4 . 78
4,00 4 . 4569	3,00 5 . 127
4,00 5 . 5679	1,00 6 . 1
1,00 6 . 6	1,00 Extremwerte (>=,87)
1,00 7 . 1	
Stammbreite: ,10	Stammbreite: ,10
Jedes Blatt: 1 Fälle	Jedes Blatt: 1 Fälle

Normalverteilote Q-Q-Diagramme

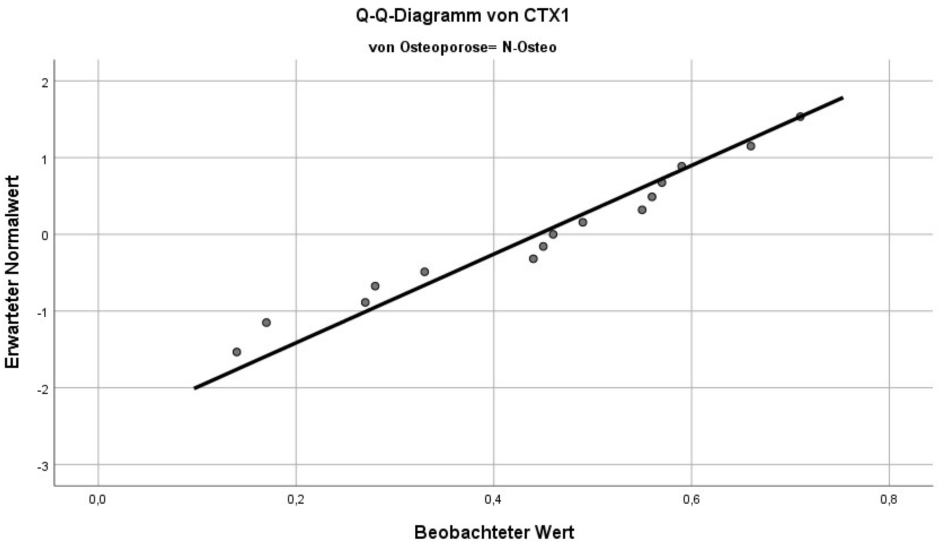

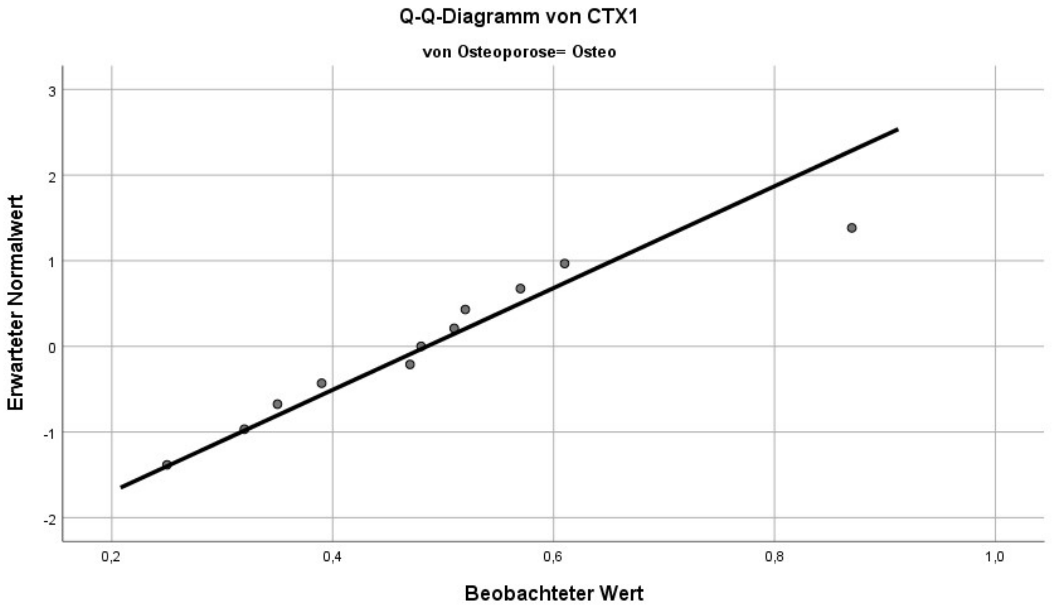

Trendbereinigte normalverteilte Q-Q-Diagramme

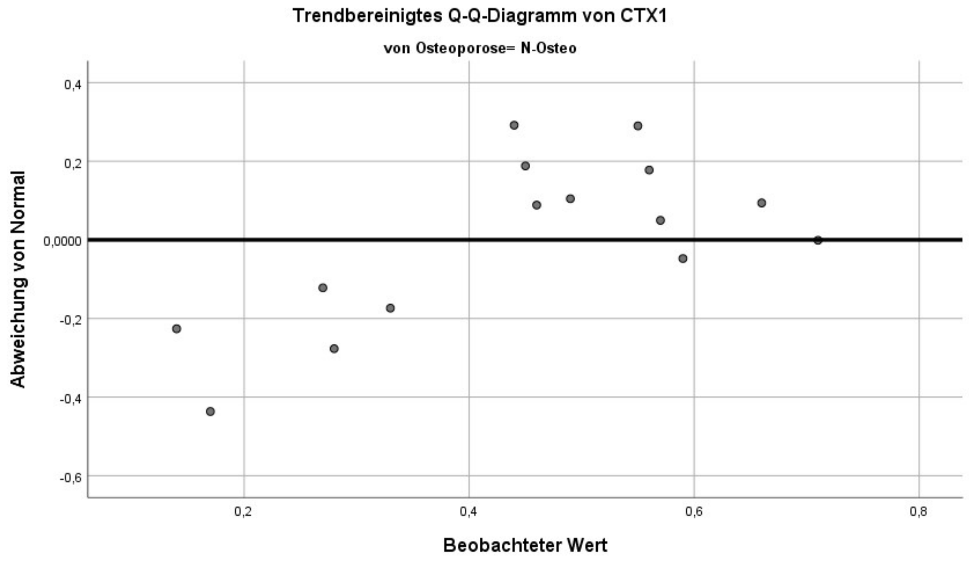

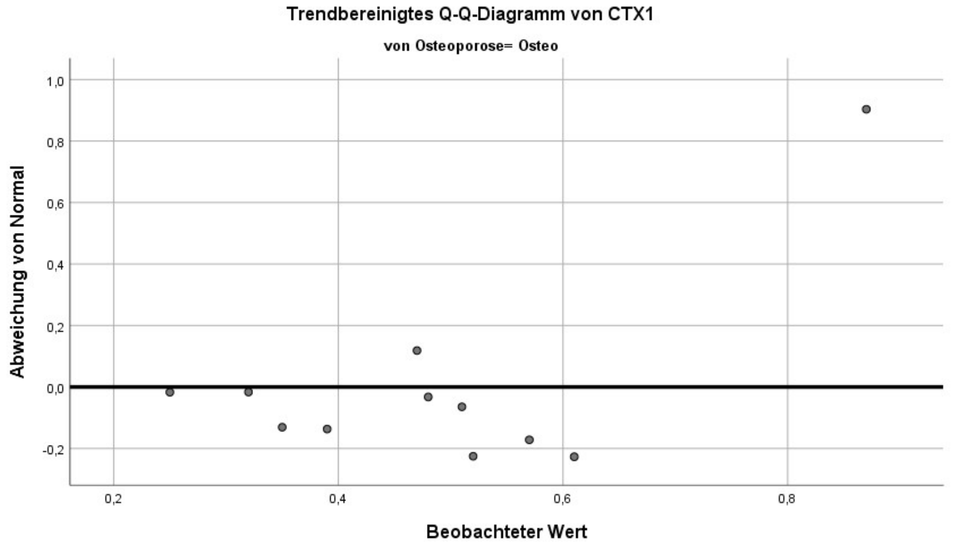

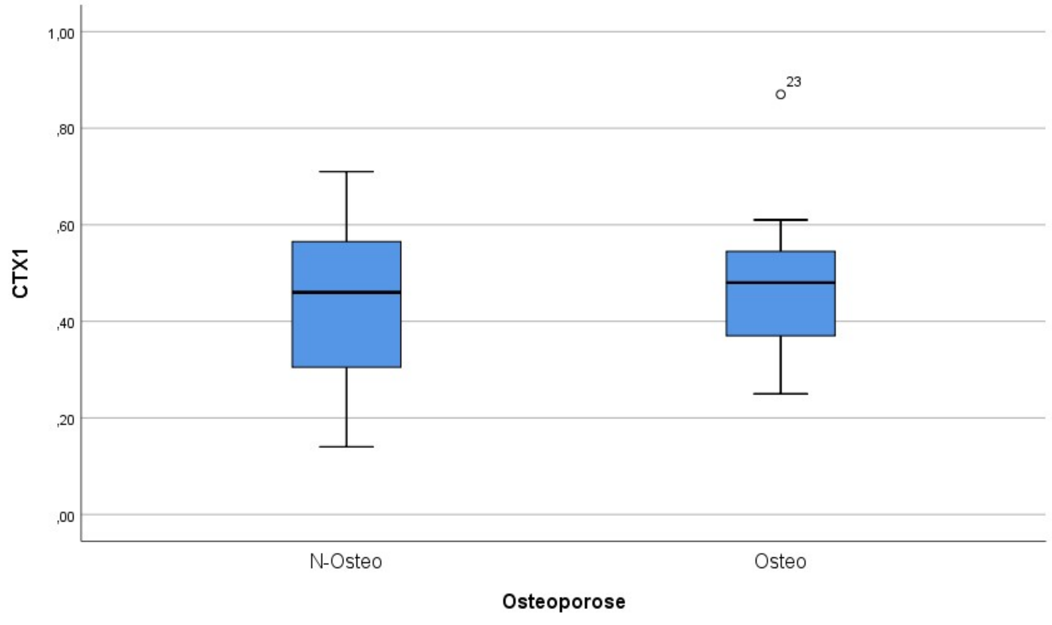

CTX2
Stengel-Blatt-Diagramme

```
CTX2 Stamm-Blatt-Diagramm für
Osteoporose= N-Osteo

 Häufigkeit  Stem &  Blatt

     1,00        0 .  1
     4,00        0 .  2333
     6,00        0 .  444455
     2,00        0 .  77
     2,00        0 .  89

 Stammbreite:      1,00
 Jedes Blatt:        1 Fälle
```

```
CTX2 Stamm-Blatt-Diagramm für
Osteoporose= Osteo

 Häufigkeit  Stem &  Blatt

     1,00        1 .  2
     5,00        2 .  12223
     2,00        3 .  78
     2,00        4 .  67
      ,00        5 .
     1,00        6 .  3

 Stammbreite:       ,10
 Jedes Blatt:        1 Fälle
```

Normalverteilote Q-Q-Diagramme

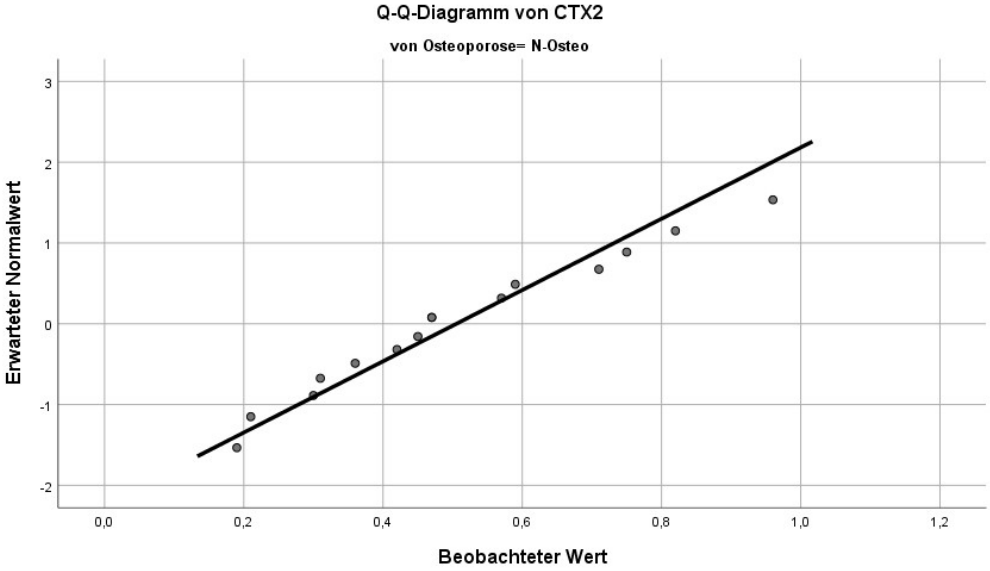

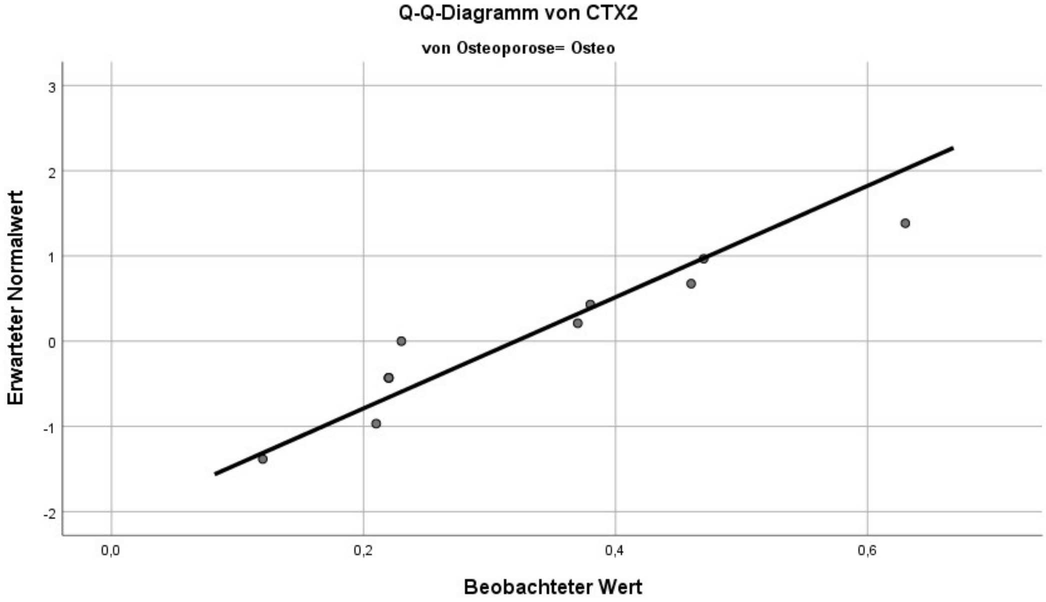

Trendbereinigte normalverteilte Q-Q-Diagramme

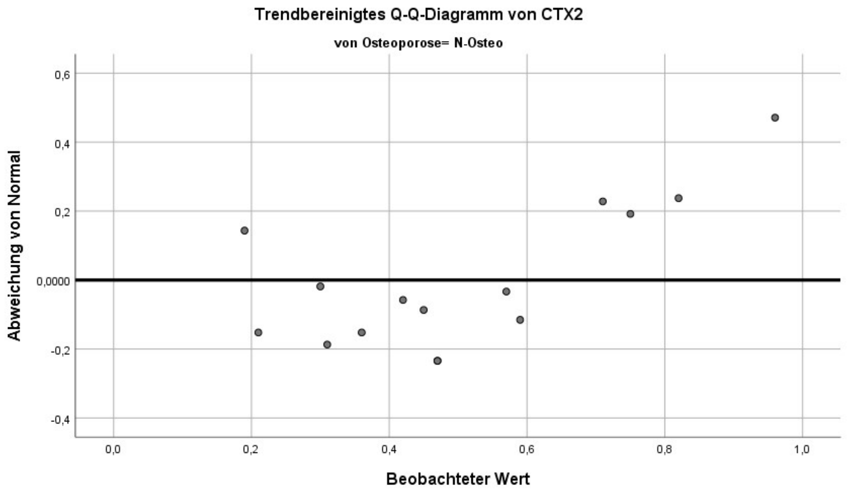

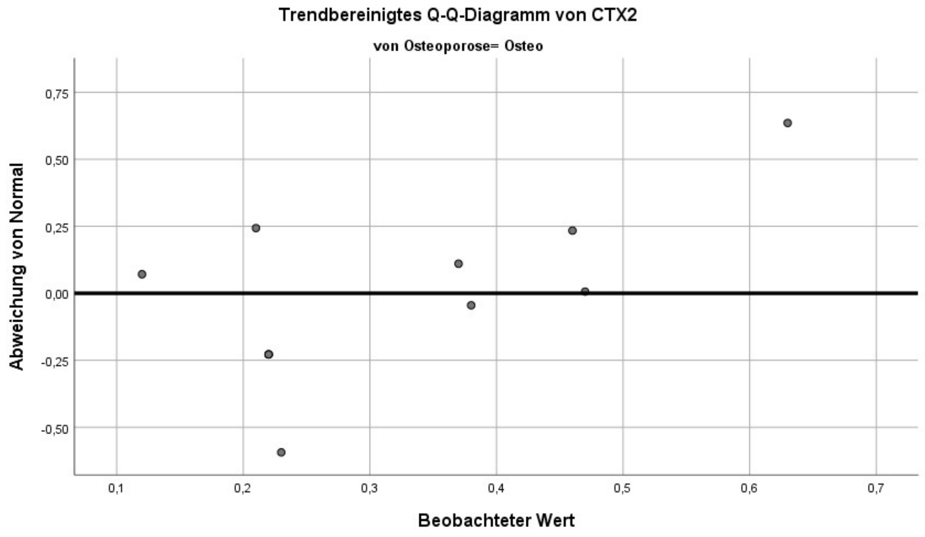

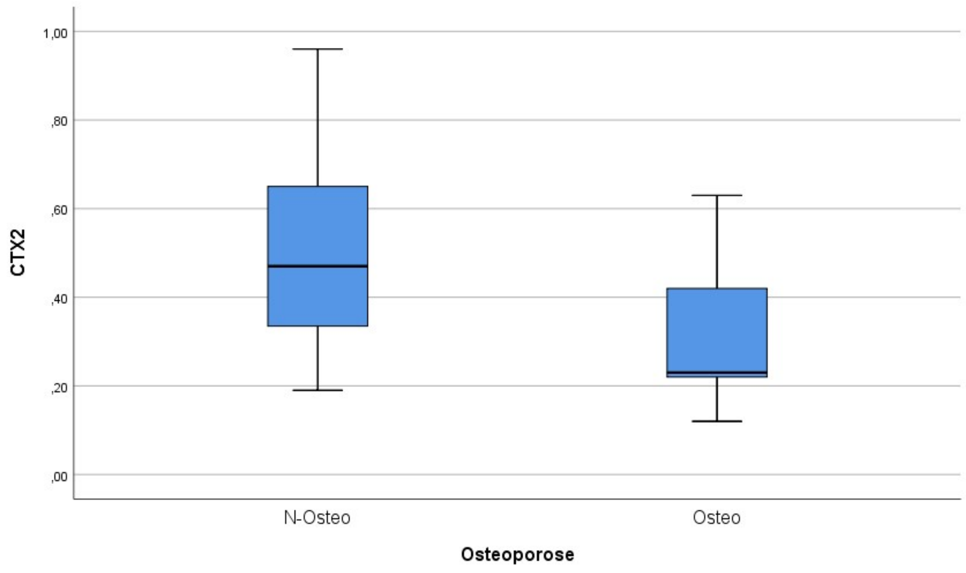

CTX3

Stengel-Blatt-Diagramme

```
CTX3 Stamm-Blatt-Diagramm für
Osteoporose= N-Osteo

 Häufigkeit  Stem &  Blatt

     6,00        0 .  233444
     8,00        0 .  55677778
     1,00        1 .  1

 Stammbreite:      1,00
 Jedes Blatt:        1 Fälle
```

```
CTX3 Stamm-Blatt-Diagramm für
Osteoporose= Osteo

 Häufigkeit  Stem &  Blatt

     1,00        1 .  8
     6,00        2 .  024699
     1,00        3 .  2
      ,00        4 .
     1,00        5 .  4
     1,00        6 .  9
     1,00 Extremwerte (>=,84)

 Stammbreite:       ,10
 Jedes Blatt:        1 Fälle
```

Normalverteilote Q-Q-Diagramme

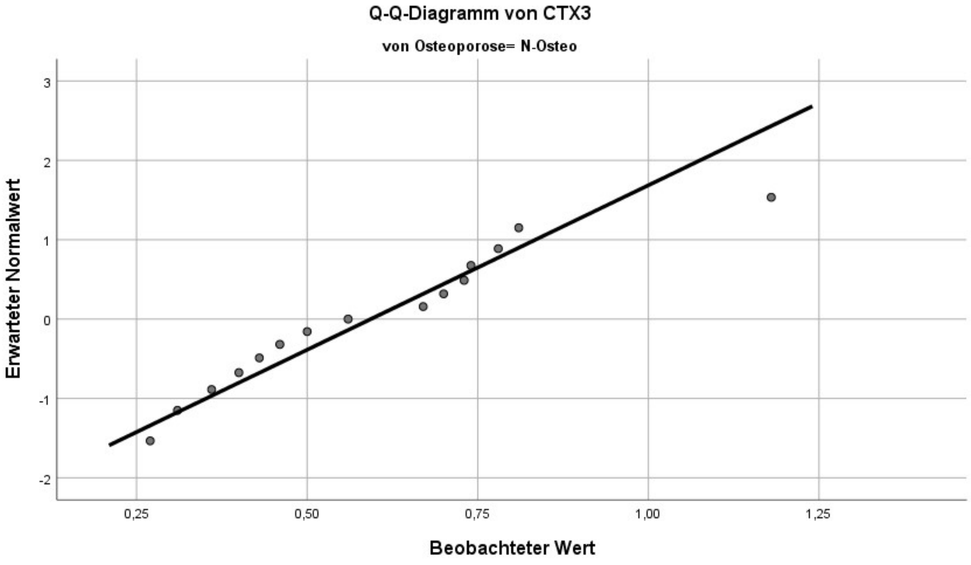

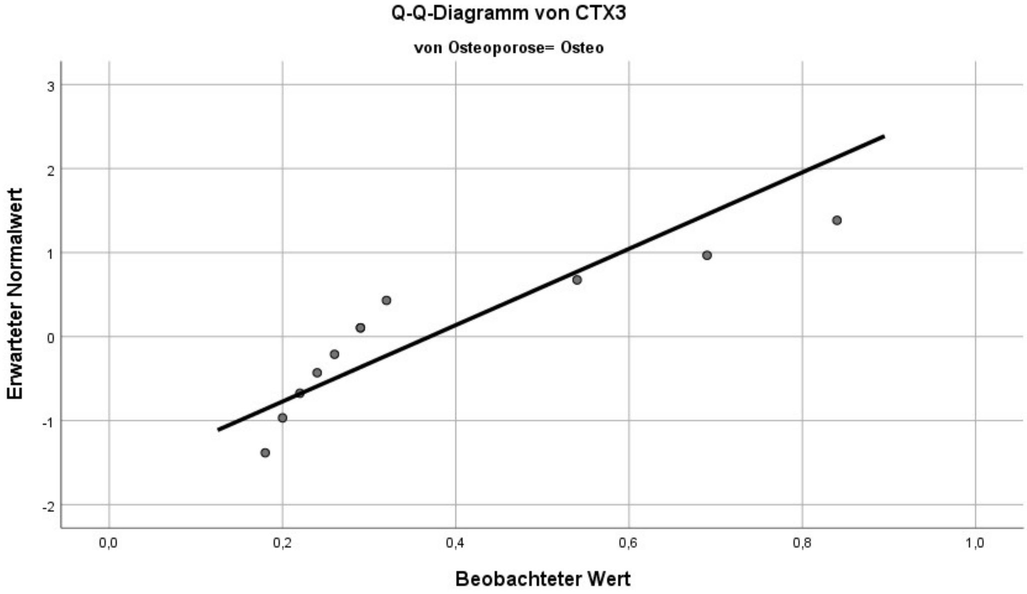

Trendbereinigte normalverteilte Q-Q-Diagramme

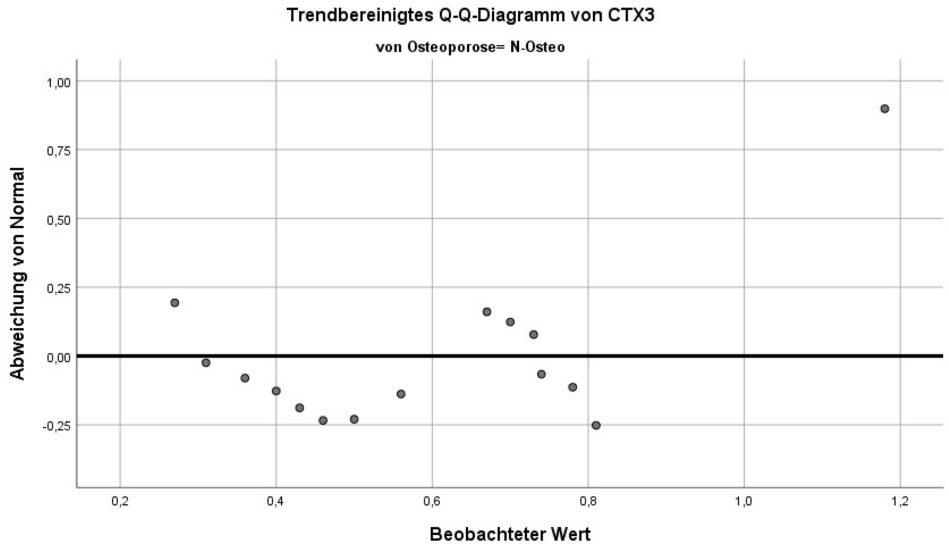

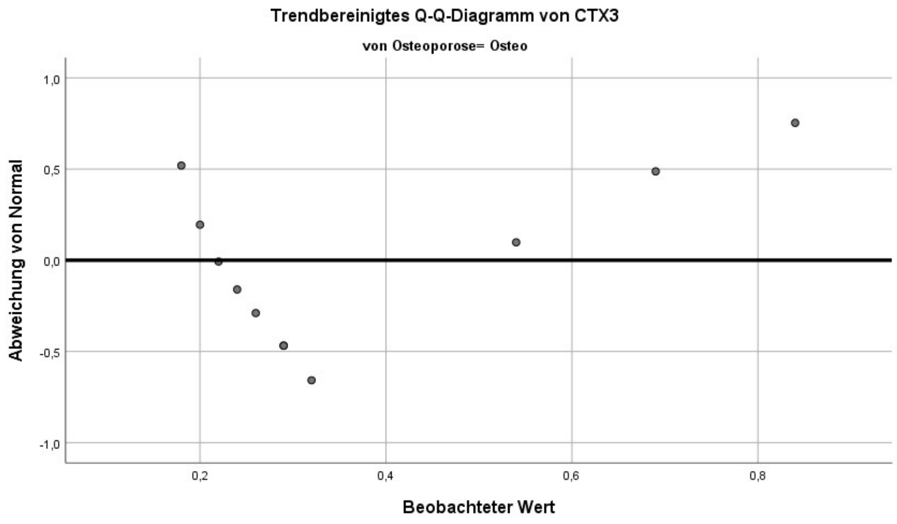

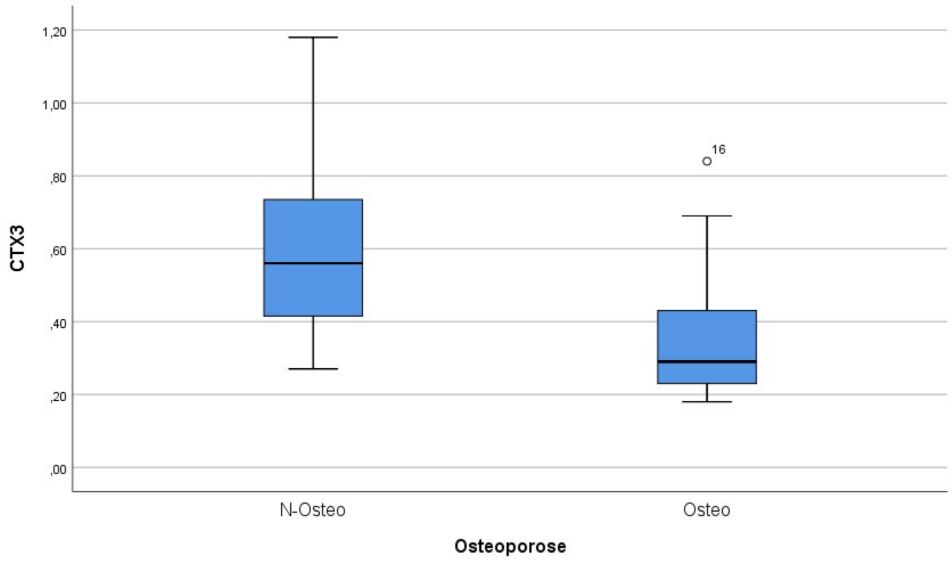

CTX4
Stengel-Blatt-Diagramme

CTX4 Stamm-Blatt-Diagramm für Osteoporose= N-Osteo	CTX4 Stamm-Blatt-Diagramm für Osteoporose= Osteo
Häufigkeit Stem & Blatt<br><br>4,00 0 . 2334<br>9,00 0 . 555556678<br>1,00 1 . 0<br>1,00 Extremwerte (>=1,3)<br><br>Stammbreite: 1,00<br>Jedes Blatt: 1 Fälle	Häufigkeit Stem & Blatt<br><br>3,00 0 . 011<br>3,00 0 . 233<br>4,00 0 . 4444<br>,00 0 .<br>1,00 0 . 8<br><br>Stammbreite: 1,00<br>Jedes Blatt: 1 Fälle

Normalverteilote Q-Q-Diagramme

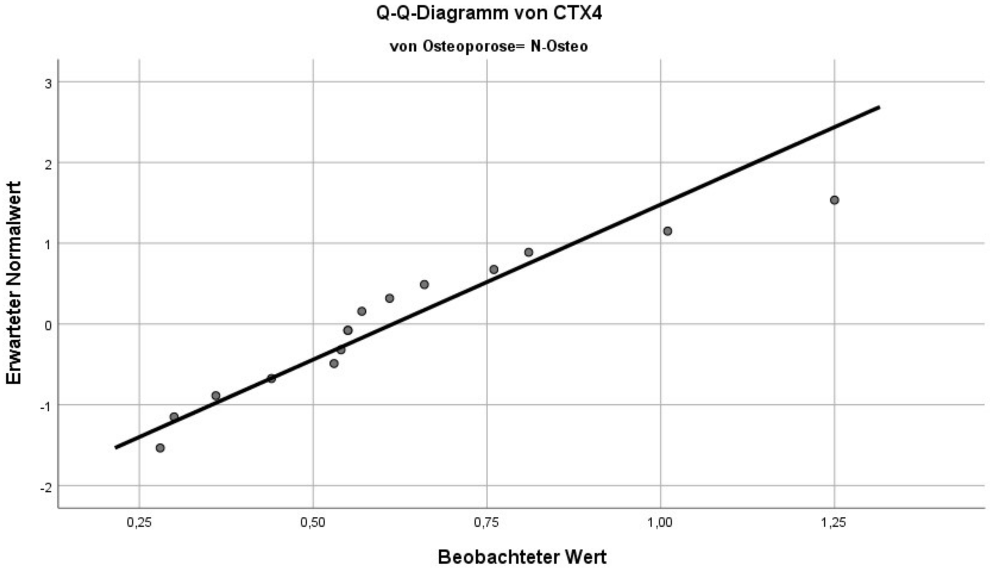

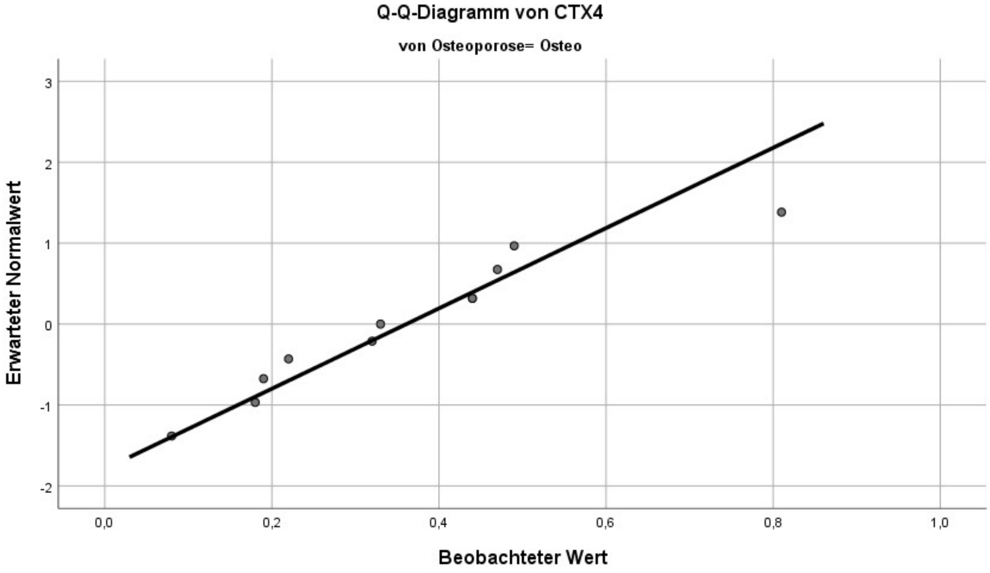

Trendbereinigte normalverteilte Q-Q-Diagramme

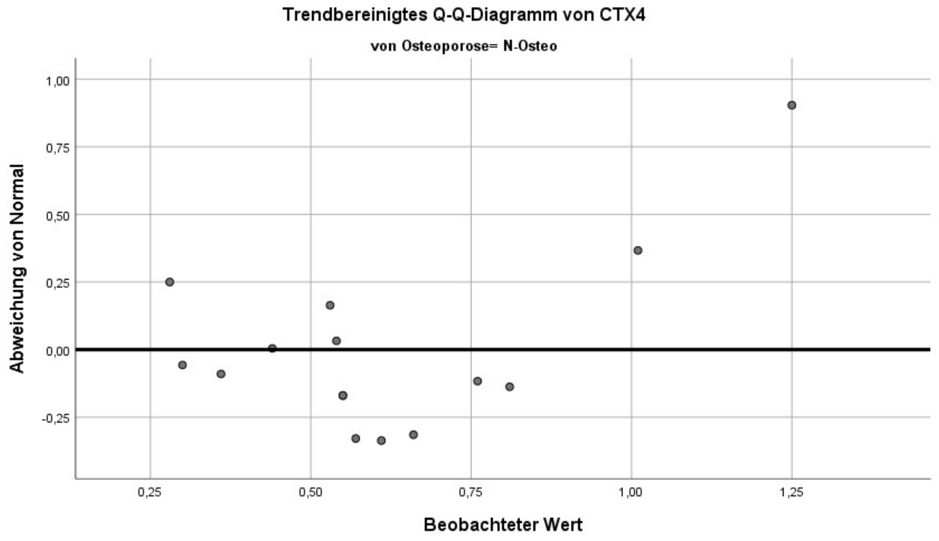

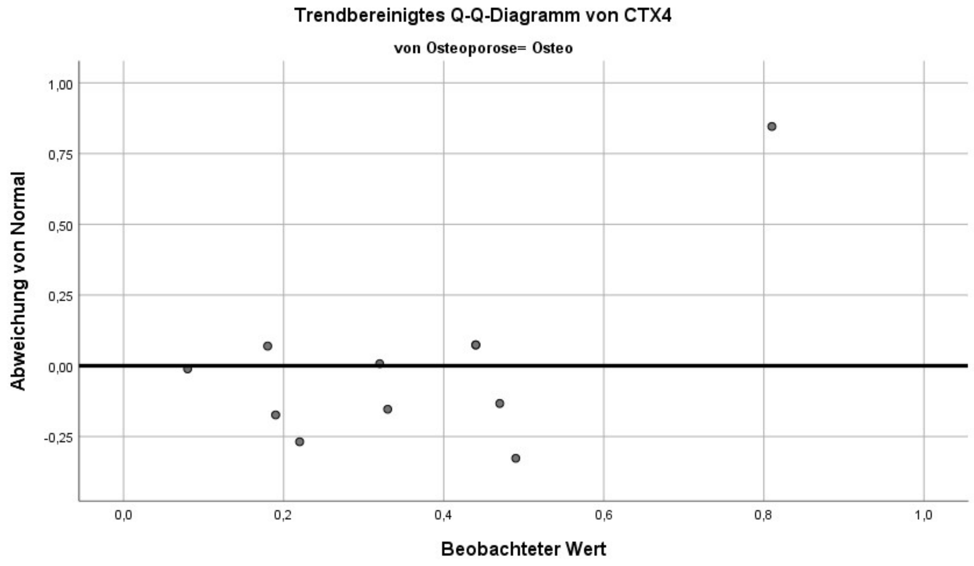

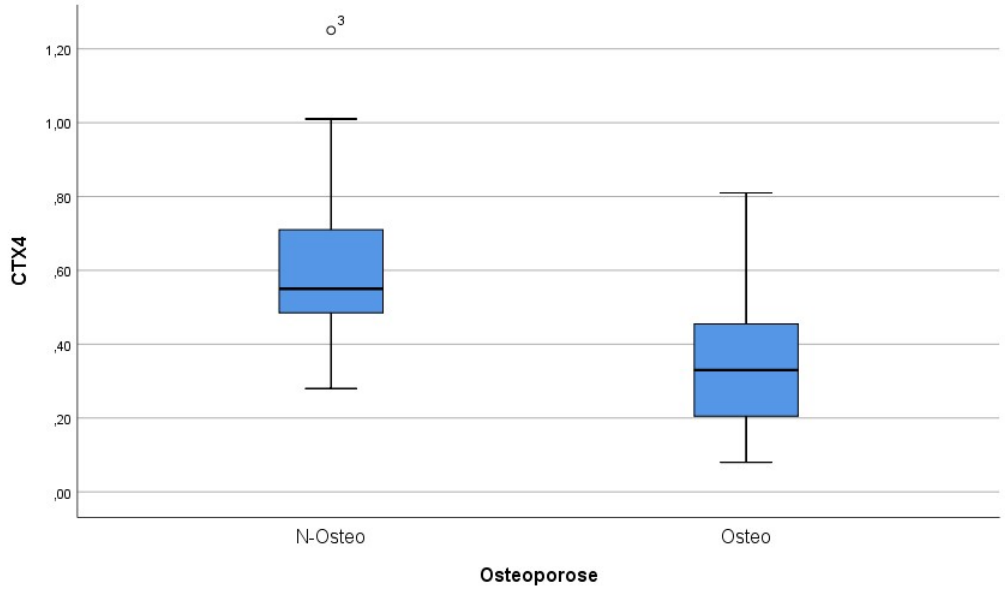

TGF1
Stengel-Blatt-Diagramme

```
TGF1 Stamm-Blatt-Diagramm für
Osteoporose= N-Osteo

 Häufigkeit  Stem &  Blatt

     2,00        2 .  37
     4,00        3 .  0279
     3,00        4 .  013
     5,00        5 .  03577
     1,00        6 .  1

 Stammbreite:     10000
 Jedes Blatt:        1 Fälle
```

```
TGF1 Stamm-Blatt-Diagramm für
Osteoporose= Osteo

 Häufigkeit  Stem &  Blatt

     2,00        3 .  09
     3,00        4 .  013
     5,00        5 .  03577
     1,00        6 .  1

 Stammbreite:     10000
 Jedes Blatt:        1 Fälle
```

Normalverteilote Q-Q-Diagramme

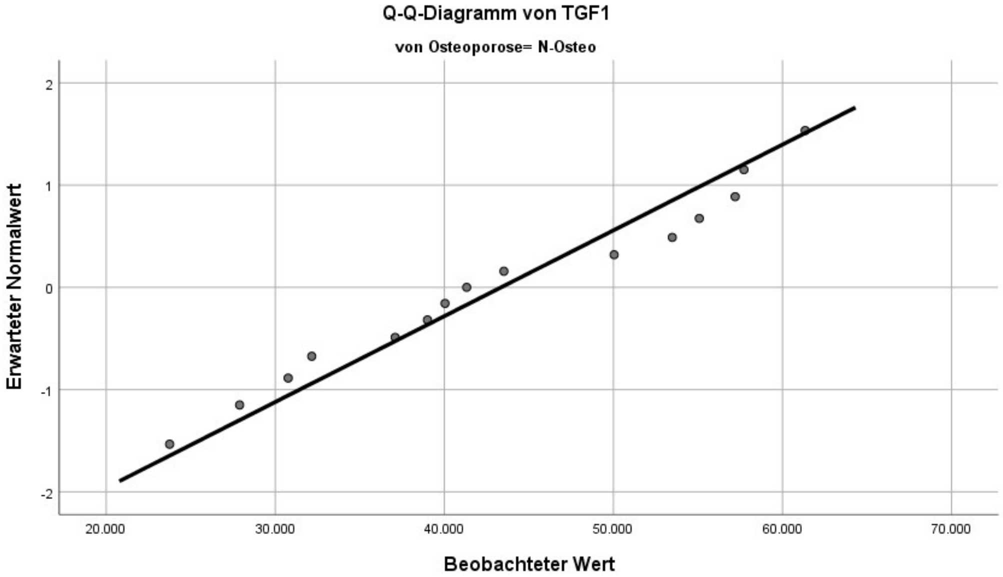

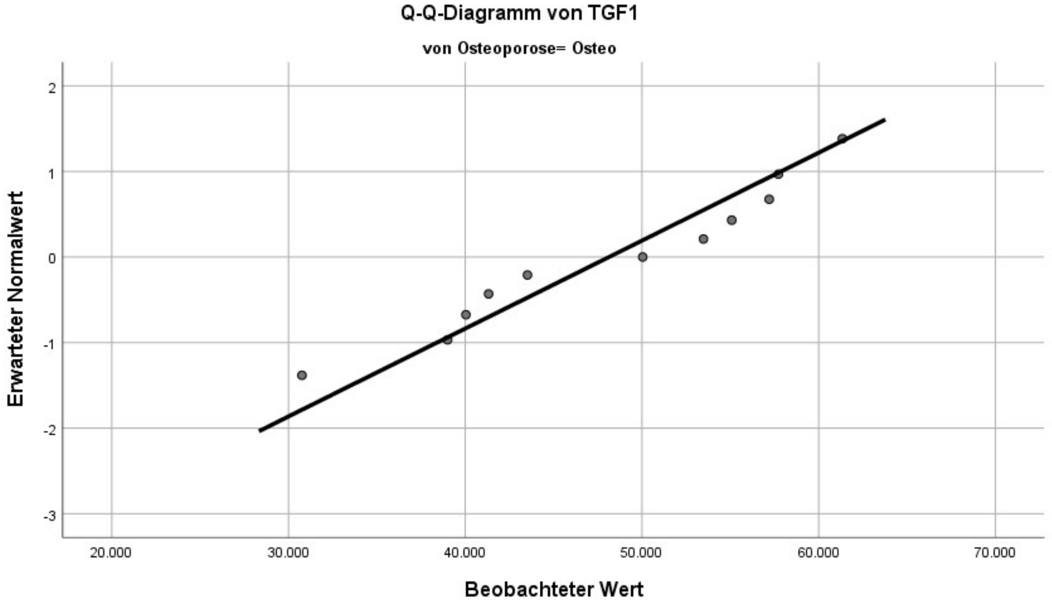

Trendbereinigte normalverteilte Q-Q-Diagramme

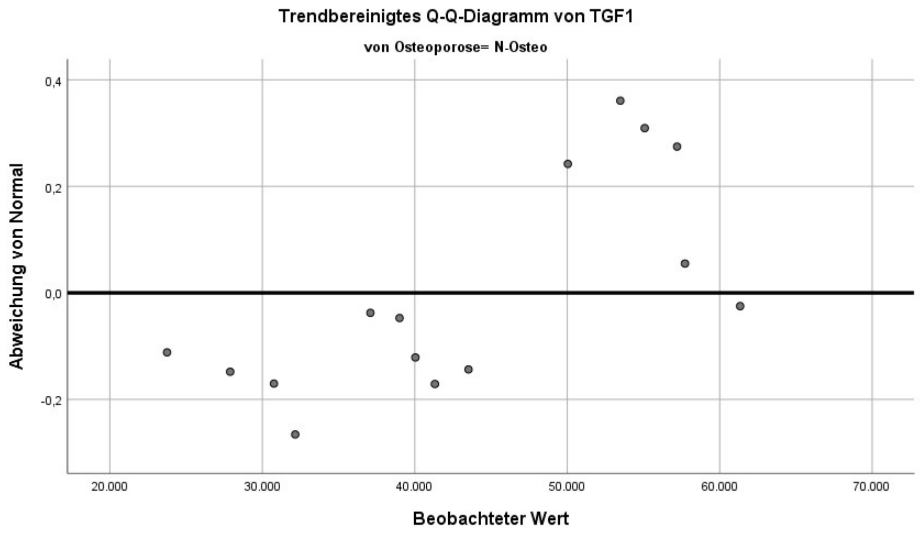

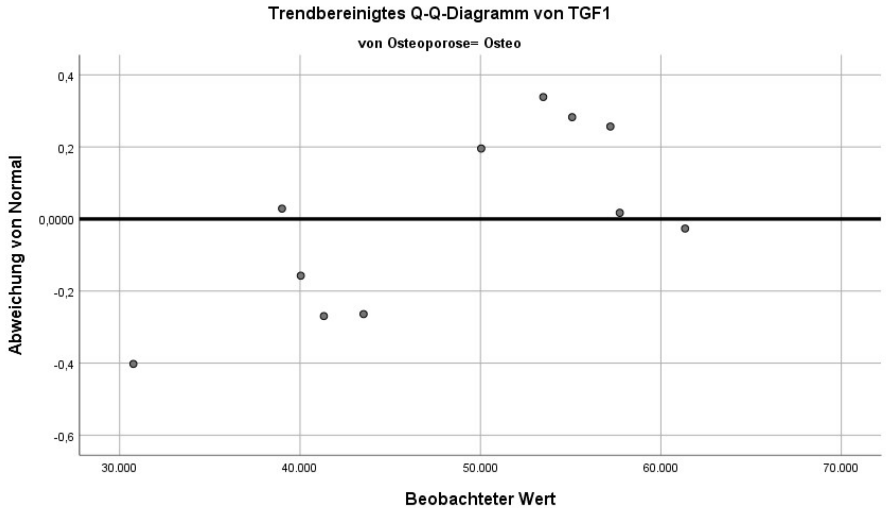

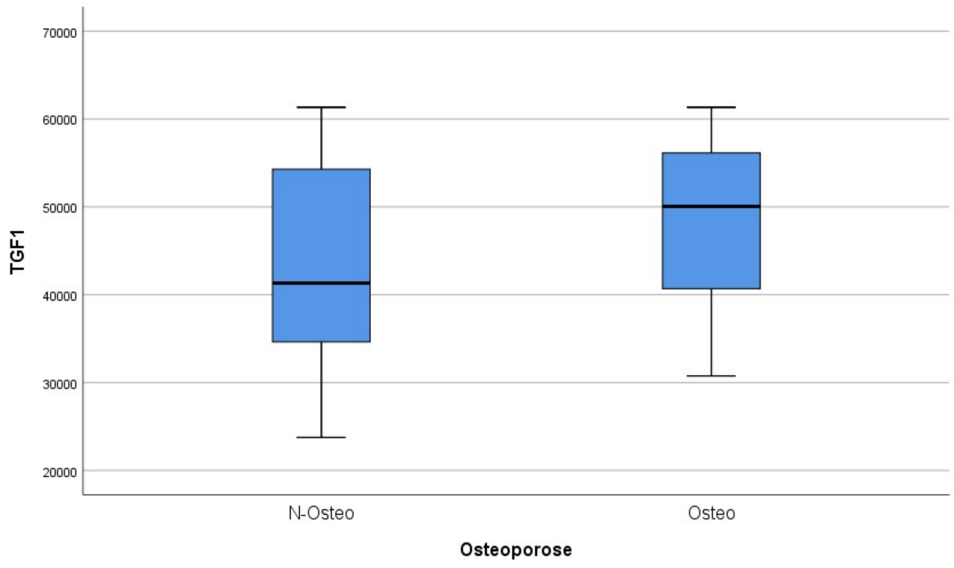

TGF2

Stengel-Blatt-Diagramme

```
TGF2 Stamm-Blatt-Diagramm für
Osteoporose= N-Osteo

 Häufigkeit   Stem &  Blatt

     2,00        2 .  89
     2,00        3 .  58
      ,00        4 .
     6,00        5 .  114566
     1,00        6 .  4
     3,00        7 .  257
     1,00 Extremwerte (>=104280)

 Stammbreite:     10000
 Jedes Blatt:        1 Fälle
```

```
TGF2 Stamm-Blatt-Diagramm für
Osteoporose= Osteo

 Häufigkeit   Stem &  Blatt

     1,00        3 .  8
      ,00        4 .
     5,00        5 .  14566
     1,00        6 .  4
     3,00        7 .  257
     1,00 Extremwerte (>=104280)

 Stammbreite:     10000
 Jedes Blatt:        1 Fälle
```

Normalverteilote Q-Q-Diagramme

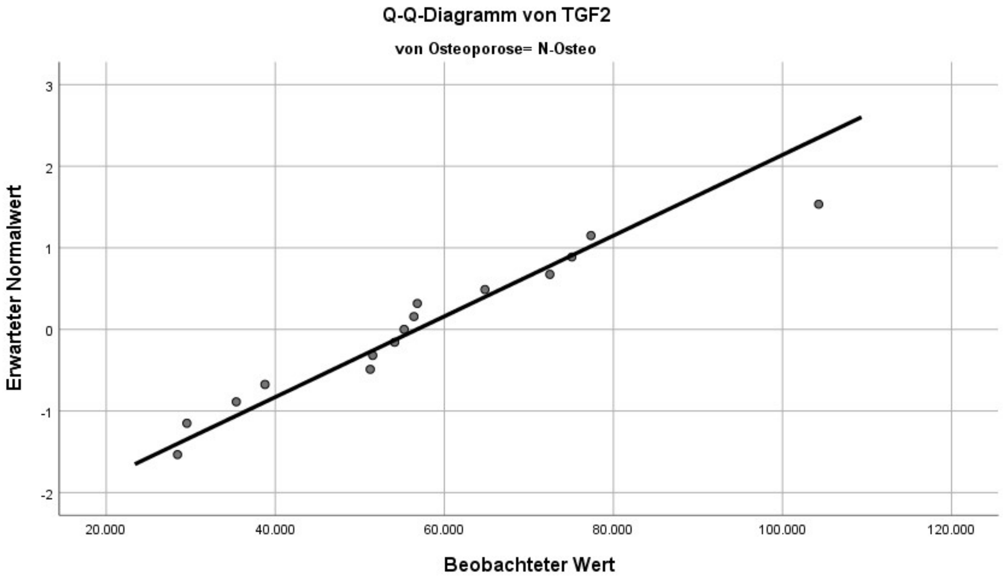

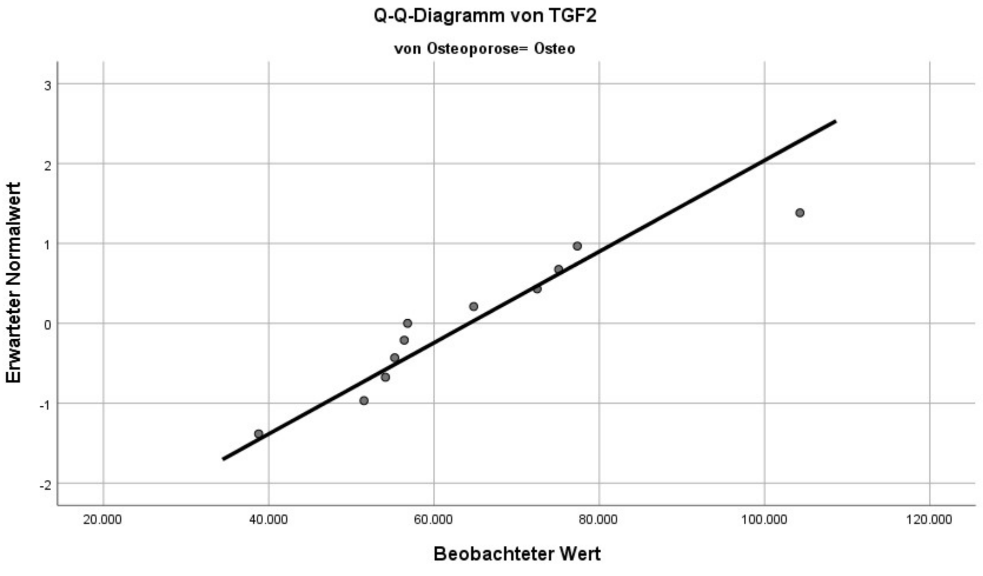

Trendbereinigte normalverteilte Q-Q-Diagramme

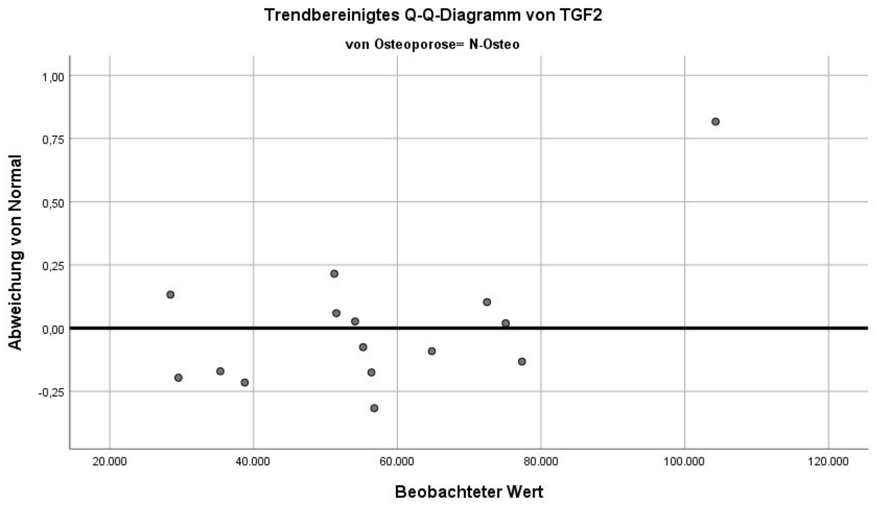

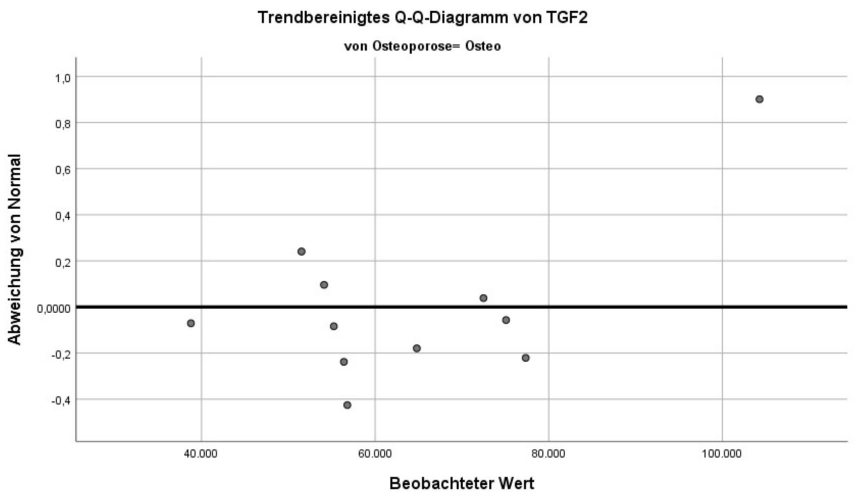

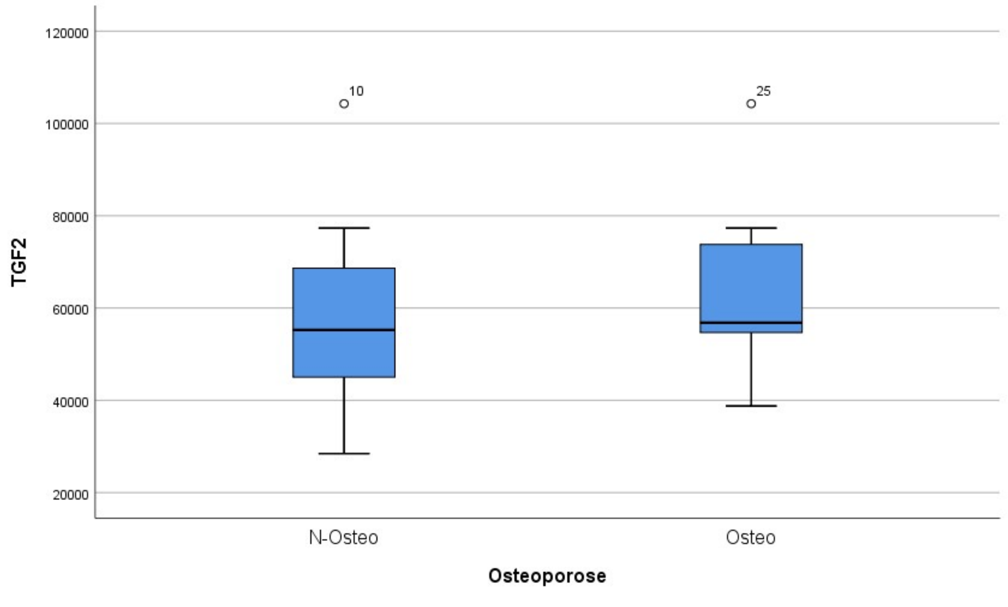

TGF3
Stengel-Blatt-Diagramme

```
TGF3 Stamm-Blatt-Diagramm für
Osteoporose= N-Osteo

 Häufigkeit   Stem &   Blatt

     1,00        2 .  7
     3,00        3 .  248
     2,00        4 .  78
     7,00        5 .  0123667
     1,00        6 .  3
     1,00 Extremwerte (>=87840)

 Stammbreite:  10000,00
 Jedes Blatt:        1 Fälle
```

```
TGF3 Stamm-Blatt-Diagramm für
Osteoporose= Osteo

 Häufigkeit   Stem &   Blatt

     1,00 Extremes    (=<32340)
     2,00        4 .  78
     4,00        5 .  0123
     2,00        5 .  67
     1,00        6 .  3
     1,00 Extremwerte (>=87840)

 Stammbreite:  10000,00
 Jedes Blatt:        1 Fälle
```

Normalverteilote Q-Q-Diagramme

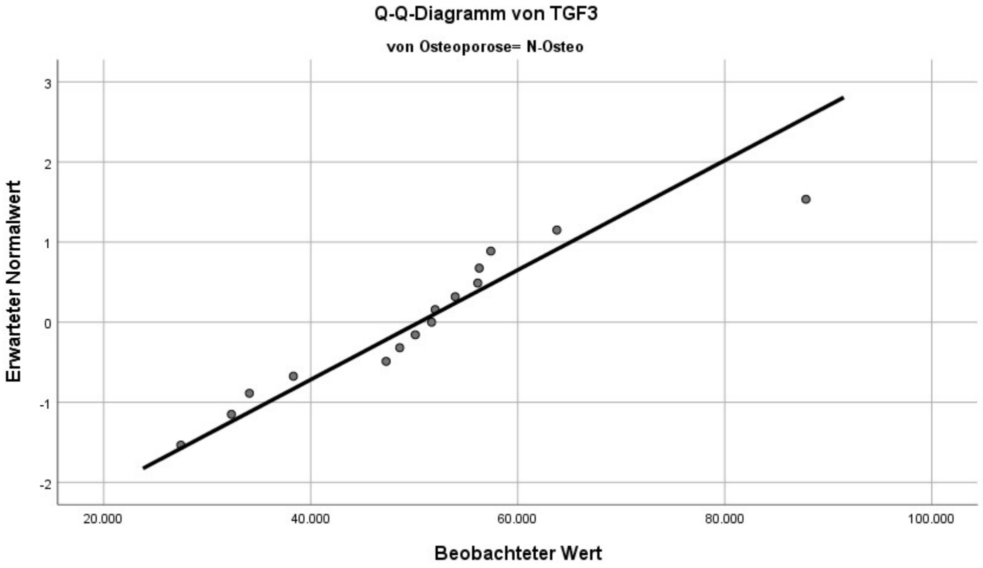

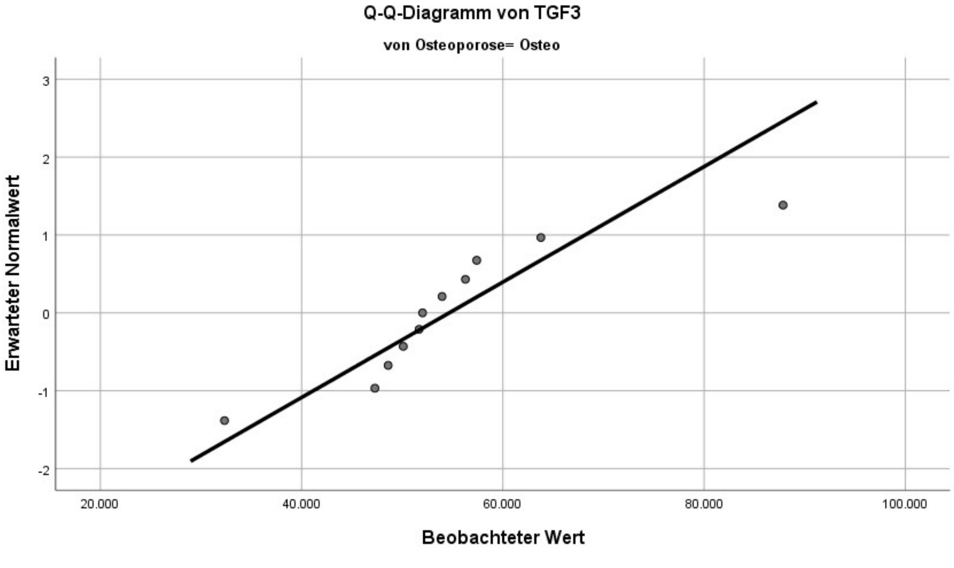

Trendbereinigte normalverteilte Q-Q-Diagramme

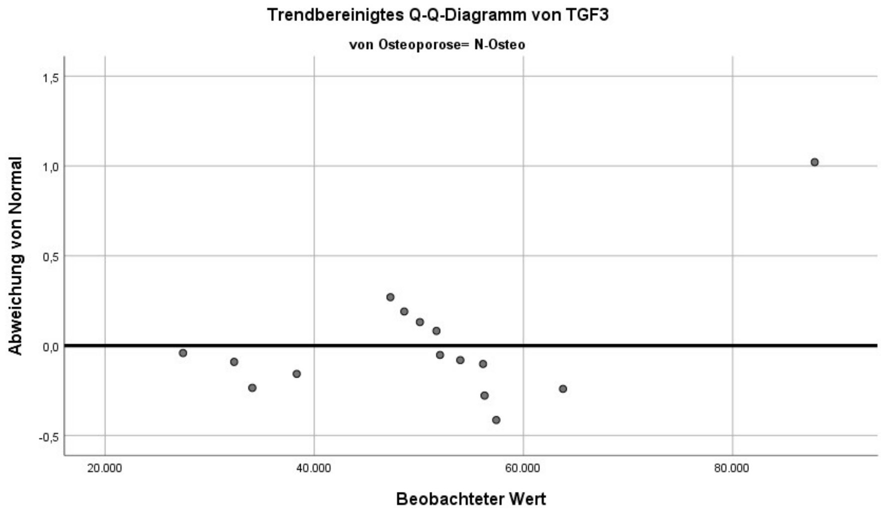

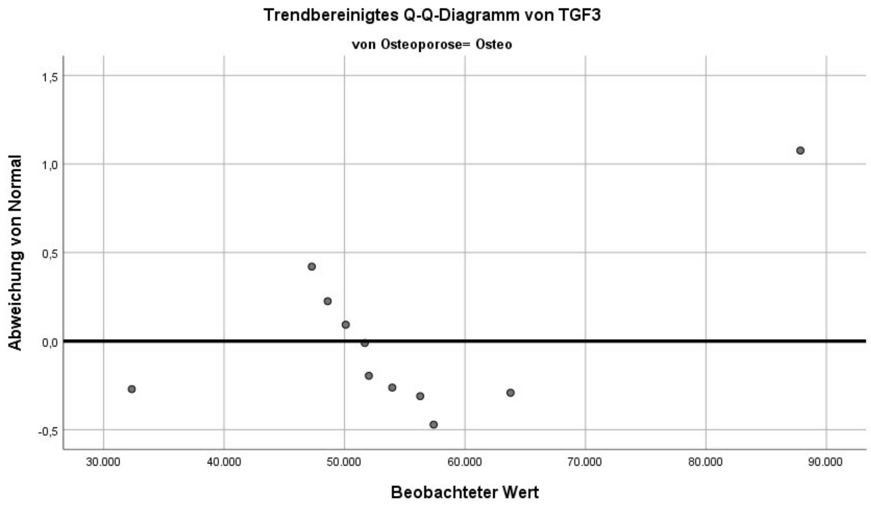

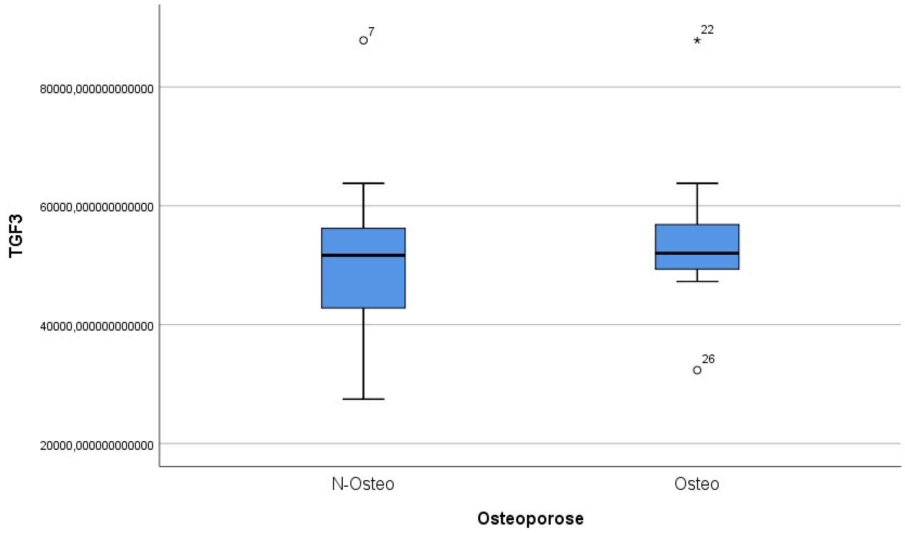

TGF4
Stengel-Blatt-Diagramme

```
TGF4 Stamm-Blatt-Diagramm für
Osteoporose= N-Osteo

 Häufigkeit  Stem &  Blatt

     1,00        2 .  7
     4,00        3 .  0568
     2,00        4 .  79
     4,00        5 .  1566
     4,00        6 .  1589

 Stammbreite:     10000
 Jedes Blatt:        1 Fälle
```

```
TGF4 Stamm-Blatt-Diagramm für
Osteoporose= Osteo

 Häufigkeit  Stem &  Blatt

     1,00        2 .  7
     1,00        3 .  8
     2,00        4 .  79
     3,00        5 .  166
     4,00        6 .  1589

 Stammbreite:     10000
 Jedes Blatt:        1 Fälle
```

Normalverteilote Q-Q-Diagramme

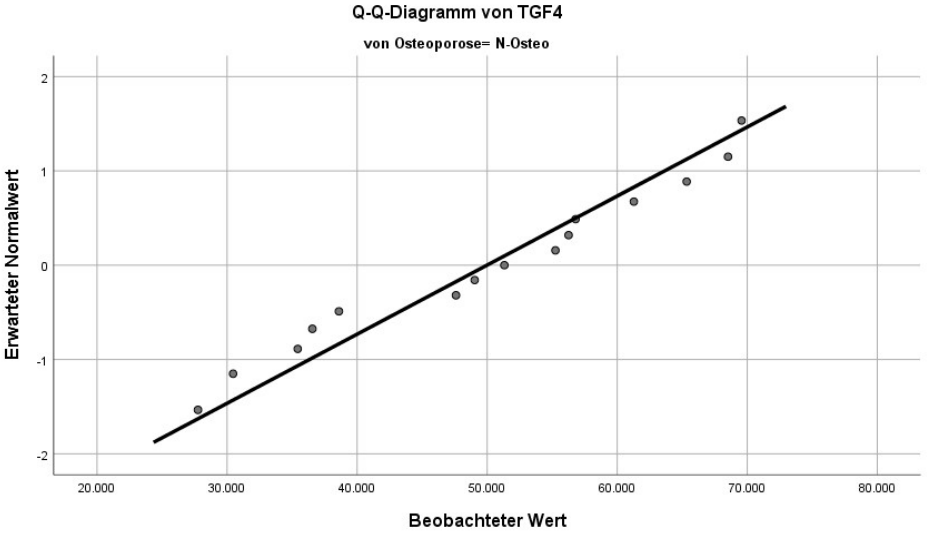

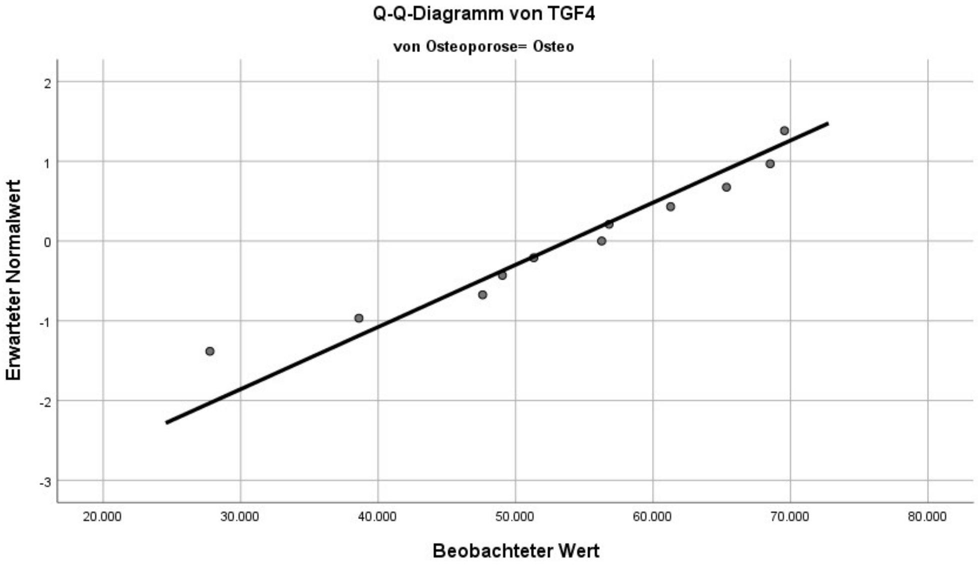

Trendbereinigte normalverteilte Q-Q-Diagramme

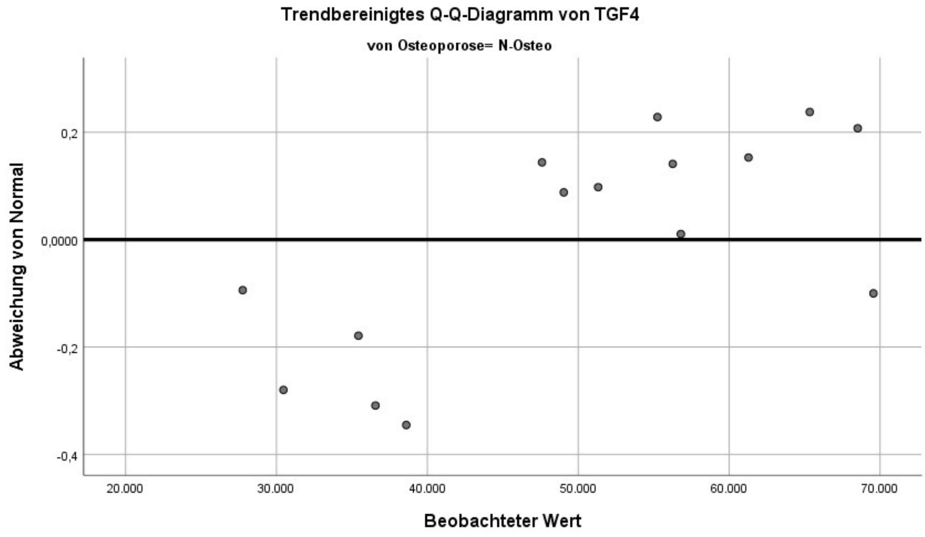

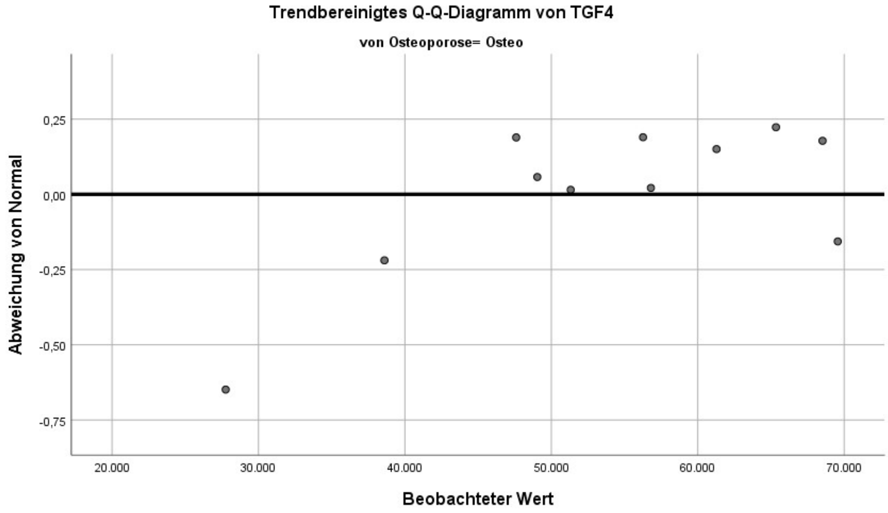

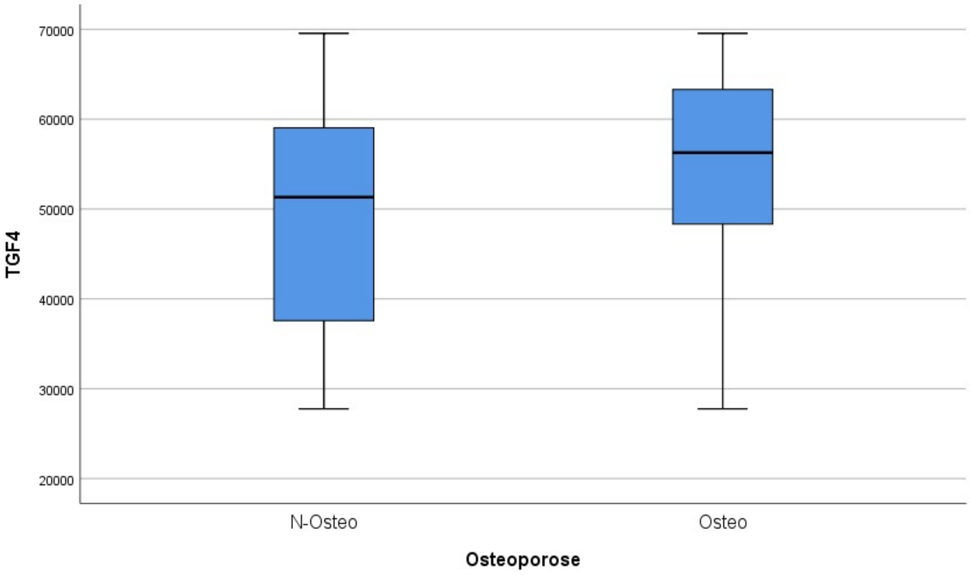

Supplement: S3 File — (PDF) [file pone.0270079.s003.pdf]
